# Supplementary material for: AAGGG repeat expansions trigger RFC1-independent synaptic dysregulation in human CANVAS neurons
Source: Sci Adv. 2024 Sep 4;10(36):eadn2321. doi: 10.1126/sciadv.adn2321 (PMC11373605; doi:10.1126/sciadv.adn2321)
Supplement: Supplementary file 2 [file sciadv.adn2321.v1.pdf]

NEUROSCIENCE

AAGGG repeat expansions trigger *RFC1*-independent synaptic dysregulation in human CANVAS neurons

Connor J. Maltby[1], Amy Krans[1,2], Samantha J. Grudzien[1,3,4], Yomira Palacios[1,5], Jessica Muiños[1,6], Andrea Suárez[1,5], Melissa Asher[1], Sydney Willey[1,3], Kinsey Van Deynze[4], Camille Mumm[4,7], Alan P. Boyle[4], Andrea Cortese[8,9], Alain Ndayisaba[10], Vikram Khurana[10,11], Sami J. Barmada[1], Anke A. Dijkstra[12,13], Peter K. Todd[1,2]*

Copyright © 2024 The Authors, some rights reserved; exclusive licensee American Association for the Advancement of Science. No claim to original U.S. Government Works. Distributed under a Creative Commons Attribution License 4.0 (CC BY).

Cerebellar ataxia with neuropathy and vestibular areflexia syndrome (CANVAS) is a recessively inherited neurodegenerative disorder caused by intronic biallelic, nonreference CCCTT/AAGGG repeat expansions within *RFC1*. To investigate how these repeats cause disease, we generated patient induced pluripotent stem cell–derived neurons (iNeurons). CCCTT/AAGGG repeat expansions do not alter neuronal *RFC1* splicing, expression, or DNA repair pathway function. In reporter assays, AAGGG repeats are translated into pentapeptide repeat proteins. However, these proteins and repeat RNA foci were not detected in iNeurons, and overexpression of these repeats failed to induce neuronal toxicity. CANVAS iNeurons exhibit defects in neuronal development and diminished synaptic connectivity that is rescued by CRISPR deletion of a single expanded AAGGG allele. These deficits were neither replicated by *RFC1* knockdown in control iNeurons nor rescued by RFC1 reprovision in CANVAS iNeurons. These findings support a repeat-dependent but RFC1 protein–independent cause of neuronal dysfunction in CANVAS, with implications for therapeutic development in this currently untreatable condition.

INTRODUCTION

Cerebellar ataxia with neuropathy and vestibular areflexia syndrome (CANVAS) is a recessively inherited, progressive, and debilitating disorder characterized by vestibular, cerebellar, and somatosensory impairments (*1*, *2*). CANVAS typically presents in late middle age, with most patients exhibiting progressive motor imbalance, oscillopsia, dysphagia, dysarthria, and peripheral sensory neuropathy. Postmortem analyses of patients with CANVAS indicate cerebellar and basal ganglia atrophy with diffuse Purkinje cell loss, as well as peripheral sensory neuronopathy including the vestibular, facial, and trigeminal nerves (*1*, *3*–*7*) (Fig. 1A).

CANVAS results from a biallelic, nonreference, pentameric CCCTT(AAGGG) repeat expansion in the second intron of replication factor complex subunit 1 (*RFC1*) and this same expansion also causes late-onset idiopathic ataxia and sensory neuropathy in isolation (*8*–*10*). Normally, this locus harbors a short $(AAAAG)_{\sim 11}$ repeat. However, CANVAS arises in individuals who have biallelic expansions at this locus above a currently denoted pathogenic threshold of >250 AAGGG repeats (*8*, *11*), and larger repeat sizes may correlate with an earlier age of onset (*11*). Individuals harboring a heterozygous $(AAGGG)_{exp}$ allele with the wild-type (WT) $(AAAAG)_{\sim 11}$ allele do not develop CANVAS spectrum phenotypes, consistent with a recessive pattern of inheritance (*8*, *9*). Since the initial description of AAGGG expansions at this locus, several further pathogenic repeat expansion conformations have been described: ACAGG expansions have been identified in patients with CANVAS of Oceania and East Asian descent (*12*), and more recently, the 100,000 genomes project database has identified AAGGC, AGGGC, and AGAGG expansions, either in the homozygous or compound heterozygous state with the AAGGG expansion (*13*). Furthermore, AAAGG repeat expansions previously reported to be nonpathogenic in the homozygous state and compound heterozygous state with AAGGG expansions have since been found to be pathogenic when present at substantially expanded states (*13*). *RFC1*-associated repeat expansions are common, with a minor allelic frequency of ~0.7 to 6.8% and an expected homozygous pathogenic repeat population frequency of 1 in 625 individuals worldwide—making it one of the most common causes of inherited ataxia and sensory neuropathy (*8*, *14*, *15*).

Despite clinical interest in CANVAS, the mechanisms by which this repeat expansion causes pathogenesis and neuronal death are unknown. Initial studies suggested that *RFC1* mRNA and protein expression are unaltered in the context of the repeat expansion, which is inconsistent with the classical mechanism by which recessively inherited repeat expansions elicit a loss of function for the genes in which they reside (*8*, *10*, *16*, *17*). However, identification of rare compound heterozygotic CANVAS patients harboring *RFC1* truncating and splicing loss-of-function mutations with single-allele repeat expansions suggest a role for RFC1 protein function in CANVAS (*18*–*20*). Alternatively, the repeats could potentially elicit toxicity through dose-dependent gain-of-function mechanisms [such as repeat associated non-AUG initiated (RAN) translation or repeat RNA-protein complex formation] that only manifest in homozygosity or in combination with RFC1 haploinsufficiency (*21*, *22*) (Fig. 1A). Limited postmortem and cell-based analyses performed to date have not demonstrated classic pathological

[1]Department of Neurology, University of Michigan, Ann Arbor, MI, USA. [2]Ann Arbor Veterans Administration Healthcare, Ann Arbor, MI, USA. [3]Neuroscience Graduate Program, University of Michigan, Ann Arbor, MI, USA. [4]Department of Computational Medicine and Bioinformatics, University of Michigan, Ann Arbor, MI, USA. [5]Postbaccalaureate Research Education Program, University of Michigan, Ann Arbor, MI, USA. [6]UM SMART Undergraduate Summer Program, University of Michigan, Ann Arbor, MI, USA. [7]Department of Human Genetics, University of Michigan, Ann Arbor, MI, USA. [8]Department of Neuromuscular Diseases, UCL Queen Square Institute of Neurology, London WC1N 3BG, UK. [9]Department of Brain and Behaviour Sciences, University of Pavia, Pavia 27100, Italy. [10]Department of Neurology, Brigham and Women's Hospital and Harvard Medical School, Boston, MA, USA. [11]Harvard Stem Cell Institute, Broad Institute of MIT and Harvard, Cambridge, MA, USA. [12]Department of Pathology, Amsterdam UMC, Amsterdam Neuroscience, Amsterdam, Netherlands. [13]Swammerdam Institute for Life Sciences, University of Amsterdam, Amsterdam, Netherlands.
*Corresponding author. Email: petertod@med.umich.edu

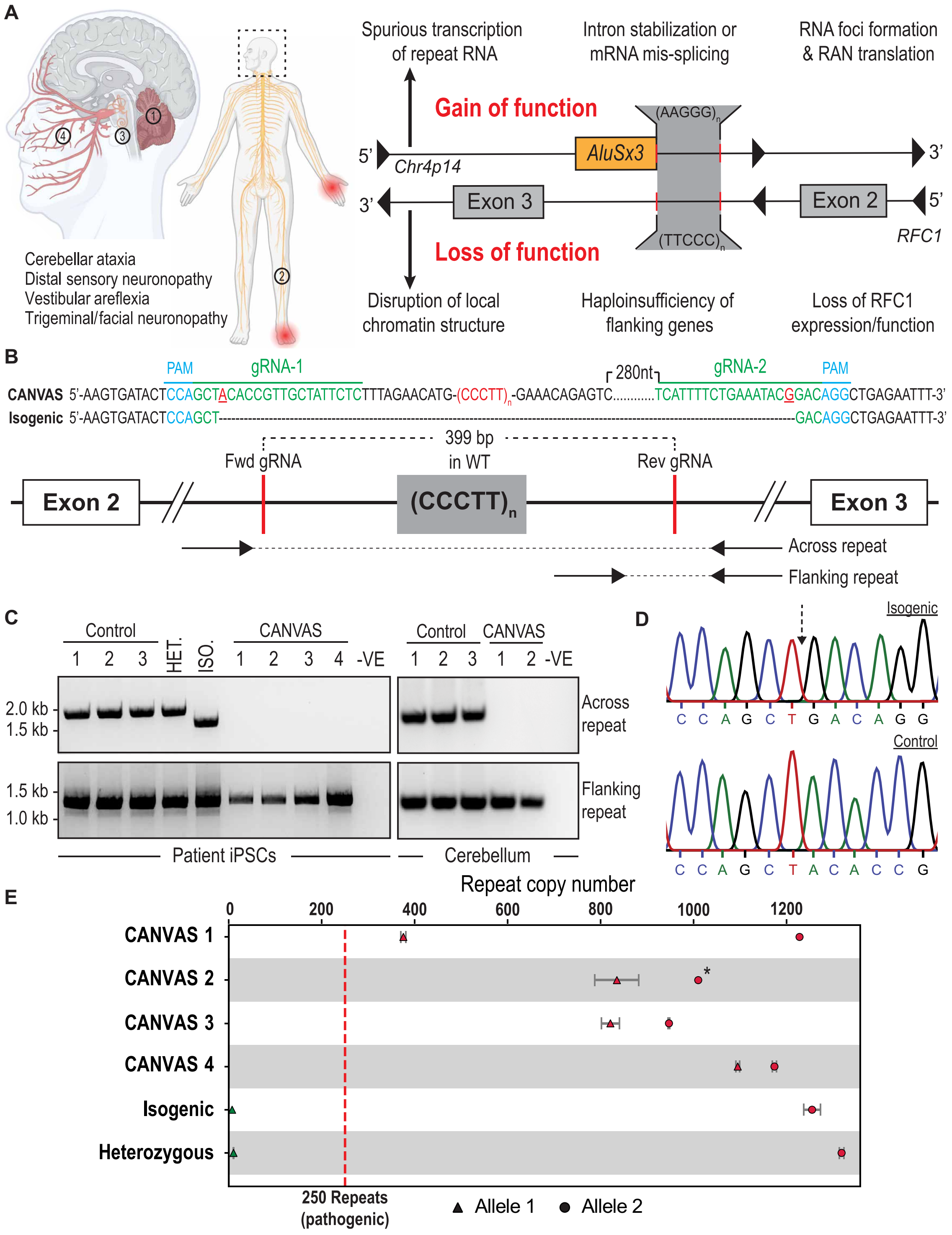

Fig. 1. Repeat characterization and heterozygous correction of CANVAS patient–derived iPSC lines. (**A**) Schematic of brain, central and peripheral nervous system regions affected in CANVAS (left), and potential mechanisms of repeat toxicity in CANVAS (right). (**B**) Repeat architecture of the expanded locus and CRISPR gRNA design to remove the AAGGG/CCCTT repeat expansion by nonhomologous end joining (NHEJ). (**C**) Endpoint PCR of gDNA extracted from CANVAS patient– and control-derived iPSC lines and CANVAS and control cerebellum tissue utilizing the primer pair outlined in (B) to screen for the presence of WT repeat, mutant repeat expansion, or deletion of expanded repeat. (**D**) Chromatogram of Sanger sequencing identifying AAGGG/CCCTT allele deletion in heterozygous isogenic line indicating the expected NHEJ join point compared to the control iPSC line. (**E**) Schematic outlining the repeat copy number per allele for each of the patient-derived iPSC lines used as identified by Oxford Nanopore gDNA targeted long-read sequencing. Green, sub-pathogenic repeat length; Red, pathogenic repeat length. Error bars indicate confidence in exact copy number calls. The "*" indicates lower boundary for repeat copy number from the longest read observed due to the absence of reads spanning the full repeat.

hallmarks seen in other repeat expansion disorders, such as repeat RNA foci or ubiquitinated inclusions (*8*).

Understanding the pathogenic nature of this repeat expansion is further complicated by its manifestation within two distinct genetic elements on opposing strands. The AAGGG repeat sits at the 3′ end of an *AluSx3* transposable element on the sense strand, while the complementary CCCTT repeat is embedded deep within the large intron 2 region of *RFC1*, potentially allowing for two distinct and context-dependent mechanisms of toxicity. *Alu* elements are ancient retro-transposition artifacts that make up roughly 10% of the human genome (~1 million copies) (*23*) and serve as reservoirs of potential regulatory functions that have actively driven primate evolution, with evidence to suggest roles for intronic *Alu* elements in mRNA splicing (*24*, *25*) (constitutive and alternative), RNA editing (*26*), and protein translation (*27*). Moreover, the AAGGG repeat sequence found on the opposing strand to *RFC1* at this *AluSx3* 3′ end is predicted to form stable G-quadruplex secondary structures that have been implicated in other repeat expansion disease pathogenesis for both transcribed RNAs and DNA during transcription and DNA replication fork formation (*28*).

Given the paucity of empiric data on these repeats and the multiple potential mechanisms underpinning CANVAS, we investigated how these repeats cause disease, including independent evaluation of previously tested ideas and direct measures of the ability of these repeats to elicit neurotoxicity. We generated multiple CANVAS patient-derived and CRISPR-corrected isogenic induced pluripotent stem cell (iPSC) lines and differentiated them into neurons. Through functional neuronal assays, transcriptomic analyses, and in vitro toxicity investigations, we identify deficits in key neuronal cellular pathways in CANVAS neurons that are rescued upon CRISPR correction of a single expanded repeat allele. In contrast, loss of RFC1 expression fails to recapitulate these cellular and molecular phenotypes and ectopic expression of RFC1 in CANVAS iNeurons is insufficient to reverse pathologic cascades. Together, these studies support a repeat-dependent mechanism of toxicity that operates outside of the canonical functions of the RFC1 protein.

RESULTS

CANVAS patient–derived and CRISPR-corrected heterozygous isogenic iPSC lines

Homozygous nonreference repeat expansions in *RFC1* could potentially elicit neurodegeneration through multiple gain-of-function or loss-of-function mechanisms (Fig. 1A). To assess these possibilities systematically, we generated a series of CANVAS patient–derived iPSC lines from four patients and three controls, as well as one family member with a heterozygous *RFC1* expansion (fig. S1, A and B). As an additional experimental control, we generated an isogenic line through deletion of the expanded allele in patient 1 iPSC line through CRISPR-Cas9 genome editing (Fig. 1, B to D). Successful deletion of a single expanded allele was achieved in CANVAS patient 1 iPSCs (Fig. 1C), and the specificity of this deletion was confirmed by Sanger sequencing of PCR products (Fig. 1D). However, we were unable to generate biallelic homozygous deletions of the *AluSx3* element and expanded repeat despite multiple rounds of editing and re-editing in both CANVAS and control cell lines, consistent with prior reports (*29*). As such, the heterozygous deletion line was taken forward for experimental analysis. Cell lines were confirmed to have AAGGG/CCCTT repeat expansion alleles by repeat-primed PCR (fig. S1A) and sized by nanopore sequencing (*30*) in combination with the results obtained by screening PCR (Fig. 1E). CANVAS and control iNeurons generated through a dual-SMAD differentiation protocol exhibited comparable neuronal numbers and efficiencies for differentiation as measured by NeuN-positive nuclei and MAP2 expression (fig. S1E).

Translated AAGGG repeat products are detected in brains of patients with CANVAS

To investigate the potential of repeat-dependent gain-of-function mechanisms in CANVAS pathogenesis (Fig. 1A), we generated repeat-containing reporter constructs encompassing various repeat motifs within intronic sequence contexts (Fig. 2A). WT AAAAG/CTTTT and mutant CANVAS AAGGG/CCCTT repeat expansions were generated by recursive directional ligation (RDL) (*31*) and were ligated into plasmid backbones containing 150 bp of upstream intronic sequence deriving from the genomic strand within which the repeat motif would be found; AAAAG/AAGGG repeats for the sense strand *AluSx3*-containing sequence and CTTTT/CCCTT repeats for the antisense *RFC1* intron 2–containing sequence.

We first assessed whether expression of these repeat-containing constructs elicit RNA foci formation by using repeat-targeting fluorescent probes and RNA HCR-FISH (Fig. 2B and fig. S2A). Prior studies have shown mixed results in regard to whether RNA foci occur in human CANVAS tissues, with discrepancies between studies potentially reflecting technical differences or repeat motif effects (*8*, *32*). In human embryonic kidney (HEK) 293 cells, sense (AAGGG) repeat-directed probes detected nuclear and cytoplasmic RNA foci that colocalized with anti-nanoluciferase probes in transfected cells and generated patterns of expression that were different from nanoluciferase expression alone. Consistent with some binding of the probes to the repeat as DNA, nuclear foci, but not cytoplasmic foci, were reduced with DNase treatment (fig. S2A). However, much of the nuclear and all the cytoplasmic signal was ablated by RNase treatment (fig. S2A). A similar pattern was seen for antisense (CCCTT) repeat-directed probes, suggesting that both the sense and antisense pentanucleotide repeats can form RNA condensates within cells. To assess whether such foci are detectable in patient neurons, we performed RNA HCR-FISH with these same probes in control ($n = 3$) and CANVAS ($n = 3$) patient iPSC–derived neurons (Fig. 2B and fig. S2, B and C). No significant difference was observed between CANVAS and control neurons for sense AAGGG repeat-RNA foci detection ($P = 0.42$). In contrast, antisense CCCTT repeat-RNA foci were detected in CANVAS neurons at greater rates compared to control cells ($P = 0.04$). However, the overall abundance of foci was low, and the specificity was imperfect, with 8.94% of control neurons and 11.3% of CANVAS neurons showing antisense CCCTT RNA foci in rater-blinded assays (Fig. 2B and fig. S2D).

The native sequences surrounding the repeats are predicted to contain both AUG and non-AUG near-cognate codons upstream of the repeats without any intervening stop codons—potentially placing the repeats within open reading frames or making them subject to RAN translation (*21*, *22*). To assess whether these repeats might be translated in patients, we generated intronic sense and antisense reporter constructs with Nanoluciferase-3×Flag (NL-3F) C-terminal tags in all three potential reading frames. Protein expression and nanoluciferase activity analysis in transfected HEK293 cells showed selective translation of repeat-derived peptides within

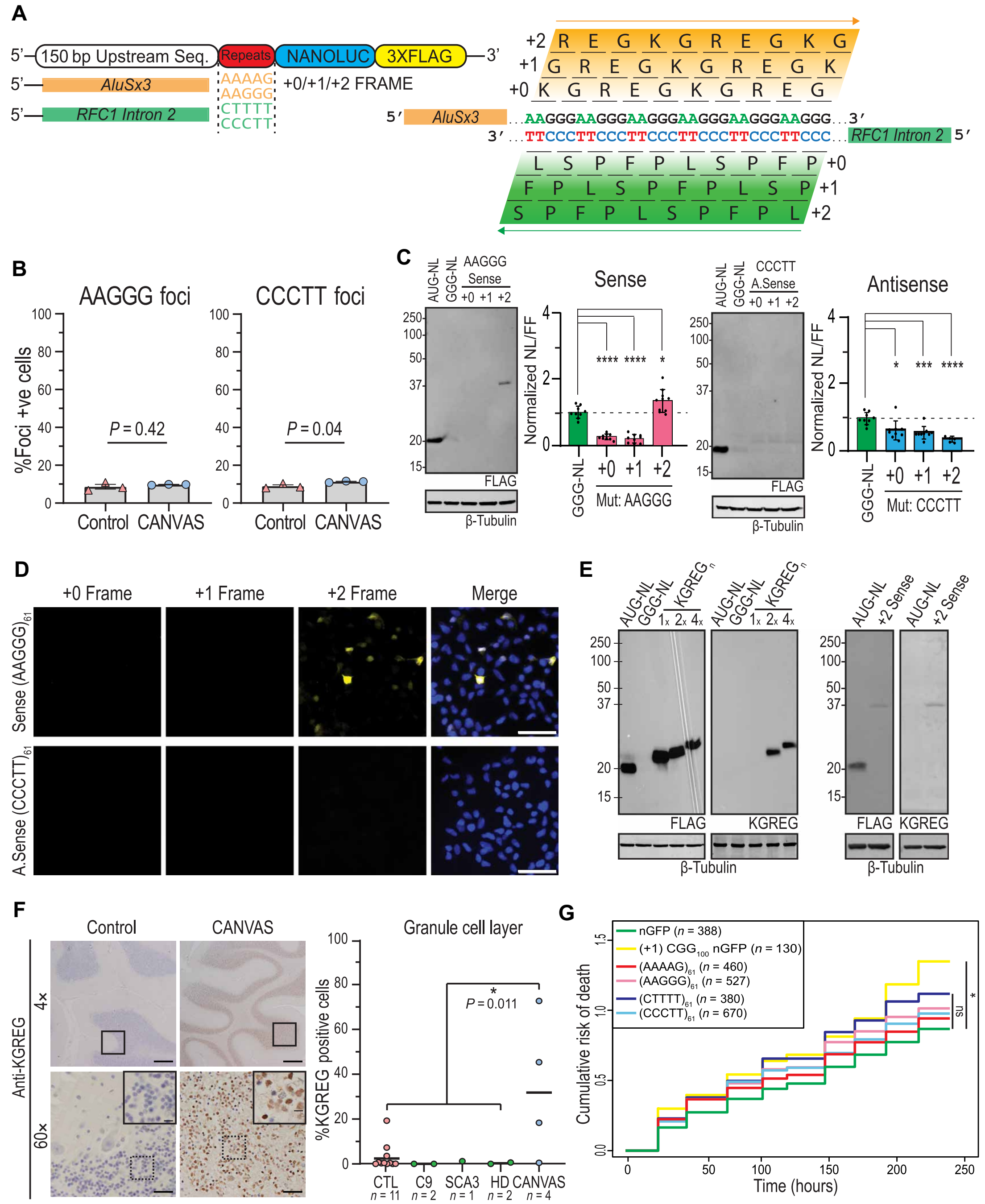

Fig. 2. Translated AAGGG repeat products are detected in brains of patients with CANVAS. (**A**) Schematic of potential peptide products from sense and antisense strand of the repeat expansion locus. (**B**) Quantification of foci positive neurons for control (n = 3) and CANVAS (n = 3) patient iPSC-derived neurons (1100 to 2000 cells per group per probe). n = 2 biological replicates from three independent patient-derived cell lines. Representative confocal images are shown in fig. S2B. (**C**) Immunoblot from HEK293 cells expressing plasmids encoding intronic sense or antisense AAGGG/CCCTT repeat reporters in the +0/+1/+2 reading frames (left) and Nano-luciferase expression assay quantification (right). n = 7 biological replicates. Data were analyzed by one-way ANOVA with Sidak's post hoc multiple comparison tests. (**D**) ICC of HEK293 cells transfected with plasmids encoding intronic sense or antisense AAGGG/CCCTT repeat reporters with C-terminal triple tags in the +0/+1/+2 reading frames. (**E**) Expression analysis of lysates from HEK293 cells transfected with control plasmid +2 Sense $(AAGGG)_{61}$ plasmid using anti-FLAG M2 (1:1000) and anti-KGREG (1:100) antibodies (left). (**F**) Left: IHC of control and *RFC1* expansion CANVAS patient postmortem cerebellar vermis tissue stained with sense anti-KGREG antibody (1:100, acid AR). Scale bars, 500 μm (4×), 50 μm (60×), and 20 μm (inset). Right: Rater blinded quantification of all 20 postmortem tissues (tissue images and quantification for each sample in fig. S4). (**G**) Cumulative hazard plot for rat cortical neurons expressing CGG100 (positive control) or CANVAS intronic expression plasmids containing 61 repeats of the indicated type over 10 days. Results from eight technical replicates/three biological replicates; n = numbers of cells assessed per condition. ns, not significant. *hazard ratio = 1.339, P = 0.025, Cox proportional hazards analysis.

the sense (AAGGG) strand +2 reading frame (Fig. 2C, left). In contrast, no products were detected from the antisense *RFC1* intron in all three reading frames (Fig. 2C, right), despite the presence of an AUG codon in the +0 reading frame. This was also observed by immunocytochemistry (ICC) in HEK293 cells, whereby only cells transfected with the +2 sense AAGGG reporter showed translation of any repeat-containing peptides (Fig. 2D).

If the AAGGG repeat were to be translated into a protein, it would generate the same pentapeptide repeat protein, polyKGREG, in all three potential reading frames. We therefore generated antibodies against a polyKGREG epitope. This antibody showed a high degree of specificity for two or more KGREG repeats, which are otherwise not predicted to occur in the human proteome, in transfected HEK293 cells by immunoblot staining (Fig. 2E, left) and strong colocalization with FLAG antibodies by ICC (fig. S3A). Using this antibody, we confirmed that the +2 sense AAGGG reporter product contained KGREG peptides (Fig. 2E, right). We next performed ICC using our KGREG and PFPSL antibodies in patient iNeurons. At 8 weeks of age, we did not see accumulation of these proteins by staining in CANVAS neurons compared to controls (fig. S3B).

To assess whether sense strand–derived KGREG peptide products accumulate in brains of patients with CANVAS, we performed immunohistochemistry on CANVAS (n = 4) as well as control [n = 16, including disease controls from cases of spinocerebellar ataxia type 3 (n = 1), C9orf72-associated FTD/ALS (n = 2), Huntington's disease (n = 2), and 11 nondisease controls] postmortem brain samples obtained from the University of Michigan Brain Bank, MassGeneral Brigham SCiN, Queen Square Brain Bank UCL, and the Netherlands Brain Bank (Fig. 2F, fig. S4, and table S3). KGREG staining was detected within cerebellar granule cells in three of four CANVAS cases, with only weak staining in the fourth case. In the controls, strong positive staining was observed in one control but was not reliably observed in any of the disease controls. The positive control case was screened for and did not have a repeat expansion in *RFC1*. While brains of patients with CANVAS showed marked Purkinje cell loss, no KGREG staining was observed in the Purkinje cells that remained (Fig. 2F and fig. S4). Of note, the CANVAS case with minimal staining for KGREG had relative preservation of Purkinje cells and was shown to carry a complex two-motif expansion on one allele $(AAAGG)_{610}(AAGGG)_{390}$ in trans with a second $(AAGGG)_{1170}$ expansion, on previous long-read DNA sequencing (*13*). As these assays did not reveal a perfect correlation with genotype, we reassessed our pathologic samples in a rater-blinded fashion using an established scoring criteria (*33*). These studies showed significantly more staining in CANVAS cases compared to controls, and significantly more cases with at least 10% of their granule cells positive for staining. These data suggest that pentapeptide KGREG repeat proteins may be produced from AAGGG repeats in patients with CANVAS in a cell type–specific manner. Of note, we did not see specific staining for the antisense PFPSL in CANVAS brains compared to controls (fig. S5).

Ectopic expression of either CGG or CAG repeats in rodent neurons is sufficient to elicit toxicity, which can serve as a proxy for gain of function–associated neurodegeneration (*34*–*38*). To assess whether ectopic AAGGG or CCCTT repeat expression might elicit neurotoxicity, we used automated longitudinal fluorescence microscopy and survival analysis (*39*–*41*) of neurons expressing the intronic WT or mutant sense or antisense reporter constructs in primary rat cortical neurons. Compared to expression of green fluorescent protein (GFP) alone, expression of these repeats failed to elicit significant toxicity over a 10-day period ($AAAAG_{61}$: P = 0.75, $AAGGG_{61}$: P = 0.45, $CTTTT_{61}$: P = 0.11, and $CCCTT_{61}$: P = 0.65) (Fig. 2G). CGG repeat expression studies performed in parallel served as a positive control with significant toxicity compared to GFP. Together, our data suggest that AAGGG repeats can form RNA foci and be translated into pentapeptide repeat proteins. However, these products do not accumulate at detectable levels in iNeurons derived from patients with CANVAS, and expression of 61 repeats is insufficient to elicit neurodegeneration in a rodent neuronal model system. Additional assays and in vivo studies will be needed to discern whether the repeat as transcribed RNA or as a translated protein expressed outside of its native locus contributes meaningfully to disease pathogenesis.

Splicing of RFC1 is normal in multiple CANVAS patient–derived cell types

Intronic repeat expansions can interfere with pre-mRNA splicing by inducing intron retention within the mature transcript (*42*, *43*), stabilizing circular intronic lariat species (*44*), or inducing differential exon usage during splicing (*45*–*47*). Intronic *Alu* elements also influence alternative splicing through inclusion of *Alu*-containing exons, with 85% of *Alu* containing exons deriving from antisense *Alu* elements (*48*, *49*). To assess whether AAGGG/CCCTT repeat expansions led to retention of *RFC1* intron 2, we performed endpoint reverse transcription polymerase chain reaction (RT-PCR) with primers that amplify the exon 2–exon 3 or the exon 2–intron 2 junctions (Fig. 3A and fig. S6A). There were no differences between cases and controls in terms of intron retention or aberrant intron 2 splicing of *RFC1* in patient fibroblasts, iPSC-derived neurons, or cortical and cerebellar regions of postmortem brain (Fig. 3A).

To investigate whether AAGGG/CCCTT repeat expansions trigger *RFC1* intron 2 stabilization as a circular lariat species, as may occur in *C9orf72* FTD/ALS (*44*), we used whole-transcriptome RNA short read sequencing to map *RFC1* mRNA splicing isoforms and identify the presence of intronic back-spliced reads indicative of circular mRNA species (circRNAs) arising from circular intronic lariats (*50*). Total rRNA-depleted RNA was extracted from 10-week-aged CANVAS patient and control iPSC–derived neurons and paired-end reads were processed and analyzed using packages described in Materials and Methods. A known list of circRNA species (*51*–*55*) were detected at comparable levels in CANVAS and control samples (Fig. 3B); however, no back-spliced reads were identified to map to *RFC1* intron 2 or across the *RFC1* transcript, indicating a lack of detectable circular RNA species deriving from the *RFC1* locus (Fig. 3B).

To assess whether AAGGG/CCCTT repeat expansions affect *RFC1* mRNA isoforms, we used DEXSeq (*56*) to assess the normalized differential exon usage of mature spliced *RFC1* transcripts in CANVAS patient and control iPSC–derived neurons (fig. S6B). No changes in *RFC1* exon usage were observed within the N-terminal region flanking the repeat expansion, or across the entire *RFC1* transcript. Similarly, Sashimi plots of *RFC1* splicing demonstrate similar splicing and alternative exon usage in CANVAS patient iPSC–derived neurons compared to controls without evidence for alternative noncanonical exon usage for *RFC1* in normal or disease states (fig. S6C).

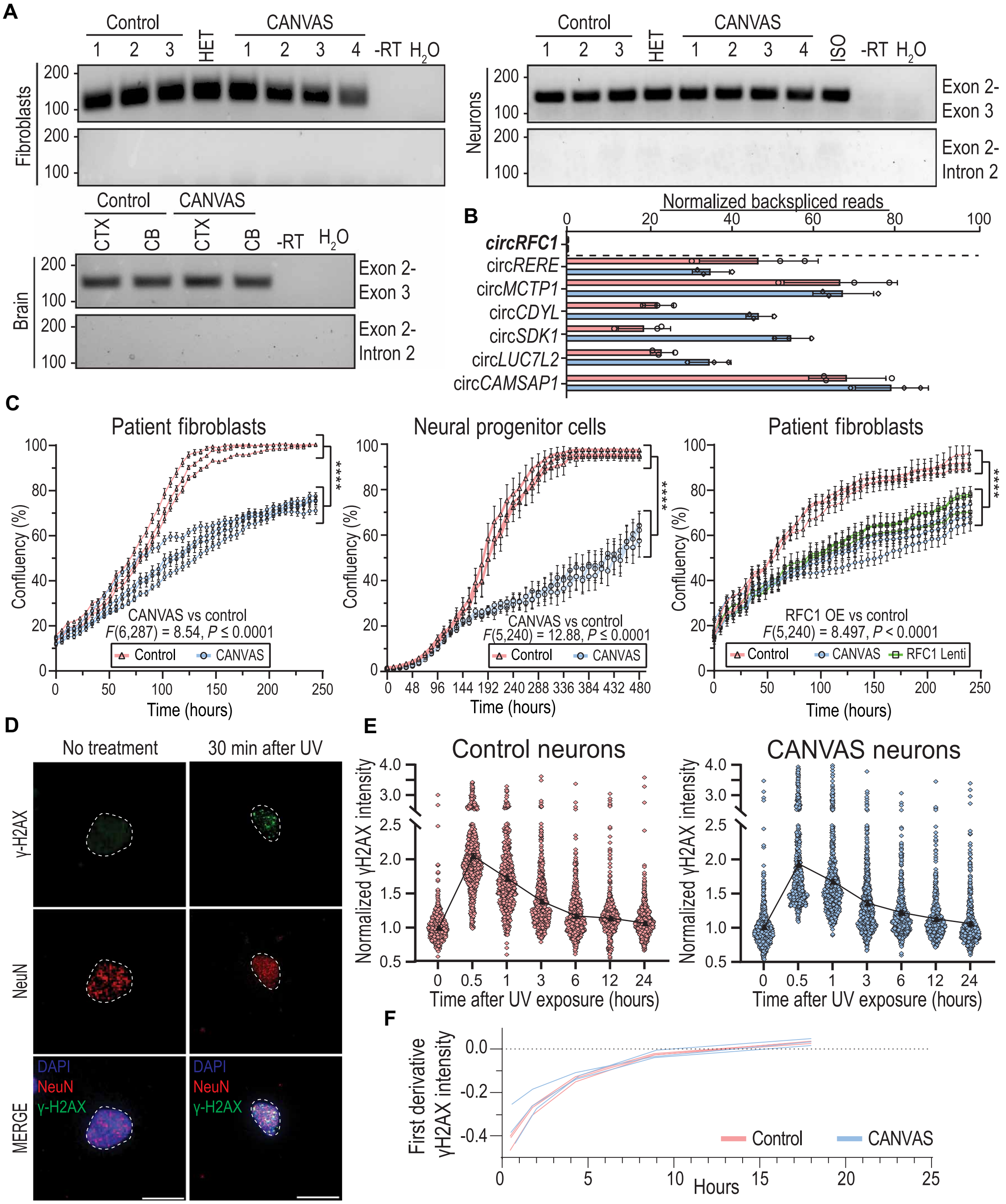

Fig. 3. Canonical functions and expression of RFC1 are normal in cells derived from patients with CANVAS. (**A**) Endpoint RT-PCR utilizing primer sets spanning *RFC1* exon 2–exon 3 or exon 2–intron 2 in CANVAS fibroblasts (top, left), iPSC-derived neurons (top, right), and CANVAS postmortem brain (bottom, left). (**B**) Quantification of normalized circular back-spliced read counts for *RFC1* and other known circRNA species in CANVAS patient iPSC–derived neurons by paired-end RNA-seq analysis. (**C**) Ten-day time-course analysis of the rate of cellular division and proliferation in CANVAS ($n = 4$) and control ($n = 3$) fibroblast lines (left, $F_{6,287} = 8.54$, $P < 0.0001$), CANVAS ($n = 3$) and control ($n = 3$) NPC lines (center, $F_{5,240} = 12.88$, $P < 0.0001$), and CANVAS fibroblast lines ($n = 3$) mock-treated or treated with RFC1 overexpression lentivirus (right, $F_{5,240} = 2.358$, $P = 0.245$). $n = 3$ biological replicates from three to four independent patient cell lines. Data were analyzed by one-way ANOVA with Sidak's post hoc multiple comparison tests. (**D** and **E**) Analysis of recovery after discrete UV exposure and DNA damage in CANVAS patient iPSC–derived neurons. (D) Representative images of γ-H2AX staining of iPSC-derived neurons before and after 60 mJ/cm^2 UV exposure (scale bar, 10 μm). (E) Quantification of mean γ-H2AX staining in CANVAS patient ($n = 3$) and control ($n = 3$) iPSC–derived NeuN+ neuronal nuclei over a 24-hour period after 60 mJ/cm^2 UV exposure ($n = 16,569$ NeuN+ nuclei total). Data were analyzed by one-way ANOVA with post hoc multiple comparison tests. (**F**) First-derivative DNA damage recovery rate curves for CANVAS ($n = 3$) and control ($n = 3$) patient iPSC–derived neurons. Error = SD.

Canonical functions and expression of RFC1 are normal in cells derived from patients with CANVAS

Prior studies in fibroblasts, lymphoblasts, frontal cortex and cerebellar vermis of patients with CANVAS demonstrated no differences in *RFC1* mRNA when compared to controls (*8*). Consistent with prior studies, we observed no changes in *RFC1* steady-state mRNA abundance or RFC1 protein expression between control ($n = 3$), CANVAS ($n = 4$), and heterozygous carrier patient fibroblasts ($n = 1$) (fig. S7A). Similarly, we observed no changes in *RFC1* mRNA or RFC1 protein levels between 6-week-old control ($n = 3$) and CANVAS ($n = 3$) patient iPSC–derived neurons (fig. S7B). Analysis in limited postmortem frontal cortex and cerebellar brain samples showed no change in *RFC1* mRNA or RFC1 protein expression levels (fig. S7C).

RFC1 plays critical roles in both DNA replication and DNA damage repair as a subunit of the DNA clamp-loader complex (*57–59*). To assess whether AAGGG/CCCTT repeat expansions might interfere with RFC1's DNA replication functions, we first investigated the rate of cellular proliferation in age- and passage-matched CANVAS ($n = 4$) and control ($n = 3$) patient–derived fibroblasts (Fig. 3C, left). We observed a significant decrease in cellular proliferation rate for CANVAS fibroblasts when compared to controls ($F_{6,287} = 8.54$, $P < 0.0001$). We confirmed this phenotype in CANVAS neural progenitor cells ($F_{5,240} = 12.88$, $P < 0.0001$; Fig. 3C, center). To determine whether this CANVAS proliferation phenotype was dependent on RFC1 expression or function, control-derived fibroblasts ($n = 3$ per group) were transduced with lentiviral particles encoding short hairpin RNAs (shRNAs) targeting *RFC1* exon 4 and exon 15, or a control shRNA, and were assessed for proliferation rate over a 10-day period as before (fig. S7D). *RFC1* knockdown reduced RFC1 protein to nearly undetectable levels (fig. S8). Consistent with its known roles in DNA replication, RFC1 knockdown in control fibroblasts resulted in an initial lag in cellular proliferation compared to control shRNA-treated fibroblasts (120 hours, $P = 0.001$), which recovered to control levels after 200 hours ($F_{5,240} = 1.314$, $P = 0.131$), perhaps due to preferential growth of cells no longer expressing the shRNA. In contrast, untreated fibroblasts from patients with CANVAS continued to show a slower proliferation rate throughout the analysis ($F_{5,240} = 4.417$, $P = 0.0007$). To assess whether reprovision of full-length WT RFC1 was sufficient to overcome the CANVAS phenotype, we overexpressed full-length RFC1 in CANVAS patient–derived fibroblasts (Fig. 3C, right). CANVAS patient–derived fibroblasts overexpressing RFC1 retained a markedly slowed proliferation rate compared to control fibroblasts ($F_{5,240} = 8.497$, $P < 0.0001$), with no significant improvement over the proliferation rate of CANVAS patient–derived fibroblasts treated with a control lentivirus ($F_{5,240} = 2.358$, $P = 0.245$). These results suggest that AAGGG/CCCTT repeat expansions affect cell proliferation independent of RFC1 protein levels.

CANVAS is predominantly a neuronopathy, and as neurons are post-mitotic, we investigated whether DNA damage repair was dysfunctional in CANVAS patient iPSC–derived neurons. To do this, we analyzed expression of the DNA damage marker γ-H2AX in 8-week-old control and CANVAS iPSC–derived neurons where basal levels of DNA damage accumulation was comparable between control and CANVAS iPSC–derived neurons (fig. S9A). To test for an altered response to DNA damage, CANVAS patient and control iPSC–derived neurons were exposed to ultraviolet (UV) irradiation (Fig. 3, D and E). As expected, γ-H2AX levels increased after exposure to 0 to 120 mJ/cm^2 UV irradiation in control iPSC–derived neurons (fig. S9B). Control ($n = 3$) and CANVAS patient ($n = 3$) iPSC–derived neurons showed a consistent ~2-fold increase in γ-H2AX reactivity within 30 min of 60 mJ/cm^2 UV irradiation, followed by a slow but consistent decline in γ-H2AX reactivity in all experimental conditions, with return to baseline γ-H2AX reactivity within 6 to 12 hours after irradiation. First-derivative analysis of normalized γ-H2AX indicated no differences in the rate of γ-H2AX recovery after UV induction between CANVAS and control neurons (Fig. 3, E and F, and fig. S9, C and D), suggesting that repair of DNA damage induced by UV irradiation is not affected in CANVAS neurons.

Synaptic genes are down-regulated in CANVAS patient–derived neurons

To assess for differences between CANVAS and control iPSC–derived neurons that might provide some insights into disease pathogenesis, we next conducted paired-end sequencing of total RNA extracted from 10-week-old CANVAS patient ($n = 4$) and control ($n = 3$) iPSC–derived neurons to identify potential dysregulated pathways or genes in CANVAS. An average of ~20 million to 30 million reads were obtained per sample, with technical replicates generated for each line from different differentiations. After trimming, genome alignment, and filtering for low transcript reads (see Materials and Methods), we observed that 5.9% (1313) of detected genes were up-regulated and 11.7% (2630) were down-regulated in CANVAS patient iPSC–derived neurons compared to control neurons (Fig. 4A). *RFC1* transcripts showed a modest but significant increase in CANVAS iNeurons that was below the prespecified fold-change threshold implemented (Fig. 4A), with no RFC1-associated GO terms identified within overrepresented cellular pathways of these dysregulated transcripts in CANVAS (Fig. 4B). Principal components analysis (PCA) clustering of neuronal samples based on gene expression patterns demonstrated discrete and distinct clustering of CANVAS neuronal samples as separate from controls (Fig. 4C) with 96% of observable variance seen across principal component 1 (PC1), indicating that variability between control and CANVAS gene expression profiles is the dominant source of variation between iNeuron lines. Heatmap analysis of the top dysregulated transcripts detected in CANVAS patient iPSC–derived neurons shows a strong preference for reduced expression of transcripts (Fig. 4D). Gene Ontology (GO) analysis indicated a significant overrepresentation of neuronal signaling processes including synaptic signaling and processes that regulate synaptic signaling in CANVAS versus control (Fig. 4B and fig. S10), with many of these transcripts expressed in neuronal processes and involved in signaling, channel, or transporter activity. Normalized transcript counts for select dysregulated synaptic genes indicate that this reduction in synaptic gene expression is highly significant and encompasses both pre- and postsynaptic genes, as well as genes involved in synaptic organization and signal transduction (Fig. 4E). A full analysis of dysregulated pathways is shown in fig. S10.

Protein expression analyses of select synaptic genes significantly down-regulated in transcriptomic analyses in Fig. 4E found similar down-regulation at the protein level. Synaptophysin, CHL1, GAP43, and CAMKIIB all showed significant reductions in expression at both the transcript and protein level in CANVAS patient iPSC–derived neurons compared to control (Figs. 4E and 5, A to D). Assessment of these same proteins by immunoblot from the cerebellum and

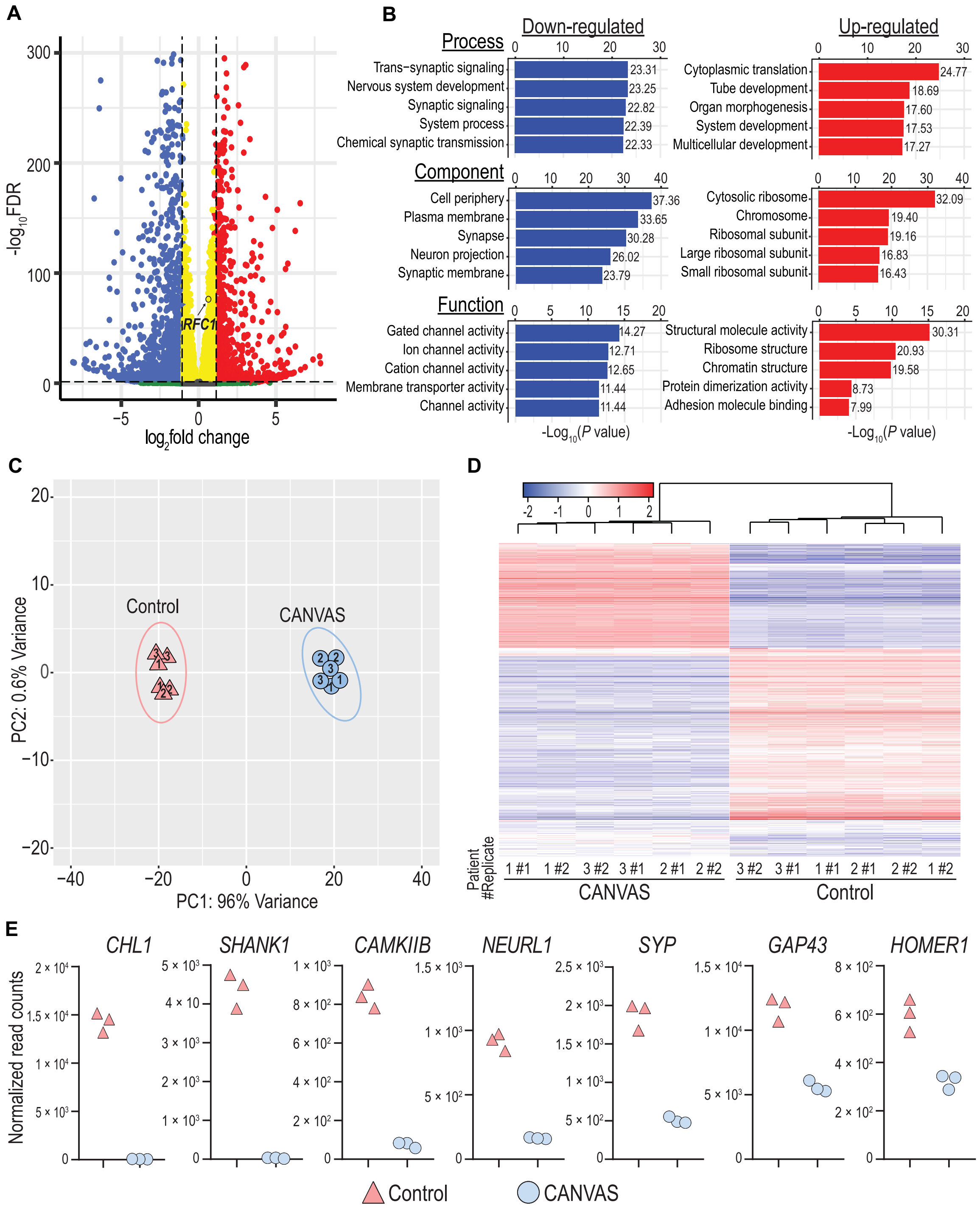

Fig. 4. Synaptic genes are down-regulated in CANVAS neurons. (**A**) Volcano plot of differentially expressed genes in CANVAS patient versus control iPSC–derived neurons, blue = significantly down-regulated, red = significantly up-regulated, *RFC1* labeled. (**B**) Gene Ontology (GO) pathway analysis of the top five up-/down-regulated biological process, cellular component, and molecular function in CANVAS ($n = 3$) versus control ($n = 3$) patient iPSC–derived neurons. (**C**) Principal components analysis (PCA) of CANVAS ($n = 3$) versus control ($n = 3$) patient iPSC–derived neurons, patient number indicated within shapes identify technical replicates. (**D**) Heatmap of normalized expression for the top 1000 genes differentially expressed in CANVAS patient versus control iPSC–derived neurons. (**E**) Normalized gene counts for the top seven down-regulated synaptic-associated genes in CANVAS patient versus control iPSC–derived neurons.

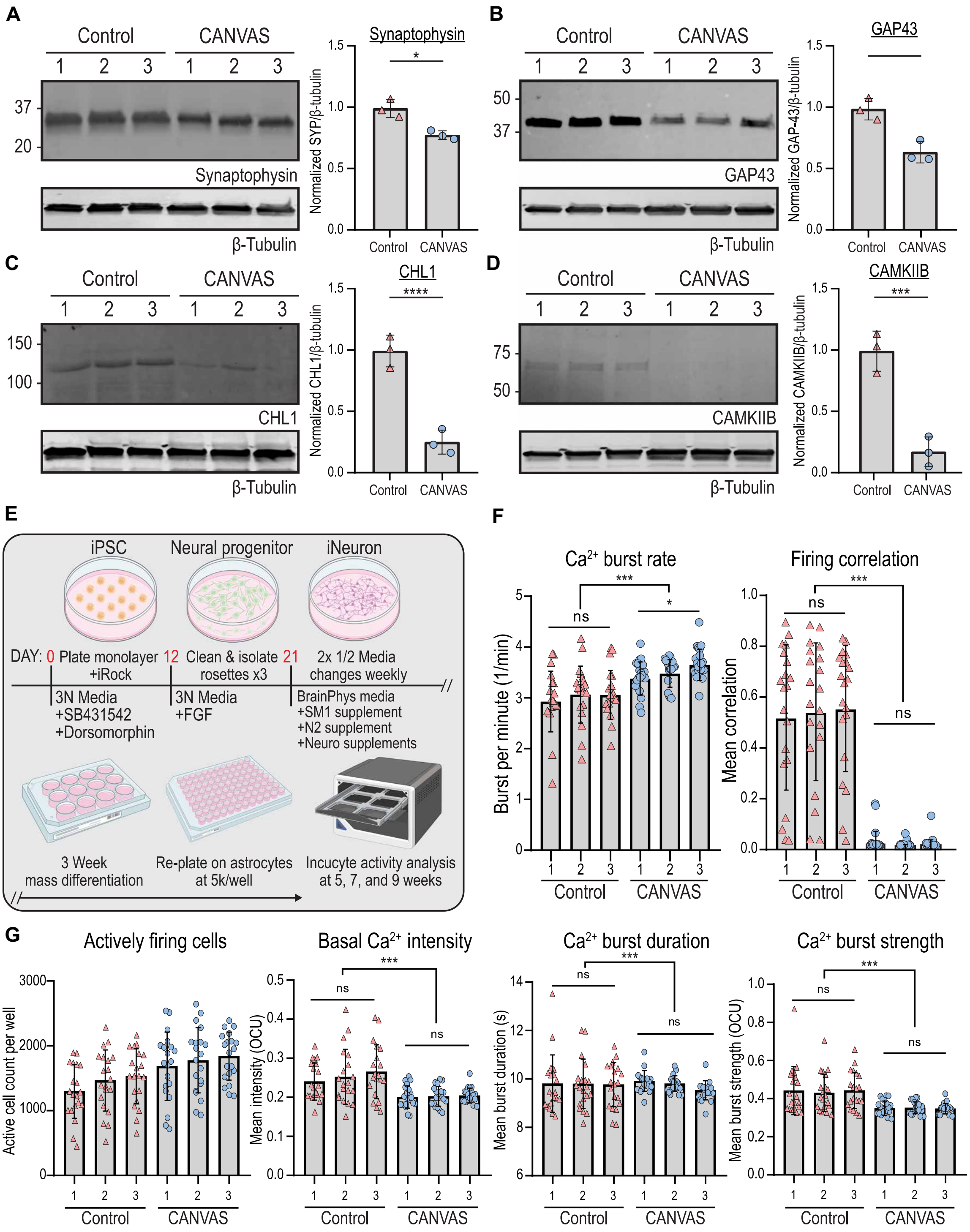

Fig. 5. CANVAS patient–derived neurons exhibit synaptic dysfunction and reduced connectivity. (**A** to **D**) Protein expression (left) and normalized quantification (right) of selected synaptic genes identified as down-regulated in CANVAS patient iPSC–derived neurons by transcriptomic analysis: (A) synaptophysin, (B) GAP43, (C) CHL1, and (D) CAMKIIB. $n = 3$ per group. Data were analyzed by one-way ANOVA with Sidak's post hoc multiple comparison tests. (**E**) Schematic outlining experimental workflow for generating patient iPSC–derived neurons for calcium imaging analysis. (**F**) Analysis of Ca^{2+} imaging metrics for control ($n = 3$) and CANVAS ($n = 3$) patient iPSC–derived neurons at 9 weeks after differentiation. Burst rate ($F_{5,114} = 8.268$, $P < 0.0001$) and firing correlation ($F_{5,114} = 45.62$, $P < 0.0001$). (**G**) Analysis of Ca^{2+} imaging metrics for control ($n = 3$) and CANVAS ($n = 3$) patient iPSC–derived neurons. Basal intensity ($F_{5,114} = 7.075$, $P < 0.0001$), burst duration ($F_{5,114} = 0.5371$, $P = 0.745$), and burst strength ($F_{5,114} = 7.573$, $P < 0.0001$). Each data point represents the mean of ~1000 to 3000 active cells per well. Data were analyzed by one-way ANOVA with Sidak's post hoc multiple comparison tests. Error = SD.

cortex of patients with CANVAS shows similar qualitative patterns of reduced expression, but an insufficient number of cases with cortical tissue available precluded quantitation (fig. S11A).

Spontaneous synaptic activity is impaired in CANVAS patient–derived neurons

Given the dysregulation of synapse-associated transcripts and proteins we observed in CANVAS patient iPSC–derived neurons, we investigated whether these alterations in gene expression correlated with observable phenotypic differences in synaptic activity. To accomplish this, we measured spontaneous synaptic activity in control ($n = 3$) and CANVAS ($n = 3$) glutamatergic forebrain neurons with the Incucyte Neuroburst Orange fluorescent calcium indicator and Incucyte S3 automated imaging system. Patient-derived neural progenitor cells were differentiated and re-plated at uniform density on an astrocyte feeder layer as described in Materials and Methods (Fig. 5E). Neurons were analyzed for active cell number, basal calcium intensity, burst rate, burst strength, burst duration, and network correlation between 3 and 11 weeks after differentiation (Fig. 5, F and G), and wells with at least 500 active cells were included in the analysis. No morphological abnormalities were noted between control and CANVAS iPSC–derived neurons. Both control and CANVAS iPSC–derived neurons consistently had between 1000 and 2000 active cells per well, with minor but statistically significant reductions observed for basal calcium intensity, burst duration, and burst strength in CANVAS neurons (Fig. 5G). Notably, while control iPSC–derived neurons formed synchronized networks with 50 to 70% network correlation by 5 weeks after differentiation (Figs. 5F and 6E), CANVAS patient iPSC–derived neurons remained devoid of detectable synchronous firing even between 7 and 11 weeks after differentiation, with single cells showing independent activity at a 17% increase in firing rate relative to control (Fig. 5F and movies S1 to S3). These between-genotype findings were robust to corrections for differences in neuronal density and active cell counts ($F_{5,114} = 59.89$, $P < 0.0001$) (fig. S11B).

Synaptic gene expression is restored upon heterozygous correction of RFC1 repeat expansion

To determine the impact of the AAGGG/CCCTT repeat expansion in CANVAS patient iPSC–derived neurons, we generated an isogenic line with a monoallelic deletion of the repeat and compared its gene expression to both CANVAS and control iPSC–derived neuronal lines. Surprisingly, the heterozygous isogenic line substantially corrected across PC1 that separates CANVAS from control neuronal samples, while variance along PC2 was increased substantially, indicating partial correction of some additional CANVAS-associated variance to control and the emergence of a new source of variance after heterozygous deletion of the AAGGG/CCCTT repeat expansion (Fig. 6A and fig. S12, A and B). This large-scale correction of the CANVAS-associated transcriptomic signature is also visible by heatmap analysis illustrating that the heterozygous isogenic patient iPSC–derived neurons exhibit a global gene expression pattern more similar to that of control than CANVAS (fig. S12C).

In total, 64% of the CANVAS up-regulated genes and 70% of the CANVAS down-regulated genes exhibited at least a 50% correction in expression upon monoallelic deletion of the AAGGG/CCCTT repeat expansion in the CANVAS patient 1 line (Fig. 6B), and 80.5% of these genes (2027:491) were no longer significantly different compared to controls (Fig. 6C, green). The GO terms of these gene expression corrected genes correlate with many of the top dysregulated pathways in CANVAS, including multiple synapse-associated processes and functions (Fig. 6C, right, and fig. S12D). Within these pathways, the key synaptic genes found to be most down-regulated in CANVAS (Fig. 4E) show significant or almost complete restoration in the isogenic neurons compared to control (fig. S12E), with *CAMKIIB, GAP43, HOMER1, NEURL1*, and *SYP* showing the greatest restoration in the isogenic line.

To assess whether the correction we observed in our isogenic line compared to its parent line and its differences from the control line could be explained by the presence of a single repeat allele, we assessed an additional iPSC line derived from a heterozygous asymptomatic carrier daughter of patient 2. This line has a single AAGGG repeat allele of size 1314 repeats and a short AAAAG allele (Fig. 1E). Transcriptomic analysis of this line revealed a pattern very similar to the isogenic control line and more similar to the nonisogenic control lines than to the CANVAS lines (fig. S12, B and C). This heterozygous line clusters with the isogenic control line as a separate group through PC analysis—perhaps reflecting the impact of a single expanded AAGGG repeat allele on gene expression.

Dysregulated synaptic activity is rescued by heterozygous correction of RFC1 repeat expansion

We next analyzed spontaneous synaptic activity upon heterozygous isogenic correction in comparison to both control and CANVAS patient iPSC–derived neurons (Fig. 6D). Heterozygous isogenic neurons exhibited modest but significant improvements in basal calcium intensity and burst duration compared to CANVAS neurons (fig. S11C), as well as large and significant improvements in neuronal burst rate synchronized firing (Fig. 6, D and E), with an average network correlation of 42% compared to 70% in controls. While these metrics do not indicate complete recovery of deficits observed in CANVAS versus control, the significant trend toward recovery in all metrics in conjunction with the observed correction in gene expression upon deletion of a single expanded allele in CANVAS neurons suggests a repeat-dependent mechanism of CANVAS pathogenesis. Similarly, the heterozygous control case exhibits an electrophysiologic profile that closely resembles that of both the isogenic control and the clinical control lines and is notably and significantly distinct/different from the CANVAS lines (Fig. 6D and fig. S11C).

RFC1 reduction does not mimic CANVAS-associated transcriptomic or functional deficits

If loss of RFC1 protein function is central to CANVAS pathogenesis, then targeted reduction of RFC1 should recapitulate defects observed in neurons from patients with CANVAS. To test this hypothesis, we conducted transcriptomic and functional neuronal analyses of control neurons after sustained (~10 weeks) knockdown of *RFC1* in comparison to CANVAS neurons. Treatment with RFC1 shRNA lentivirus led to undetectable levels of RFC1 protein (Fig. 7A and fig. S8B), and normalized *RFC1* gene counts across experimental conditions showed efficient knockdown of *RFC1* transcripts with ~63% reduction in comparison to control neurons and ~75% reduction in comparison to CANVAS neurons (Fig. 7B, left). Compared to control neurons expressing a nontargeting shRNA, *RFC1* knockdown led to statistically up-regulated expression of only 0.5% (235) of detected genes and statistically down-regulated expression of only 0.23% (103) of detected genes in iPSC-derived neurons. PCA

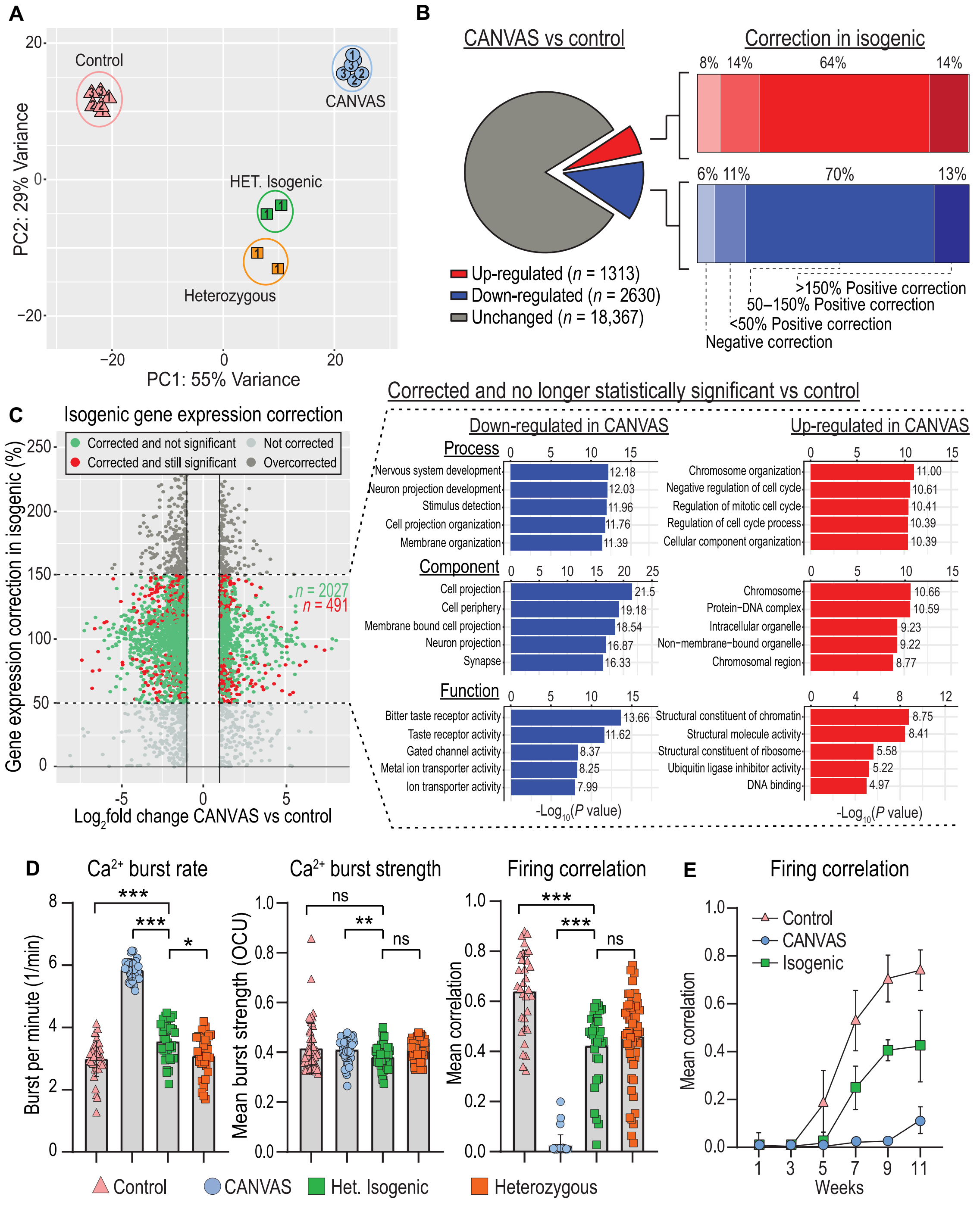

Fig. 6. Heterozygous isogenic correction of CANVAS neurons corrects transcriptomic and synaptic functional deficits. (**A**) PCA of CANVAS ($n = 3$), control ($n = 3$), heterozygous ($n = 1$), and CANVAS heterozygous isogenic ($n = 1$) patient iPSC–derived neurons. Patient number indicated within shapes identify technical replicates. (**B**) Schematic illustrating the total number of genes dysregulated (up and down) in CANVAS versus control (left), with the percentage of these up- or down-dysregulated genes that show negative correction, partial correction, or full correction of expression upon heterozygous isogenic correction of CANVAS patient iPSC–derived neuron line. (**C**) Scatter plot of $Log_2FoldChange$ (CANVAS versus control) versus gene expression correction per gene in the heterozygous isogenic patient iPSC–derived neurons (left), and GO pathway analysis of the top five up-/down-regulated biological process, cellular component, and molecular function for the genes that show 50 to 150% gene expression in isogenic correction versus CANVAS and are nonstatistically significant in isogenic versus control conditions. (**D**) Analysis of Ca^{2+} imaging metrics for control ($n = 3$), CANVAS ($n = 3$), heterozygous ($n = 1$), and heterozygous isogenic ($n = 1$) patient iPSC–derived neurons. Burst rate ($F_{2,165} = 279.4$, $P < 0.0001$), burst strength ($F_{2,165} = 4.034$, $P = 0.019$), and firing correlation ($F_{2,165} = 185.9$, $P < 0.0001$). Each data point represents the mean of ~1000 to 3000 active cells per well (fig. S8). Data were analyzed by one-way ANOVA with Sidak's post hoc multiple comparison tests. (**E**) Mean firing correlation of control ($n = 3$), CANVAS ($n = 3$), and heterozygous isogenic ($n = 1$) patient iPSC–derived neurons across 10 weeks of differentiation from week 1 to week 11. Error = SD.

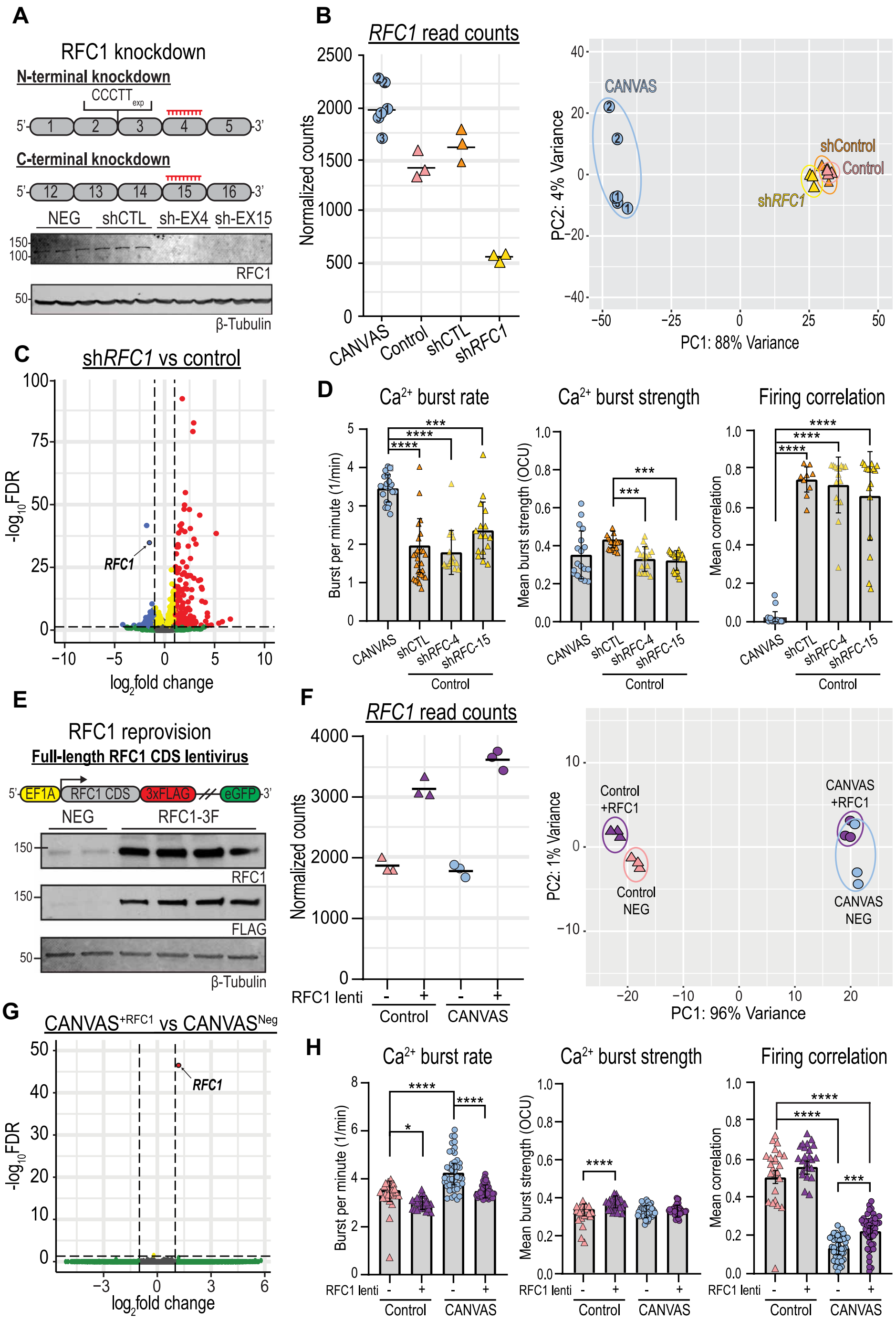

Fig. 7. Altering RFC1 expression neither recapitulates nor corrects CANVAS patient neuron dysfunction. (**A**) Schematic of *RFC1* knockdown using *RFC1* N or C terminus targeting shRNA lentiviruses (top) and RFC1 expression after knockdown in control iPSC–derived neurons (bottom). (**B**) Normalized read counts for *RFC1* transcripts (left) and PCA (right) in CANVAS ($n = 3$), control ($n = 3$), control mock-treated ($n = 3$), and control sh*RFC1*-treated ($n = 3$) patient iPSC–derived neurons. (**C**) Volcano plot of *RFC1* knockdown versus control, *RFC1* labeled. (**D**) Ca^{2+} imaging metrics of CANVAS ($n = 3$) and control ($n = 3$) patient iPSC–derived neurons treated with shControl or sh*RFC1* lentiviruses. Burst rate ($F_{3,78} = 29.6$, $P < 0.0001$), burst strength ($F_{3,78} = 8.265$, $P < 0.0001$), and firing correlation ($F_{3,78} = 100.6$, $P < 0.0001$). (**E**) Schematic of *RFC1* overexpression in CANVAS patient iPSC–derived neurons (top) and analysis of RFC1 expression in patient iPSC–derived neurons upon lentiviral transduction (bottom). (**F**) Normalized read counts for *RFC1* transcripts (left) and PCA of CANVAS and control iPSC–derived neurons transduced either full-length *RFC1* CDS or control lentivirus ($n = 3$ per group) (right). (**G**) Volcano plot of CANVAS patient–derived neurons transduced with either full-length *RFC1* CDS or control lentivirus ($n = 3$ per group), *RFC1* labeled. (**H**) Ca^{2+} imaging metrics of control ($n = 3$) and CANVAS ($n = 3$) patient iPSC–derived neurons treated with control or *RFC1*-overexpression lentivirus. Burst rate ($F_{3,135} = 31.01$, $P < 0.0001$), burst strength ($F_{3,135} = 16.74$, $P < 0.0001$), and firing correlation ($F_{3,135} = 147.3$, $P < 0.0001$). Firing correlation two-way ANOVA treatment versus genotype: $F_{1,135} = 41.25$, $P < 0.0001$ and $F_{1,135} = 36.64$, $P < 0.0001$, respectively. Each data point represents the mean of ~1000 to 3000 active cells per well (fig. S5). Data were analyzed by one-way ANOVA with Sidak's post hoc multiple comparison tests. Patient numbers indicated identify technical replicates. Error = SD.

clustering showed no overlap between *RFC1* shRNA-treated iPSC-derived neurons and CANVAS neurons and instead showed tight clustering with nontargeting shRNA-expressing controls (Fig. 7B, right, and fig. S13, A and B), indicating that *RFC1* knockdown in control neurons does not induce CANVAS-like transcriptomic alterations. Furthermore, a volcano plot of *RFC1* knockdown versus control neurons (Fig. 7C) indicates less dysregulation in gene expression when compared to CANVAS versus control (Fig. 4A), with a preference for increased expression for a small number of transcripts and a reduction in *RFC1* (circled). Similarly, none of the key synaptic genes found to be highly down-regulated in CANVAS (Fig. 4E) showed significant dysregulation upon *RFC1* knockdown (fig. S13C).

Gene set enrichment analysis (GSEA) of ranked genes from *RFC1* knockdown showed an enrichment for annotated RFC1-associated functions (fig. S14). GO analysis for the most overrepresented cellular pathways within the transcripts dysregulated upon RFC1 knockdown, meanwhile, found that immune and developmental cellular processes were most significantly dysregulated (fig. S13D). There was no enrichment in synaptic-associated processes, in contrast to the significantly dysregulated pathways identified in CANVAS neurons versus control (Fig. 4B and figs. S10 and S13D).

To assess the impact of *RFC1* knockdown on neuronal function, we again used calcium imaging. Compared to control neurons, *RFC1* knockdown elicited no differences in basal calcium intensity, burst duration (fig. S11D), neuronal burst rate, or network firing correlation (Fig. 7D) and the *RFC1* knockdown neurons remained highly significantly different from CANVAS neurons in these four metrics. The only metric that showed any similarities with the findings in CANVAS neurons was burst strength, where knockdown of *RFC1* induced a 24% reduction (Fig. 7D, center, $P < 0.0001$), comparable to the levels seen in CANVAS neurons (CANVAS-shRNA1: $P = 0.915$, CANVAS-shRNA2: $P = 0.786$).

Reprovision of RFC1 does not correct CANVAS-associated transcriptomic or functional deficits

To determine whether RFC1 reprovision can correct phenotypic deficits observed in neurons from patients with CANVAS, we conducted transcriptomic and functional neuronal analyses of control and CANVAS iPSC–derived neurons after sustained (~10 weeks) expression of either GFP lentivirus or a full-length RFC1-encoding lentivirus with a C-terminal 3× FLAG tag and separate GFP reporter (Fig. 7E and fig. S8C). Transduction of this lentivirus significantly boosted RFC1 expression in patient iPSC–derived neurons (Fig. 7E), with a ~50 to 75% increase in detected *RFC1* transcripts compared to neurons treated with the GFP control lentivirus only (Fig. 7F, left). PCA clustering showed no overlap of RFC1 overexpression CANVAS neurons with control neurons and instead showed tight clustering between genotypes independent of treatment condition (Fig. 7F, right), indicating that *RFC1* overexpression fails to correct CANVAS neuronal transcriptomic alterations. Furthermore, a volcano plot of differential gene expression in CANVAS neurons overexpressing RFC1 compared to those expressing a control GFP lentivirus shows that sustained RFC1 reprovision effectively induces no transcriptomic changes in CANVAS neurons, with only *RFC1* found to be differentially expressed between these conditions (Fig. 7G). Similarly, comparison of RFC1-overexpressing CANVAS neurons with control neurons (fig. S15A) exhibited identical differential gene expression patterns as was observed in Fig. 4A. Overexpression of RFC1 in control neurons also triggered minimal changes in global gene expression (fig. S15B), and heatmap profiles of global gene expression across all conditions indicate that overexpression of RFC1 does not correct the transcriptomic deficits observed in CANVAS, and elicits negligible effects within control neurons (fig. S15C).

When analyzing the functional phenotypes observed for CANVAS neurons, overexpression of RFC1 elicited no differences for metrics that exhibited only minor or no dysregulation in CANVAS compared to controls (fig. S11E). Reprovision of RFC1 did reduce the abnormal firing burst rate in CANVAS neurons by 17.6%, which effectively corrected the difference between CANVAS and control neurons. However, RFC1 overexpression also induced a 13.9% reduction in burst rate within control neurons, suggesting that this effect is not specific to CANVAS neurons (Fig. 7H, left). Two-way analysis of variance (ANOVA) indicates that the effect sizes are comparable and that the CANVAS versus control genotype is not a driver of the effect (treatment: $F_{1,135} = 41.25$, $P < 0.0001$; genotype: $F_{1,135} = 36.64$, $P < 0.0001$). Similarly, RFC1 overexpression increased the firing correlation observed for CANVAS neurons (0.13 to 0.22, $P = 0.001$). However, this correction was modest, and these CANVAS neurons remained significantly less correlated than control neurons ($P < 0.0001$, Fig. 7H, right).

DISCUSSION

With an estimated carrier frequency upward of ~6% (*8*, *15*), nonreference AAGGG/CCCTT repeat expansions in *RFC1* are potentially notable contributors to neurologic disease, including both ataxia and sensory neuronopathy. Here, we used CANVAS patient–derived neurons, patient cells, and tissues to directly assess how these repeats elicit toxicity. Our findings suggest a specific role for the repeat element in neuronal development and synaptic function that is largely independent of RFC1 protein expression and its canonical functions. These findings have important implications for therapy development in this currently untreatable disorder.

CANVAS arises from a polymorphic set of biallelic *RFC1* repeat expansion motifs comprising AAGGG, AAAGG, ACAGG, AAGGC, AGGGC, and AGAGG motifs in isolation or as heterozygous combinations (*8*, *9*, *12*, *13*). Furthermore, rare CANVAS patients harbor compound heterozygous monoallelic *RFC1* expansions combined with loss-of-function mutations that result in RFC1 haploinsufficiency from the nonexpanded allele (*18*–*20*). This, in combination with data indicating a recessive mode of inheritance, suggested to us and others that RFC1 loss of function is a pathogenic driver of CANVAS pathogenesis. However, we find that RFC1 expression is normal at both the mRNA and protein level in the context of biallelic expansions in differentiated human iPSC–derived neurons, with no changes in *RFC1* mRNA splicing, intron degradation, transcript isoform, or exon usage in CANVAS neurons compared to controls (Fig. 3, A and B, and fig. S6, A to C). Consistent with this, we observe no deficits in canonical RFC1 functions in either mitotic and post-mitotic CANVAS patient–derived cell types. RFC1 plays a key role in both DNA replication and DNA damage repair (*57*), but CANVAS patient–derived neurons do not accumulate DNA damage compared to controls, and they exhibit normal rates of recovery after UV-induced DNA damage (Fig. 3 and fig. S9). Deficits in the rate of cell division and proliferation were observed in fibroblasts

and neural progenitor cells from patients with CANVAS (Fig. 3C). However, these deficits were at least partly independent of RFC1 function, as they were not corrected by reprovision of RFC1 to fibroblasts of patients with CANVAS. Despite clinical genetic data strongly suggesting an RFC1 loss of function in CANVAS and our own data showing gene alterations and signaling dysfunction in CANVAS patient iPSC–derived neurons, we find no evidence for a loss of canonical RFC1 function in multiple cell types, pointing toward a more nuanced etiology driving CANVAS pathogenesis at the *RFC1* locus.

Repeat associated gain-of-function mechanisms, such as the formation of RNA foci and the presence of ubiquitinated inclusions of RAN translated repeat polypeptides, occur in many repeat expansion neurologic diseases (*35*, *42–44*, *60*). Postmortem and cell-based analyses performed to date have not reliably demonstrated these pathological features in CANVAS (*7*, *8*), although recent reports do suggest the appearance of RNA foci in the context of certain repeat motifs (*32*). We detected sense strand AAGGG repeat-derived pentapeptide repeat peptides in cerebellar granule cells in three of four CANVAS patient brains examined (Fig. 2F and fig. S4). We did not detect these proteins or repeat RNA foci in patient iPSC–derived neurons and overexpression of AAGGG repeats in isolation did not induce toxicity in rodent neurons (Fig. 2, B and G, and fig. S3B). It is therefore unclear what role repeat RNA or translated pentapeptide repeats play in the neuropathogenic cascades that drive CANVAS pathogenesis.

CANVAS patient iPSC–derived neurons showed significant deficits in synaptic gene expression at both the mRNA and protein level compared to controls. CANVAS patient–derived neurons exhibit significant dysregulation in genes associated with pathways regulating synaptic structure and organization, regulation of chemical synaptic transmission, and expression of ion channels localized to both the pre- and postsynaptic membranes (Figs. 4 and 5 and fig. S10). This deficit correlates with phenotypic deficits in synaptic activity (Figs. 5 to 7). While synaptic dysfunction and loss of synaptic connections occur in numerous neurodegenerative diseases, such as Huntington disease (*61*, *62*), *C9orf72* FTD/ALS (*63*, *64*), and Alzheimer disease (*65*, *66*), the molecular steps preceding this deficit in synaptic gene expression and associated synaptic dysfunction in neurons of patients with CANVAS are unclear. Monoallelic deletion of the AAGGG expansion significantly corrected both synaptic gene expression defects and synaptic signaling dysfunction in CANVAS neurons and mimics the transcriptomic signature observed in a heterozygous case (Fig. 6). As this isogenic deletion of a single AAGGG expansion does not alter RFC1 expression or neuronal response and recovery after UV-induced DNA damage (figs. S7 and S9), this rescue in synaptic gene expression and neuronal connectivity instead appears to be dependent on the repeat element itself.

If dysfunction in RFC1 protein expression or activity was the cause of CANVAS phenotypes, then we would predict that synaptic dysfunction and gene expression alterations in CANVAS patient–derived neurons would be replicated by knockdown of RFC1 in control neurons. However, sustained (~10 weeks) knockdown of RFC1 with two independent shRNAs in control iPSC–derived neurons elicits no deficits in synaptic function or gene expression changes akin to those seen in CANVAS neurons (Fig. 7 and figs. S11, S13, and S14). Similarly, if RFC1 loss impacts CANVAS disease-relevant phenotypes, then prolonged reprovision of RFC1 protein into CANVAS patient iPSC–derived neurons should rescue the functional synaptic phenotypes observed. Yet, we observed no correction of CANVAS neuronal transcriptomic signatures after RFC1 reprovision (Fig. 7 and fig. S15). Moreover, while minor changes were observed in CANVAS neuronal activity with exogenous RFC1 expression, these improvements were both modest and nonspecific, with similar effects in control and CANVAS neurons. CANVAS neurons remained highly dysregulated even when expressing RFC1 (Fig. 7 and fig. S11).

One confounding aspect of *RFC1*-repeat expansion associated disorders is the discordance between expression of *RFC1* mRNA and protein and development of CANVAS. RFC1 expression is largely normal in most CANVAS cases, but is reduced in rare compound heterozygous CANVAS cases with one disease-associated repeat allele and one truncating mutant in *RFC1*. Our data suggest that RFC1 reduction alone fails to recapitulate synaptic, transcriptomic, and physiological phenotypes we observe in iPSC-derived glutamatergic neurons and RFC1 protein repletion is insufficient to rescue these same phenotypes in CANVAS neurons. However, removal of a single repeat allele on one CANVAS iNeuron line was sufficient to correct these same phenotypes. These clinical data and the iNeuron experimental observations appear to contradict one another. We assessed for a number of potential explanations for this contradiction, but found no evidence of mis-splicing or aberrant transcript generation from *RFC1* in the setting of the repeat expansion. However, one observation suggests a direction for future work: We were unable to remove the repeat and *Alu* element by CRISPR editing on both alleles in either control or CANVAS lines, despite extensive attempts to do so. A similar observation was made by a second group who generated heterozygous isogenic iPSCs (*29*). These data suggest that the repeat or the DNA region with which it is associated may have a normal function that is altered in the setting of larger and different repeat sequences. Thus, a homozygous AAGGG repeat may indeed induce disease through a loss-of-function mechanism, but just not through one that is dependent on RFC1 protein expression.

This manuscript has some limitations. First, there are a limited number of CANVAS and control cell lines analyzed and only a single isogenic control is included. Thus, this work is underpowered to identify all disease-relevant phenotypes and precluded consideration of sex as an independent variable in analysis. Second, we differentiated our iPSCs into predominantly glutamatergic neurons that most closely resemble cortical neurons rather than the sensory neurons or Purkinje neurons that are most impacted in the disease state. Last, we looked at relatively early developmental phenotypes in these iNeurons, which may be distinct from what occurs in a late-onset neurodegenerative disease such as CANVAS. However, it is worth noting that stem cell models of other neurodegenerative disorders exhibit developmental phenotypes that precede later evidence of neurodegeneration (*67–70*). Future work will be needed to confirm and extend these findings in more robust model systems to assess whether reduction in RFC1 or overexpression of AAGGG/CCTTT repeats are important in other aspects of disease pathogenesis and whether this synaptic dysfunction contributes to neuronal loss.

In summary, our studies provide support for a repeat-dependent mechanism of neuronal dysfunction in CANVAS that operates outside of the canonical functions of RFC1 protein. These findings stand in contrast to clinical genetic studies pointing toward an RFC1 loss-of-function mechanism as a central contributor to the molecular etiology of CANVAS and suggest that replacing or boosting canonical RFC1 function would be ineffective as a therapeutic approach in this

condition. The mechanisms by which these repetitive elements act to elicit disease do not fit easily into known repeat-associated gain-of-function and loss-of-function boxes previously defined for other repeat expansion disorders. Instead, our findings suggest that these nonreference repeats act through an as-yet undefined molecular mechanism that will likely have relevance beyond this condition.

MATERIALS AND METHODS

Dermal fibroblast isolation and iPSC derivation from samples of patients with CANVAS

Control iPSC lines were obtained from published sources (table S4). Dermal fibroblasts were obtained under institutional review board (IRB) protocol HUM00030934 after informed consent from patients clinically diagnosed with CANVAS spectrum disorder and genetically confirmed to have biallelic *RFC1* expansions or their relatives. Dermal biopsy samples were cultured in fibroblast medium [Dulbecco's modified Eagle's medium (DMEM), 10% (v/v) fetal bovine serum (FBS), 1% (v/v) 100× nonessential Amino Acid (NEAA), 1% (v/v) penicillin/streptomycin, and 1.5% (v/v) 1 M Hepes] at 37°C and 5% CO_2 until fibroblasts emerged from the tissue and were maintained at low passage number and grown to 80 to 90% confluence before episomal reprogramming. On day 0, fibroblasts were detached with Trypsin-EDTA, washed with (−/−) phosphate-buffered saline (PBS), and 3×10^5 cells were resuspended in 120 μl of R-buffer (Invitrogen) supplemented with 1 μg of GFP plasmid, 1 μg of pCXLE-hUL (Addgene no. 27080), 1 μg of pCXLE-hSK (Addgene no. 27078), and 1 μg of pCXLE-hOCT3/4-shP53 (Addgene no. 27077) and electroporated using the Neon System (Invitrogen, condition: 1450 V, 10 ms, three pulses). Electroporated fibroblasts were subsequently plated across three wells of a Geltrex (Thermo Fisher Scientific)–coated six-well plate with daily media changes of fresh fibroblast media until day 3. On day 3, cells were changed into a 50/50 ratio of fibroblast media and TeSR E-7 Reprogramming Media (STEMCELL Technologies), and on day 5, they were changed into 100% E-7 Reprogramming Media. Daily media changes of E7 Reprogramming Media followed until the emergence of iPSC colonies between days 21 and 28 when iPSC colonies were picked and transferred to Geltrex-coated 12-well plates containing 1 ml of TeSR-E8 (STEMCELL Technologies) supplemented with 10 μM ROCK Inhibitor (Y-27632, Cayman Chemical) for 24 hours. iPSCs were maintained in TeSR-E8 at 37°C and 5% CO_2 and passaged with 0.5 mM EDTA as needed. Multiple iPSC colonies were picked, expanded, and characterized per patient line before use. iPSC colonies were stained via ICC for pluripotency markers using antibodies against SOX2 (ab5603), OCT4 (ab181557), and Nanog (ab21624), and mRNA expression of pluripotency transcription factors was confirmed by RT-PCR (fig. S1, A and B). iPSCs were confirmed to have no chromosomal abnormalities by G-band karyotyping (fig. S1A, WiCell Research Institute). A detailed description of all cell lines, patient samples, and reagents used in these studies is provided in table S4.

Generation of heterozygous isogenic iPSC lines by CRISPR-Cas9

CRISPR gRNAs were designed to remove the expanded AAGGG repeat by nonhomologous end joining (NHEJ) utilizing the closest unique PAM sites to the repeat region and obtained from IDT (Fig. 1B). iPSCs were detached to a single-cell suspension using Accutase (Thermo Fisher Scientific), and 1×10^6 cells were resuspended in 120 μl of R-buffer containing preformed Cas9 RNP complexes (20 μM HiFi Cas9 (IDT), tracR-ATTO[550] (IDT), and gRNAs: F: GAGAATAGCAACGGTGTAGCTGG, R: TCATTTTCTGAAATACGGACAGG). The iPSC:RNP mix was electroporated using the Neon system (condition: 1450 V, 10 ms, three pulses), before plating across three wells of a Geltrex-coated six-well plate with TeSR-E8 media supplemented with 10 μM ROCK inhibitor (Y-27632) for 24 hours. Electroporation efficiency was assessed by nuclear positive fluorescence of TracR ATTO[550], and iPSCs were cultured until healthy colonies emerged. Clonal populations were achieved through single-cell isolation and expansion (*71*). Emerging clonal colonies were screened by PCR utilizing primer sets that spanned the repeat, priming either inside or outside the deletion region (Fig. 1C and table S1). Utilizing these primer sets, lack of amplification using primer set 1 in conjunction with amplification using primer set 2 indicates a biallelic expansion at this locus, and amplification with both primer sets indicates either a biallelic WT locus or the presence of a heterozygous monoallelic expansion when combined with a positive saw-tooth–like pattern by repeat-primed PCR. Last, reduced molecular weight amplification utilizing primer set 1 and full-length amplification with primer set 2 in combination with a positive saw-tooth–like pattern by repeat-primed PCR indicates a monoallelic deletion of this locus by CRISPR-Cas9, and reduced molecular weight amplification with primer set 1 and the absence of amplification with primer set 2 indicate a biallelic deletion of this locus byCRISPR-Cas9. Successful deletion of a single allele was achieved in CANVAS patient 1 iPSCs (Fig. 1C), and the specificity of this deletion was investigated by Sanger sequencing of long-range PCR products (Fig. 1D). Low-pass whole-genome sequencing of CANVAS patient 1 and its isogenic control did not reveal any deletions or insertions or mutations in known cancer-causing genes, or expected off-target loci.

RFC1 repeat screening, repeat-primed PCR, and Oxford nanopore sequencing

Cell pellets were obtained from patient-derived cell lines by Accutase detachment and centrifugation, or from patient postmortem brain tissue through gentle neutral protease digestion of brain tissue chunks for 45 min at 37°C, followed by mechanical dissociation to single-cell slurry by trituration. Genomic DNA was subsequently isolated from cell pellets using the gDNA mini-prep kit (Zymo). Screening for repeat expansions in *RFC1* was achieved by a combination of short-range repeat-spanning end-point PCR (table S1) with 2× Faststart PCR Mastermix (Roche) and by Repeat-Primed PCR using 2× Phusion Flash High-Fidelity PCR Mastermix (Thermo Fisher Scientific). End-point PCR was achieved by amplifying across the repeat region where the presence of a single band at the expected size indicated a nonexpanded WT locus, whereas the absence of a band in the presence of a control band utilizing primers that amplified the intronic region adjacent to the repeat locus indicated the presence of a large, expanded region (Fig. 1C). Repeat primed PCR of the repeat locus was conducted as previously described (*8*). 5′-FAM–labeled PCR products were analyzed through capillary electrophoresis by Laragen Inc., and files were analyzed by Peak Scanner Fragment Analysis Software (fig. S1A). Primers and cycling conditions for all PCR/RT-PCR experiments are outlined in table S1.

RFC1 repeat sizing by Nanopore is the subject of a separate manuscript (*30*). Briefly, we used a multiplexed CRISPR-based targeted Nanopore sequencing platform coupled with a modified profile

hidden Markov model STR repeat length caller to measure repeat expansion sizes at the *RFC1* locus (*30*). High–molecular weight genomic DNA was extracted using the Monarch HMW DNA Extraction Kit (T3050L, NEB) and treated with proteinase K before the application of nCATs-based CRISPR targeting disease-associated repeats, including *RFC1*. After incubation of gDNA with the Cas9 RNP and guide pool, RNA was processed for adapter ligation with T4 Ligase and captured using 0.3× Ampure beads (SQK-LSK114, ONT). Eluted long-fragment DNA was loaded onto R10.4.1 MinION flow cells following standard ONT protocols and sequenced for 72 hours.

Differentiation and maintenance of patient iPSC–derived neurons

iPSCs were differentiated to neural precursor cells (NPCs) and glutamatergic forebrain neurons using a modified dual-SMAD inhibition protocol (*72*) (fig. S1, C to E). Briefly, iPSCs were detached and plated as a monolayer at 100% confluence in TeSR-E8 media containing 10 μM ROCK Inhibitor (Y-27632) for 24 hours. On day 2, media was changed to 3N + A Media (241 ml of DMEM/F12 with Hepes + L-glutamine, 241 ml of Neurobasal Medium, 5 ml of NEAA, 2.5 ml of N2 supplement, 5 ml of B27 (− Vitamin A), 2.5 ml of Glutamax, 2.5 ml of penicillin/streptomycin, 125 μl of insulin, and 3.5 μl of BME) supplemented with two SMAD inhibitors: 10 μM SB431542 (Cayman Chemical) and 1 μM dorsomorphin (Cayman Chemical). On days 10 to 12, neuroepithelial sheets were combed into large clumps, passaged, and maintained on Geltrex-coated plates in 3N + A media supplemented with fibroblast growth factor (FGF; 20 ng/ml), with daily media changes until neuronal rosettes appeared (fig. S1D). Rosettes were manually picked and dissociated into single-cell NPCs using Accutase and plated on to Geltrex-coated plates in neural expansion medium [3N + A containing FGF (20 ng/ml) and epidermal growth factor (20 ng/ml)] with media changes every other day and passaged as needed using Accutase. Neuronal differentiation was achieved by plating high-density NPCs and switching to Neuronal Induction Media [490 ml of Brainphys, 10 ml of SM1, 5 ml of N2, brain-derived neurotrophic factor (20 ng/ml), glial-derived neurotrophic factor (20 ng/ml), 200 nM L-ascorbic acid, and 1 mM bucladesine]. Neurons were maintained with half medium changes twice per week, with Neuronal Induction Media supplemented with laminin (1 μg/ml) once per week. After 3 weeks of neuronal induction, post-mitotic neurons were detached using Accutase and replated at a uniform density on polyethyleneimine (PEI) and laminin-coated plates before experimentation. Efficient neuronal differentiation was further confirmed with Neu-N staining (fig. S1E). A detailed description of all cell lines and reagents used in these studies is provided in table S4.

Lentivirus production and transduction of iPSC-derived neurons

pLV-eF1-RFC1-eGFP full-length RFC1 expression plasmid was designed and synthesized by VectorBuilder, and pSMART-eF1-RFC1shRNA-tGFP expression plasmids were designed and produced by Horizon Discovery (table S4). Plasmids were prepped for lentiviral production by bacterial transformation followed by maxiprep. Plasmid DNA (80 μg) was used for lentiviral particle generation at University of Michigan Viral Vector Core. Lentivirus particles were delivered resuspended in DMEM media at 100× concentration (1×10^8 TU/ml). Lentivirus transduction of patient iPSC–derived neurons occurred on days in vitro (DIV) 7 after differentiation: Neurons were first treated with polybrene (1 μg/ml) for 30 min, before being washed with fresh complete Brainphys media and subsequently changed to conditioned media containing lentiviral particles diluted to their working concentration. Neurons were changed to fresh media 24 hours after transduction, and fluorescently positive cells appeared ~72 hours after transduction. Efficiency of lentiviral transduction is assessed in fig. S8.

Generation of repeat-containing CANVAS DNA reporter plasmids

Plasmids containing expanded AAGGG/AAAAG or CCCTT/CTTTT repeats were generated using RDL (*31*). Briefly, gene blocks containing 16 repeats flanked by type IIS restriction sites were produced by Genewiz (Azenta), and these were ligated into pcDNA3.1 plasmids mutated to remove restriction sites of interest. The repeat size was recursively doubled through a process of plasmid digestion to remove the repeat unit, followed by re-ligation back into the donor plasmid that had been nicked open at the 3′ end of the repeat unit. When the repeats were built to the maximal length before notable repeat shrinkage was observed, the repeats were removed by restriction endonuclease digestion and ligated into pcDNA3.1 reporter plasmids containing 100 nt of sense or antisense intronic sequence (Fig. 2A).

Longitudinal survival assays of primary rodent and iPSC-derived neurons

Mixed cortical neurons were dissected from E20 Long-Evans rat pups of both sexes under University of Michigan (UM)-approved The Institutional Animal Care and Use Committee (IACUC) protocols, as previously described (*39*–*41*). Cortical neurons were cultured at 0.6×10^6 cells ml^{-1} on 96-well plates. Cultures were maintained at 37°C in Neuronal Growth Media [NGM—Neurobasal-A supplemented with 2% (v/v) B-27 and 1% (v/v) Glutamax (Thermo Fisher Scientific)]. On DIV 4, neurons were cotransfected with 0.1 μg of pGW1-mApple and either 0.1 μg of pGW1-GFP or 0.1 μg of experimental DNA per well of a 96-well culture plate, using Lipofectamine 2000 (Invitrogen). Neurons were imaged at regular 24-hour intervals starting 24 hours after transfection using an automated fluorescence microscopy platform detailed in earlier studies (*39*–*41*). Image processing for each time point and survival analysis for automated fluorescence images were achieved by custom code written in Python or the ImageJ macro language, and cumulative hazard plots were generated using the survival package in R.

Postmortem brain IHC and antibody generation

Rabbit polyclonal antibodies were generated by Abclonal (Cambridge, MA) against peptides containing $(KGREG)_7$ or $(PFPSL)_7$, corresponding to $(AAGGG)_{21}$ and $(CCCTT)_{21}$, respectively. Antibodies were affinity purified from anti-sera. Prebleed sera and peptides were used to characterize the antibodies, and protein concentrations of the prebleed sera were determined. The dilution of prebleed sera used had an equal protein concentration as the dilution of antibody used. A 100× excess of peptide was incubated with the antibody before use to block the antibodies' ability to bind to antigen.

CANVAS and control postmortem brain tissue was obtained with informed consent of the patients or their relatives and the approval of the local IRBs from the University of Michigan Brain Bank

(IRB: HUM00041576), Mass General Brigham Stem Cells in Neurodegeneration Center (IRB: 2020P002649), UCL Queen Square Brain Bank for Neurological Disorders (REC: 08/H0718/54+5), and The Netherlands Brain Bank (IRB: IRB00002991). For immunohistochemistry, paraffin-embedded sections were deparaffinized with a series of xylene washes and decreasing concentrations of ethanol. Antigen retrieval (AR) was achieved with citrate buffer, if necessary. Endogenous peroxidase was quenched with 1% hydrogen peroxide for 30 min. Sections were blocked in 5% normal goat serum (NGS) in tris, pH 7.6. Primary antibodies $KGREG_7$ (Abclonal, 1:100, Acid AR) and $PFPSL_7$ (Abclonal, 1:100, Acid AR) were diluted in 5% NGS tris B (tris, pH 7.6, 0.1% Triton X-100, and 0.5% bovine serum albumin) and were incubated with sections overnight at 4°C. The following day, sections were washed in tris A (tris, pH 7.6, 0.1% Triton) and tris B. Antibody detection was determined using VECTASTAIN Elite ABC HRP kit (Vector Laboratories) following the manufacturer's protocol. Sections were counterstained with hematoxylin (Vector Laboratories). After dehydrating the slides through a series of increasing concentration of ethanol and then xylenes, coverslips were mounted to the slides using DPX mounting media. Images were taken on an Olympus BX51 microscope. A detailed description of all patient samples used in these studies are provided in table S4.

RNA hybridization chain reaction of RNA foci

CANVAS patient and control iPSC–derived neurons were differentiated and maintained to 8 weeks of age as previously described. Cells were washed with (−/−) PBS and subsequently fixed with 4% paraformaldehyde (PFA) and incubated overnight in 70% ethanol. After overnight incubation with 70% ethanol, cells were rehydrated in PBS for 1 hour, permeabilized with 0.1% Triton X-100 for 5 min, and blocked with 2% bovine serum albumin (BSA) for 20 min at room temperature. Repeat-containing RNA foci were probed in patient iPSC–derived neurons using DNA probes with additional sequence complementary to Cy5-labeled, self-hybridizing hairpins (*73*–*76*). Probes against the AAAAG, AAGGG, CTTTT, and CCCTT sequences (table S2) were purchased from molecularinstruments.org and applied according to the manufacturer's protocol. Coverslips were then applied to slides with ProLong Gold Antifade Mounting Medium with DAPI (4′,6-diamidino-2-phenylindole). Then, 10 to 20 fields per condition were imaged blinded using an inverted, Olympus FV1000, laser-scanning confocal microscope. Channels were imaged sequentially and optimized to eliminate bleed-through. Neurons were imaged in a series of *Z*-planes to resolve the entire soma and dendritic arbor. Images were analyzed blinded in ImageJ. Average intensity composite images were derived from raw image files. For quantification of individual soma, cell casts were made using threshold images as a guide.

Analysis of DNA damage accumulation and recovery in iPSC-derived neurons

The accumulation of basal DNA damage was analyzed in 8-week-old patient iPSC–derived neurons by Western blot for the DNA damage marker γ-H2AX (P-S139, Abcam ab11174). The ability to detect DNA damage was tested in patient iPSC–derived neurons by exposure to 0, 15, 30, and 120 mJ/cm^2 UV irradiation to induce differing degrees of DNA damage, followed by Western blot analysis for γ-H2AX. Recovery after DNA damage induction in CANVAS patient iPSC–derived neurons was conducted as described: CANVAS patient and control iPSC–derived neurons were plated on PEI/laminin-coated glass chamber slides and maintained for 8 weeks as previously described. At 8 weeks, neurons were either fixed with 4% PFA for no-exposure control or irradiated with 60 mJ/cm^2 UV. UV-irradiated cells were fixed in 4% PFA at time intervals of 0.5, 1, 3, 6, 12, and 24 hours after irradiation and were stained for the neuronal nuclei marker NeuN, DNA marker DAPI, and DNA damage marker γ-H2AX. Images were captured in all three fluorescent channels at the same exposure for each cell line and time point at 40× magnification with an Olympus IX71 fluorescent microscope and Slidebook 5.5 software, and γ-H2AX reactivity was analyzed using FIJI. High-throughput quantification of cells was achieved by using a FIJI script to mask NeuN-positive nuclei using the parameters of size 200 to 1500 pixels^2 and circularity 0.40 to 1.00. Masked NeuN-positive nuclei were subsequently analyzed for γ-H2AX intensity and was quantified and visualized using GraphPad Prism v.9.

Cell proliferation assays

CANVAS patient and control fibroblasts or NPCs were analyzed for cell proliferation rates through automated longitudinal bright-field microscopy using the Incucyte S3 system. Cells were seeded into 96-well plates (Techno Plastic Products) at a density of 2500 cells per well and whole wells were imaged by tiling of 5× images per well using S3 Neuro O/NIR Optical Module 10× magnification with 6-hour intervals for a period of 10 days. Cell confluence was automatically analyzed using the Incucyte S3 Live Cell Analysis Software using the basic analyzer cell confluence settings to mask and quantify individual cell number and absolute confluence with the following parameters: segmentation adjustment, 2; hole fill, 0 μm^2; and adjust size, 4 pixels. Three control and four CANVAS patient fibroblast lines were used, with 12 wells averaged per fibroblast line for technical replicates within each biological replicate, and a total of three biological replicates were presented. For lentiviral experiments, fibroblasts were transduced with lentiviral particles in 10-cm dish mass culture 7 days before re-seeding at experimental density in 96-well plates. Lentiviral particles were diluted within culture medium to a final concentration of 1×.

Calcium imaging and analysis of iPSC-derived neurons

TPP tissue culture–treated 96-well plates were prepared by coating with 0.1% PEI diluted in borate buffer for 24 hours, followed by 3× washes in H_2O and coating with laminin (1 μg/ml) diluted into neuronal medium overnight before use. Rat primary cortical astrocytes (Thermo Fisher Scientific) were plated at high density and maintained until confluence in Astrocyte Media [DMEM/F12, 10% (v/v) horse serum, 10% (v/v) FBS, 3% (v/v) 20% D-glucose solution, 2% (v/v) B27 supplement, 1% (v/v) Glutamax, and 1% (v/v) penicillin/streptomycin antibiotic]. CANVAS and control iPSC–derived neurons were differentiated from NPCs in Geltrex-coated 10-cm plates as previously described for 21 days. Five days after differentiation, neurons were transduced with lentiviral particles as previously described based on experimental setup. After 3 weeks of neuronal differentiation, neurons were detached using 25% Accutase and incubating at 37°C for 10 min. Neurons were diluted in prewarmed Brainphys neuronal media and gently triturated to detach. Neurons were pelleted by centrifugation and resuspended in 1 ml of prewarmed Brainphys neuronal media before passing through a 45-μm cell strainer to obtain a single-cell suspension of neuronal soma. Cells were counted using a hemocytometer with trypan blue as a live

cell marker, and neurons were diluted to a concentration of 5000 live cells in 200 μl of complete neuronal media per well of a 96-well plate. Ten days after replating, neurons were transduced with Incucyte Neuroburst Orange Lentivirus particles by diluting 5 μl of lentivirus into complete Brainphys neuronal media per well, and neurons were maintained as previously described until analysis using the Incucyte S3 Live Cell Analysis Instrument. Image acquisition was conducted using S3 Neuro O/NIR Optical Module at 4× magnification for 180 s per well in phase and orange fluorescence channels, and data were automatically analyzed using the Incucyte S3 Live Cell Analysis Software with the following parameters: object size, 30 μm; min cell width, 9 μm; sensitivity, 1; and min burst intensity, 0.2. Neurons were analyzed for active cell number, basal calcium intensity, burst rate, burst strength, burst duration, and network correlation. Data for different neuronal signaling metrics were compiled for each scan time point and analyzed using GraphPad Prism v.9.

RNA extraction and RT-PCR/qPCR

Total RNA was extracted using TRIzol and chloroform, followed by isopropanol precipitation. RNA pellets were washed in 75% + ethanol and resuspended in nuclease free H_2O. Contaminating gDNA was removed by DNase I digestion (Thermo Fisher Scientific) and cleaned up using an RNA clean and concentrator kit (Zymo). Total RNA was quantified by NanoDrop UV/Vis spectrophotometry, and 1 μg of RNA was reverse-transcribed to cDNA using iScript cDNA synthesis kit (Bio-Rad) with oligo-dT primers for poly-A mRNA selection. RT-PCR was achieved using 2× Faststart PCR Mastermix (Roche) using primer sets and cycling conditions described in table S1. RT-qPCR was achieved using Taqman Real-Time PCR Assay(s) (Thermo Fisher Scientific) using primer probe sets and cycling conditions described in table S1.

RNA-seq sample preparation and analysis

For RNA sequencing (RNA-seq) experiments, patient iPSC–derived neurons were plated and aged to ~10 weeks as previously described. Total RNA was extracted using TRIzol and chloroform, followed by isopropanol precipitation. RNA pellets were washed in 75% + ethanol and resuspended in nuclease-free H_2O. Contaminating gDNA was removed by DNase I digestion (Thermo Fisher Scientific) and cleaned up using an RNA clean and concentrator kit (Zymo). Total RNA was quantified by NanoDrop UV/Vis spectrophotometry, analyzed for integrity by Bioanalyzer, and 1.5 to 2 μg total RNA was sent to Genewiz (Azenta) for rRNA-depletion library preparation and paired-end sequencing, where between 20 million and 30 million paired-end reads were achieved per sample. Raw FASTA read files were delivered by sFTP transfer and analyzed in Command Line and R-Studio. Read files were quality controlled using FastQC(v0.12.0) (*77*), preprocessed and trimmed using fastp(v0.23.4) (*78*), and aligned to GRCh38 (NCBI GCF_000001405.40) genome using STAR(v2.7.11a) two-pass alignment (*79*). Transcript and gene count matrices were produced using featureCounts (Subread v2.0.6) (*80*). Read count matrices were imported into R-Studio (vRtidyverse/4.2.0) where count normalization and differential gene expression analysis was conducted using DESeq2(v1.40.2) (*81*). GO Pathway Overrepresentation analysis was conducted using GOstats(v2.66.0) (*82*), and GSEA was conducted using clusterProfiler(v4.8.3) (*83*) and gseGO (v3.0.4) (*83*). Plots were generated and visualized using DESeq2(v1.40.2) (*81*), EnhancedVolcano(v1.18.0) (*84*), ggPlot2(v3.4.3) (*85*), and ggRidges(v0.5.4) (*86*). Differential exon usage was analyzed and plotted using DEXSeq(v1.46.0) (*56*), and circular RNA analysis was conducted using DCC(v0.5.0) in Python (*87*). Sashimi plots of splice junction analysis were produced using the Integrative Genomics Viewer Genome Browser (*88*) and exported for modification in Illustrator (Adobe). All harvesting and sequencing of RNA were conducted at the same time within each experimental condition to avoid batch effects. Genes and pathways that showed significant correction in the heterozygous isogenic corrected line from CANVAS to control were identified by binning normalized gene expression values into categories of those showing reduced expression upon heterozygous deletion, 0 to 50% positive correction in expression compared to control, 50 to 150% positive correction in expression compared to control, and those genes that showed over 150% correction in comparison to control. These genes were further binned into their statistical significance in difference of expression when compared to control and plotted using ggPlot2(v3.4.3) (*85*), and GO Pathway Overrepresentation analysis was conducted using GOstats(v2.66.0) (*81*).

Supplementary Materials

The PDF file includes:
Figs. S1 to S15
Tables S1 to S4
Legends for movies S1 to S3

Other Supplementary Material for this manuscript includes the following:
Movies S1 to S3

REFERENCES AND NOTES

1. A. Cortese, R. Curro', E. Vegezzi, W. Y. Yau, H. Houlden, M. M. Reilly, Cerebellar ataxia, neuropathy and vestibular areflexia syndrome (CANVAS): Genetic and clinical aspects. *Pract. Neurol.* **22**, 14–18 (2022).
2. D. J. Szmulewicz, L. Roberts, C. A. McLean, H. G. MacDougall, G. Michael Halmagyi, E. Storey, Proposed diagnostic criteria for cerebellar ataxia with neuropathy and vestibular areflexia syndrome (CANVAS). *Neurol. Clin. Pract.* **6**, 61–68 (2016).
3. C. Paisán-Ruiz, J. C. Jen, CANVAS with cerebellar/sensory/vestibular dysfunction from RFC1 intronic pentanucleotide expansion. *Brain* **143**, 386–390 (2020).
4. R. Ishai, M. Seyyedi, A. M. Chancellor, C. A. M. Lean, M. L. Rodriguez, G. M. Halmagyi, J. B. Nadol Jr., D. J. Szmulewicz, A. M. Quesnel, The pathology of the vestibular system in CANVAS. *Otol. Neurotol.* **42**, e332–e340 (2021).
5. D. J. Szmulewicz, L. Roberts, C. A. McLean, H. G. MacDougall, A. M. Chancellor, S. Mossman, D. Lamont, E. Storey, G. M. Halmagyi, Clinico-pathological correlates in cerebellar ataxia with neuronopathy and vestibular areflexia syndrome (CANVAS). *J. Neurol. Sci.* **333**, e632 (2013).
6. D. J. Szmulewicz, C. A. M. Lean, M. L. Rodriguez, A. M. Chancellor, S. Mossman, D. Lamont, L. Roberts, E. Storey, G. M. Halmagyi, Dorsal root ganglionopathy is responsible for the sensory impairment in CANVAS. *Neurology* **82**, 1410–1415 (2014).
7. V. Khurana, C. M. de Gusmao, M. Glover, J. Helgager, Case 20-2021: A 69-year-old man with ataxia. *N. Engl. J. Med.* **385**, 165–175 (2021).
8. A. Cortese, R. Simone, R. Sullivan, J. Vandrovcova, H. Tariq, Y. W. Yan, J. Humphrey, Z. Jaunmuktane, P. Sivakumar, J. Polke, M. Ilyas, E. Tribollet, P. J. Tomaselli, G. Devigili, I. Callegari, M. Versino, V. Salpietro, S. Efthymiou, D. Kaski, N. W. Wood, N. S. Andrade, E. Buglo, A. Rebelo, A. M. Rossor, A. Bronstein, P. Fratta, W. J. Marques, S. Züchner, M. M. Reilly, H. Houlden, Biallelic expansion of an intronic repeat in RFC1 is a common cause of late-onset ataxia. *Nat. Genet.* **51**, 649–658 (2019).
9. H. Rafehi, D. J. Szmulewicz, M. F. Bennett, N. L. M. Sobreira, K. Pope, K. R. Smith, G. Gillies, P. Diakumis, E. Dolzhenko, M. A. Eberle, M. G. Barcina, D. P. Breen, A. M. Chancellor, P. D. Cremer, M. B. Delatycki, B. L. Fogel, A. Hackett, G. M. Halmagyi, S. Kapetanovic, A. Lang, S. Mossman, W. Mu, P. Patrikios, S. L. Perlman, I. Rosemergy, E. Storey, S. R. D. Watson, M. A. Wilson, D. S. Zee, D. Valle, D. J. Amor, M. Bahlo, P. J. Lockhart, Bioinformatics-based identification of expanded repeats: A non-reference intronic pentamer expansion in RFC1 causes CANVAS. *Am. J. Hum. Genet.* **105**, 151–165 (2019).
10. A. Cortese, S. Tozza, W. Y. Yau, S. Rossi, S. J. Beecroft, Z. Jaunmuktane, Z. Dyer, G. Ravenscroft, P. J. Lamont, S. Mossman, A. Chancellor, T. Maisonobe, Y. Pereon,

C. Cauquil, S. Colnaghi, G. Mallucci, R. Curro, P. J. Tomaselli, G. Thomas-Black, R. Sullivan, S. Efthymiou, A. M. Rossor, M. Laurá, M. Pipis, A. Horga, J. Polke, D. Kaski, R. Horvath, P. F. Chinnery, W. Marques, C. Tassorelli, G. Devigili, L. Leonardis, N. W. Wood, A. Bronstein, P. Giunti, S. Züchner, T. Stojkovic, N. Laing, R. H. Roxburgh, H. Houlden, M. M. Reilly, Cerebellar ataxia, neuropathy, vestibular areflexia syndrome due to RFC1 repeat expansion. *Brain* **143**, 480–490 (2020).

11. R. Currò, N. Dominik, S. Facchini, E. Vegezzi, R. Sullivan, V. Galassi Deforie, G. Fernández-Eulate, A. Traschütz, S. Rossi, M. Garibaldi, M. Kwarciany, F. Taroni, A. Brusco, J.-M. Good, F. Cavalcanti, S. Hammans, G. Ravenscroft, R. H. Roxburgh; RFC1 repeat expansion study group, R. P. Schnekenberg, B. Rugginini, E. Abati, A. Manini, I. Quartesan, A. Ghia, A. L. de Munaìn, F. Manganelli, M. Kennerson, F. M. Santorelli, J. Infante, W. Marques, M. Jokela, S. M. Murphy, P. Mandich, G. M. Fabrizi, C. Briani, D. Gosal, D. Pareyson, A. Ferrari, F. Prados, T. Yousry, V. Khurana, S.-H. Kuo, J. Miller, C. Troakes, Z. Jaunmuktane, P. Giunti, A. Hartmann, N. Basak, M. Synofzik, T. Stojkovic, M. Hadjivassiliou, M. M. Reilly, H. Houlden, A. Cortese, Role of the repeat expansion size in predicting age of onset and severity in RFC1 disease. *Brain* **147**, 1887–1898 (2024).
12. C. K. Scriba, S. J. Beecroft, J. S. Clayton, A. Cortese, R. Sullivan, W. Y. Yau, N. Dominik, M. Rodrigues, E. Walker, Z. Dyer, T. Y. Wu, M. R. Davis, D. C. Chandler, B. Weisburd, H. Houlden, M. M. Reilly, N. G. Laing, P. J. Lamont, R. H. Roxburgh, G. Ravenscroft, A novel RFC1 repeat motif (ACAGG) in two Asia-Pacific CANVAS families. *Brain* **143**, 2904–2910 (2020).
13. N. Dominik, S. Magri, R. Currò, E. Abati, S. Facchini, M. Corbetta, H. MacPherson, D. D. Bella, E. Sarto, I. Stevanovski, S. R. Chintalaphani, F. Akcimen, A. Manini, E. Vegezzi, I. Quartesan, K.-A. Montgomery, V. Pirota, E. Crespan, C. Perini, G. P. Grupelli, P. J. Tomaselli, W. Marques, G. E. R. Consortium, J. Shaw, J. Polke, E. Salsano, S. Fenu, D. Pareyson, C. Pisciotta, G. K. Tofaris, A. H. Nemeth, J. Ealing, A. Radunovic, S. Kearney, K. R. Kumar, S. Vucic, M. Kennerson, M. M. Reilly, H. Houlden, I. Deveson, A. Tucci, F. Taroni, A. Cortese, Normal and pathogenic variation of RFC1 repeat expansions: Implications for clinical diagnosis. *Brain* **146**, 5060–5069 (2023).
14. A. Cortese, M. M. Reilly, H. Houlden, *RFC1 CANVAS/Spectrum Disorder* (University of Washington, GeneReviews, 2020); https://ncbi.nlm.nih.gov/books/NBK564656/.
15. A. Traschütz, A. Cortese, S. Reich, N. Dominik, J. Faber, H. Jacobi, A. M. Hartmann, D. Rujescu, S. Montaut, A. Echaniz-Laguna, S. Erer, V. C. Schütz, A. A. Tarnutzer, M. Sturm, T. B. Haack, N. Vaucamps-Diedhiou, H. Puccio, L. Schöls, T. Klockgether, B. P. van de Warrenburg, M. Paucar, D. Timmann, R.-D. Hilgers, J. Gazulla, M. Strupp, G. Moris, A. Filla, H. Houlden, M. Anheim, J. Infante, A. N. Basak, M. Synofzik; RFC1 Study Group, Natural history, phenotypic spectrum, and discriminative features of multisystemic RFC1 disease. *Neurology* **96**, e1369–e1382 (2021).
16. M. Gisatulin, V. Dobricic, C. Zühlke, Y. Hellenbroich, V. Tadic, A. Münchau, K. Isenhardt, K. Bürk, M. Bahlo, P. J. Lockhart, K. Lohmann, C. Helmchen, N. Brüggemann, Clinical spectrum of the pentanucleotide repeat expansion in the RFC1 gene in ataxia syndromes. *Neurology* **95**, e2912–e2923 (2020).
17. A. Martelli, M. Napierala, H. Puccio, Understanding the genetic and molecular pathogenesis of Friedreich's ataxia through animal and cellular models. *Dis. Model. Mech.* **5**, 165–176 (2012).
18. R. Ronco, C. Perini, R. Currò, N. Dominik, S. Facchini, A. Gennari, R. Simone, S. Stuart, S. Nagy, E. Vegezzi, I. Quartesan, A. El-Saddig, T. Lavin, A. Tucci, A. Szymura, L. E. N. D. Farias, A. Gary, M. Delfeld, P. Kandikatla, N. Niu, S. Tawde, J. Shaw, J. Polke, M. M. Reilly, N. W. Wood, E. Crespan, C. Gomez, J. Y. H. Chen, J. D. Schmahmann, D. Gosal, H. Houlden, S. Das, A. Cortese, Truncating variants in RFC1 in cerebellar ataxia, neuropathy, and vestibular areflexia syndrome. *Neurology* **100**, e543–e554 (2023).
19. A. Arteche-López, A. Avila-Fernandez, A. Damian, E. Soengas-Gonda, R. P. de la Fuente, P. R. Gómez, J. G. Merlo, L. H. Burgos, C. C. Fernández, J. M. L. Rosales, J. F. G. Martínez, J. F. Quesada-Espinosa, M. Corton, M. P. Guerrero-Molina, New cerebellar ataxia, neuropathy, vestibular areflexia syndrome cases are caused by the presence of a nonsense variant in compound heterozygosity with the pathogenic repeat expansion in the RFC1 gene. *Clin. Genet.* **103**, 236–241 (2023).
20. S. Weber, G. Coarelli, A. Heinzmann, M.-L. Monin, N. Richard, M. Gerard, A. Durr, V. Huin, Two RFC1 splicing variants in CANVAS. *Brain* **146**, e14–e16 (2023).
21. J. D. Cleary, A. Pattamatta, L. P. W. Ranum, Repeat-associated non-ATG (RAN) translation. *J. Biol. Chem.* **293**, 16127–16141 (2018).
22. A. Bajc Česnik, S. Darovic, S. Prpar Mihevc, M. Štalekar, M. Malnar, H. Motaln, Y.-B. Lee, J. Mazej, J. Pohleven, M. Grosch, M. Modic, M. Fonovič, B. Turk, M. Drukker, C. E. Shaw, B. Rogelj, Nuclear RNA foci from C9ORF72 expansion mutation form paraspeckle-like bodies. *J. Cell Sci.* **132**, jcs224303 (2019).
23. International Human Genome Sequencing Consortium, Initial sequencing and analysis of the human genome. *Nature* **409**, 860–921 (2001).
24. L. M. Payer, J. P. Steranka, D. Ardeljan, J. Walker, K. C. Fitzgerald, P. A. Calabresi, T. A. Cooper, K. H. Burns, Alu insertion variants alter mRNA splicing. *Nucleic Acids Res.* **47**, 421–431 (2019).
25. R. Sorek, G. Ast, D. Graur, Alu-containing exons are alternatively spliced. *Genome Res.* **12**, 1060–1067 (2002).
26. D. D. Y. Kim, T. T. Y. Kim, T. Walsh, Y. Kobayashi, T. C. Matise, S. Buyske, A. Gabriel, Widespread RNA editing of embedded Alu elements in the human transcriptome. *Genome Res.* **14**, 1719–1725 (2004).
27. L.-L. Chen, L. Yang, ALUternative regulation for gene expression. *Trends Cell Biol.* **27**, 480–490 (2017).
28. J. Robinson, F. Raguseo, S. P. Nuccio, D. Liano, M. Di Antonio, DNA G-quadruplex structures: More than simple roadblocks to transcription? *Nucleic Acids Res.* **49**, 8419–8431 (2021).
29. K. C. Davies, K. Bozaoglu, P. J. Lockhart, Generation and heterozygous repair of human iPSC lines from three individuals with cerebellar ataxia, neuropathy and vestibular areflexia syndrome (CANVAS) carrying biallelic AAGGG expansions in RFC1. *Stem Cell Res.* **68**, 103047 (2023).
30. K. Van Deynze, C. Mumm, C. J. Maltby, J. A. Switzenberg, P. K. Todd, A. P. Boyle, Enhanced detection and genotyping of disease-associated tandem repeats using HMMSTR and targeted long-read sequencing. medRxiv 24306681 [Preprint] (2024). https://doi.org/10.1101/2024.05.01.24306681.
31. K. J. Rohilla, K. N. Ovington, A. A. Pater, M. Barton, A. J. Henke, K. T. Gagnon, Systematic microsatellite repeat expansion cloning and validation. *Hum. Genet.* **139**, 1233–1246 (2020).
32. T. Wada, H. Doi, M. Okubo, M. Tada, N. Ueda, H. Suzuki, W. Tominaga, H. Koike, H. Komiya, S. Kubota, S. Hashiguchi, H. Nakamura, K. Takahashi, M. Kunii, K. Tanaka, Y. Miyaji, Y. Higashiyama, E. Koshimizu, S. Miyatake, M. Katsuno, S. Fujii, H. Takahashi, N. Matsumoto, H. Takeuchi, F. Tanaka, RNA foci in two bi-allelic RFC1 expansion carriers. *Ann. Neurol.* **95**, 607–613 (2024).
33. A. Krans, G. Skariah, Y. Zhang, B. Bayly, P. K. Todd, Neuropathology of RAN translation proteins in fragile X-associated tremor/ataxia syndrome. *Acta Neuropathol. Commun.* **7**, 152 (2019).
34. S. E. Wright, C. M. Rodriguez, J. Monroe, J. Xing, A. Krans, B. N. Flores, V. Barsur, M. I. Ivanova, K. S. Koutmou, S. J. Barmada, P. K. Todd, CGG repeats trigger translational frameshifts that generate aggregation-prone chimeric proteins. *Nucleic Acids Res.* **50**, 8674–8689 (2022).
35. C. M. Rodriguez, S. E. Wright, M. G. Kearse, J. M. Haenfler, B. N. Flores, Y. Liu, M. F. Ifrim, M. R. Glineburg, A. Krans, P. Jafar-Nejad, M. A. Sutton, G. J. Bassell, J. M. Parent, F. Rigo, S. J. Barmada, P. K. Todd, A native function for RAN translation and CGG repeats in regulating fragile X protein synthesis. *Nat. Neurosci.* **23**, 386–397 (2020).
36. F. He, B. N. Flores, A. Krans, M. Frazer, S. Natla, S. Niraula, O. Adefioye, S. J. Barmada, P. K. Todd, The carboxyl termini of RAN translated GGGGCC nucleotide repeat expansions modulate toxicity in models of ALS/FTD. *Acta Neuropathol. Commun.* **8**, 122 (2020).
37. I. Malik, Y.-J. Tseng, S. E. Wright, K. Zheng, P. Ramaiyer, K. M. Green, P. K. Todd, SRSF protein kinase 1 modulates RAN translation and suppresses CGG repeat toxicity. *EMBO Mol. Med.* **13**, e14163 (2021).
38. B. N. Flores, M. E. Dulchavsky, A. Krans, M. R. Sawaya, H. L. Paulson, P. K. Todd, S. J. Barmada, M. I. Ivanova, Distinct *C9orf72*-associated dipeptide repeat structures correlate with neuronal toxicity. *PLOS ONE* **11**, e0165084 (2016).
39. H. C. Archbold, K. L. Jackson, A. Arora, K. Weskamp, E. M.-H. Tank, X. Li, R. Miguez, R. D. Dayton, S. Tamir, R. L. Klein, S. J. Barmada, TDP43 nuclear export and neurodegeneration in models of amyotrophic lateral sclerosis and frontotemporal dementia. *Sci. Rep.* **8**, 4606 (2018).
40. K. Weskamp, N. Safren, R. Miguez, S. Barmada, Monitoring Neuronal Survival via Longitudinal Fluorescence Microscopy. *J. Vis. Exp.* 10.3791/59036 (2019).
41. A. M. Malik, R. A. Miguez, X. Li, Y.-S. Ho, E. L. Feldman, S. J. Barmada, Matrin 3-dependent neurotoxicity is modified by nucleic acid binding and nucleocytoplasmic localization. *eLife* **7**, e35977 (2018).
42. M. Niblock, B. N. Smith, Y.-B. Lee, V. Sardone, S. Topp, C. Troakes, S. Al-Sarraj, C. S. Leblond, P. A. Dion, G. A. Rouleau, C. E. Shaw, J.-M. Gallo, Retention of hexanucleotide repeat-containing intron in *C9orf72* mRNA: Implications for the pathogenesis of ALS/FTD. *Acta Neuropathol. Commun.* **4**, 18 (2016).
43. Ł. J. Sznajder, J. D. Thomas, E. M. Carrell, T. Reid, K. N. McFarland, J. D. Cleary, R. Oliveira, C. A. Nutter, K. Bhatt, K. Sobczak, T. Ashizawa, C. A. Thornton, L. P. W. Ranum, M. S. Swanson, Intron retention induced by microsatellite expansions as a disease biomarker. *Proc. Natl. Acad. Sci. U.S.A.* **115**, 4234–4239 (2018).
44. S. Wang, M. J. Latallo, Z. Zhang, B. Huang, D. G. Bobrovnikov, D. Dong, N. M. Livingston, W. Tjoeng, L. R. Hayes, J. D. Rothstein, L. W. Ostrow, B. Wu, S. Sun, Nuclear export and translation of circular repeat-containing intronic RNA in C9ORF72-ALS/FTD. *Nat. Commun.* **12**, (2021).
45. S. I. Bidichandani, T. Ashizawa, P. I. Patel, The GAA triplet-repeat expansion in friedreich ataxia interferes with transcription and may be associated with an unusual DNA structure. *Am. J. Hum. Genet.* **62**, 111–121 (1998).
46. E. D. Wieben, R. A. Aleff, X. Tang, M. L. Butz, K. R. Kalari, E. W. Highsmith, J. Jen, G. Vasmatzis, S. V. Patel, L. J. Maguire, K. H. Baratz, M. P. Fautsch, Trinucleotide repeat expansion in the transcription factor 4 (TCF4) gene leads to widespread mRNA splicing

changes in Fuchs' endothelial corneal dystrophy. *Invest. Ophthalmol. Vis. Sci.* **58**, 343–352 (2017).
47. N. Zhang, T. Ashizawa, RNA toxicity and foci formation in microsatellite expansion diseases. *Curr. Opin. Genet. Dev.* **44**, 17–29 (2017).
48. M. Nakama, H. Otsuka, Y. Ago, H. Sasai, E. Abdelkreem, Y. Aoyama, T. Fukao, Intronic antisense Alu elements have a negative splicing effect on the inclusion of adjacent downstream exons. *Gene* **664**, 84–89 (2018).
49. G. Lev-Maor, O. Ram, E. Kim, N. Sela, A. Goren, E. Y. Levanon, G. Ast, Intronic alus influence alternative splicing. *PLOS Genet.* **4**, e1000204 (2008).
50. M. Wang, J. Hou, M. Müller-McNicoll, W. Chen, E. M. Schuman, Long and repeat-rich intronic sequences favor circular RNA formation under conditions of reduced spliceosome activity. *iScience* **20**, 237–247 (2019).
51. R. Wang, X. Zhou, G. Luo, J. Zhang, M. Yang, C. Song, CircRNA RERE promotes the oxidative stress-induced apoptosis and autophagy of nucleus pulposus cells through the miR-299-5p/galectin-3 axis. *J. Healthc. Eng.* **2021**, 2771712 (2021).
52. A. Dal Molin, M. Hofmans, E. Gaffo, A. Buratin, H. Cavé, C. Flotho, V. de Haas, C. M. Niemeyer, J. Stary, P. Van Vlierberghe, J. Philippé, B. De Moerloose, G. te Kronnie, S. Bresolin, T. Lammens, S. Bortoluzzi, CircRNAs dysregulated in juvenile myelomonocytic leukemia: CircMCTP1 stands out. *Front. Cell Dev. Biol.* **8**, 613540 (2021).
53. Y. Wei, X. Chen, C. Liang, Y. Ling, X. Yang, X. Ye, H. Zhang, P. Yang, X. Cui, Y. Ren, X. Xin, H. Li, R. Wang, W. Wang, F. Jiang, S. Liu, J. Ding, B. Zhang, L. Li, H. Wang, A noncoding regulatory RNAs network driven by Circ-CDYL acts specifically in the early stages hepatocellular carcinoma. *Hepatology* **71**, 130–147 (2020).
54. Y. Chen, X. Li, S. Meng, S. Huang, S. Chang, J. Shi, Identification of functional circRNA–miRNA–mRNA regulatory network in dorsolateral prefrontal cortex neurons of patients with cocaine use disorder. *Front. Mol. Neurosci.* **15**, 839233 (2022).
55. Z. Luo, L. Lu, Q. Tang, W. Wei, P. Chen, Y. Chen, J. Pu, J. Wang, CircCAMSAP1 promotes hepatocellular carcinoma progression through miR-1294/GRAMD1A pathway. *J. Cell. Mol. Med.* **25**, 3793–3802 (2021).
56. S. Anders, A. Reyes, W. Huber, Detecting differential usage of exons from RNA-seq data. *Genome Res.* **22**, 2008–2017 (2012).
57. J. Majka, P. M. J. Burgers, The PCNA–RFC families of DNA clamps and clamp loaders, in *Progress in Nucleic Acid Research and Molecular Biology* (Academic Press, 2004), vol. 78, pp. 227–260.
58. J. Tomida, Y. Masuda, H. Hiroaki, T. Ishikawa, I. Song, T. Tsurimoto, S. Tateishi, T. Shiomi, Y. Kamei, J. Kim, K. Kamiya, C. Vaziri, H. Ohmori, T. Todo, DNA damage-induced ubiquitylation of RFC2 subunit of replication factor C complex. *J. Biol. Chem.* **283**, 9071–9079 (2008).
59. R. M. Overmeer, A. M. Gourdin, A. Giglia-Mari, H. Kool, A. B. Houtsmuller, G. Siegal, M. I. Fousteri, L. H. F. Mullenders, W. Vermeulen, Replication factor C recruits DNA polymerase δ to sites of nucleotide excision repair but is not required for PCNA recruitment. *Mol. Cell. Biol.* **30**, 4828–4839 (2010).
60. M. Boivin, J. Deng, V. Pfister, E. Grandgirard, M. Oulad-Abdelghani, B. Morlet, F. Ruffenach, L. Negroni, P. Koebel, H. Jacob, F. Riet, A. A. Dijkstra, K. McFadden, W. A. Clayton, D. Hong, H. Miyahara, Y. Iwasaki, J. Sone, Z. Wang, N. Charlet-Berguerand, Translation of GGC repeat expansions into a toxic polyglycine protein in NIID defines a novel class of human genetic disorders: The polyG diseases. *Neuron* **109**, 1825–1835.e5 (2021).
61. A. J. Milnerwood, L. A. Raymond, Early synaptic pathophysiology in neurodegeneration: Insights from Huntington's disease. *Trends Neurosci.* **33**, 513–523 (2010).
62. A. S. Ravalia, J. Lau, J. C. Barron, S. L. M. Purchase, A. L. Southwell, M. R. Hayden, F. Nafar, M. P. Parsons, Super-resolution imaging reveals extrastriatal synaptic dysfunction in presymptomatic Huntington disease mice. *Neurobiol. Dis.* **152**, 105293 (2021).
63. L. T. Ghaffari, D. Trotti, A. R. Haeusler, B. K. Jensen, Breakdown of the central synapses in *C9orf72*-linked ALS/FTD. *Front. Mol. Neurosci.* **15**, 1005112 (2022).
64. P. A. Gelon, P. A. Dutchak, C. F. Sephton, Synaptic dysfunction in ALS and FTD: Anatomical and molecular changes provide insights into mechanisms of disease. *Front. Mol. Neurosci.* **15**, 1000183 (2022).
65. S. Meftah, J. Gan, Alzheimer's disease as a synaptopathy: Evidence for dysfunction of synapses during disease progression. *Front. Synaptic Neurosci.* **15**, 1129036 (2023).
66. R. Martínez-Serra, L. Alonso-Nanclares, K. Cho, K. P. Giese, Emerging insights into synapse dysregulation in Alzheimer's disease. *Brain Commun.* **4**, fcac083 (2022).
67. N. F. Schor, D. W. Bianchi, Neurodevelopmental clues to neurodegeneration. *Pediatr. Neurol.* **123**, 67–76 (2021).
68. E. Taoufik, G. Kouroupi, O. Zygogianni, R. Matsas, Synaptic dysfunction in neurodegenerative and neurodevelopmental diseases: An overview of induced pluripotent stem-cell-based disease models. *Open Biol.* **8**, 180138 (2018).
69. S. Pourtoy-Brasselet, A. Sciauvaud, M.-G. Boza-Moran, M. Cailleret, M. Jarrige, H. Polvèche, J. Polentes, E. Chevet, C. Martinat, M. Peschanski, L. Aubry, Human iPSC-derived neurons reveal early developmental alteration of neurite outgrowth in the late-occurring neurodegenerative Wolfram syndrome. *Am. J. Hum. Genet.* **108**, 2171–2185 (2021).
70. S. Pasteuning-Vuhman, R. de Jongh, A. Timmers, R. J. Pasterkamp, Towards advanced iPSC-based drug development for neurodegenerative disease. *Trends Mol. Med.* **27**, 263–279 (2021).
71. S. P. Mojica-Perez, K. Stokes, D. C. Jaklic, S. Jahagirdar, M. Uhler, J. M. Parent, W. Niu, Protocol for selecting single human pluripotent stem cells using a modified micropipetter. *STAR Protocols* **4**, 102629 (2023).
72. Y. Shi, P. Kirwan, F. J. Livesey, Directed differentiation of human pluripotent stem cells to cerebral cortex neurons and neural networks. *Nat. Protoc.* **7**, 1836–1846 (2012).
73. H. M. T. Choi, C. R. Calvert, N. Husain, D. Huss, J. C. Barsi, B. E. Deverman, R. C. Hunter, M. Kato, S. M. Lee, A. C. T. Abelin, A. Z. Rosenthal, O. S. Akbari, Y. Li, B. A. Hay, P. W. Sternberg, P. H. Patterson, E. H. Davidson, S. K. Mazmanian, D. A. Prober, M. V. D. Rijn, J. R. Leadbetter, D. K. Newman, C. Readhead, M. E. Bronner, B. Wold, R. Lansford, T. Sauka-Spengler, S. E. Fraser, N. A. Pierce, Mapping a multiplexed zoo of mRNA expression. *Development* **143**, 3632–3637 (2016).
74. H. M. T. Choi, J. Y. Chang, L. A. Trinh, J. E. Padilla, S. E. Fraser, N. A. Pierce, Programmable in situ amplification for multiplexed imaging of mRNA expression. *Nat. Biotechnol.* **28**, 1208–1212 (2010).
75. D. Huss, H. M. T. Choi, C. Readhead, S. E. Fraser, N. A. Pierce, R. Lansford, Combinatorial analysis of mRNA expression patterns in mouse embryos using hybridization chain reaction. *Cold Spring Harb. Protoc.* **2015**, 259–268 (2015).
76. M. R. Glineburg, Y. Zhang, A. Krans, E. M. Tank, S. J. Barmada, P. K. Todd, Enhanced detection of expanded repeat mRNA foci with hybridization chain reaction. *Acta Neuropathol. Commun.* **9**, 73 (2021).
77. S. Andrews, FastQC: A quality control tool for high throughput sequence data (2010). www.bioinformatics.babraham.ac.uk/projects/fastqc/.
78. S. Chen, Y. Zhou, Y. Chen, J. Gu, Fastp: An ultra-fast all-in-one FASTQ preprocessor, in *Bioinformatics* (Oxford Univ. Press, 2018), vol. 34, pp. i884–i890.
79. A. Dobin, C. A. Davis, F. Schlesinger, J. Drenkow, C. Zaleski, S. Jha, P. Batut, M. Chaisson, T. R. Gingeras, STAR: Ultrafast universal RNA-seq aligner. *Bioinformatics* **29**, 15–21 (2013).
80. Y. Liao, G. K. Smyth, W. Shi, FeatureCounts: An efficient general purpose program for assigning sequence reads to genomic features. *Bioinformatics* **30**, 923–930 (2014).
81. M. I. Love, W. Huber, S. Anders, Moderated estimation of fold change and dispersion for RNA-seq data with DESeq2. *Genome Biol.* **15**, 550 (2014).
82. S. Falcon, R. Gentleman, Using GOstats to test gene lists for GO term association. *Bioinformatics* **23**, 257–258 (2007).
83. T. Wu, E. Hu, S. Xu, M. Chen, P. Guo, Z. Dai, T. Feng, L. Zhou, W. Tang, L. Zhan, X. Fu, S. Liu, X. Bo, G., clusterProfiler 4.0: A universal enrichment tool for interpreting omics data. *Innovation* **2**, 100141 (2021).
84. K. Blighe, S. Rana, M. Lewis, EnhancedVolcano: Publication-ready volcano plots with enhanced colouring and labeling (2023); https://github.com/kevinblighe/EnhancedVolcano.
85. H. Wickham, ggplot2: Elegant graphics for data analysis (2016); https://ggplot2.tidyverse.org.
86. C. O. Wilke, ggridges: Ridgeline Plots in "ggplot2" (2022); https://wilkelab.org/ggridges/.
87. J. Cheng, F. Metge, C. Dieterich, Specific identification and quantification of circular RNAs from sequencing data. *Bioinformatics* **32**, 1094–1096 (2016).
88. J. T. Robinson, H. Thorvaldsdóttir, W. Winckler, M. Guttman, E. S. Lander, G. Getz, J. P. Mesirov, Integrative genomics viewer. *Nat. Biotechnol.* **29**, 24–26 (2011).

Acknowledgments: We wish to thank the patients and their families for the donation of fibroblast and postmortem tissue samples for this study. We also thank the University of Michigan Human Stem Cell and Genome Editing Core, J. Parent, and S. Mojica-Perez for continuous support in generating CRISPR-corrected iPSC clones and for the use of their Incucyte S3 instrument. **Funding:** This work was supported by National Institute of Neurological Disorders and Stroke R21NS129096-01 (P.K.T.), National Institute of Neurological Disorders and Stroke R01NS086810-09 (P.K.T.), National Institute of Neurological Disorders and Stroke R01NS097542 (S.J.B.), National Institute of Neurological Disorders and Stroke R01NS113943 (S.J.B.), National Institute of Neurological Disorders and Stroke R56NS128110 (S.J.B.), National Institute of Child Health and Human Development P50HD104463-04 (P.K.T.), Veterans' Health Administration BLRD BX004842 (P.K.T. and A.K.), NIH Postbaccalaureate Research Education Program PREP, PAR-22-220 R25 (Y.P. and A.S.), the UM Medical Scientist Training Program (MSTP) (J.M.), the Silva Ataxia Foundation (V.K.), the Medical Research Council (MR/T001712/1) (A.C.), Fondazione Cariplo (grant no. 2019-1836) (A.C.), the Fragile X Association of Michigan (P.K.T.), the Wisne Foundation (P.K.T.), and the A. Alfred Taubman Medical Research Institute (P.K.T.). **Author contributions:** Conceptualization: C.J.M. and P.K.T. Methodology: C.J.M., A.K., S.J.G., Y.P., J.M., A.S., K.V.D., C.M., and A.P.B. Investigation: C.J.M., A.K., Y.P., J.M., A.S., M.A., S.W., A.C., and A.A.D. Formal analysis: C.J.M., A.K., and S.J.G.; Software: K.V.D., C.M., A.P.B., and S.J.B. Resources: C.J.M., A.C., A.N., A.K.,V.K., A.A.D., S.J.B., and P.K.T. Validation: A.K. and C.M. Funding acquisition: P.K.T. and S.J.B. Supervision: P.K.T. Visualization: C.J.M. Writing—original draft: C.J.M. and P.K.T. Writing—review and editing: C.J.M., A.K., S.J.G., Y.P., J.M., A.S., M.A., S.W., K.V.D., C.M., A.P.B., A.C., A.N., V.K., S.J.B., A.A.D., and P.K.T. **Competing**

interests: No commercial forces had editorial or supervisory input on the content of the manuscript or its figures. P.K.T. holds a shared patent on ASOs with Ionis Pharmaceuticals. He has served as a consultant with Denali Therapeutics, and he has licensed technology and antibodies to Denali and Abcam. V.K. is a cofounder of and senior advisor to DaCapo Brainscience and Yumanity Therapeutics, companies focused on CNS diseases. S.J.B. serves on the advisory board for Neurocures Inc., Symbiosis, Eikonizo Therapeutics, Ninesquare Therapeutics, the Live Like Lou Foundation, and the Robert Packard Center for ALS Research. S.J.B. has received research funding from Denali Therapeutics, Biogen Inc., Lysoway Therapeutics, Amylyx Therapeutics, Acelot Therapeutics, Meira GTX Inc., Prevail Therapeutics, Eikonizo Therapeutics, and Ninesquare Therapeutics. All other authors declare that they have no competing interests. **Data and materials availability:** All data needed to evaluate the conclusions in the paper are present in the paper and/or the Supplementary Materials. RNA-seq data are available at GEO: no. GSE248345.

Submitted 29 November 2023
Accepted 30 July 2024
Published 4 September 2024
10.1126/sciadv.adn2321
