# Supplementary material for: AAGGG repeat expansions trigger RFC1-independent synaptic dysregulation in human CANVAS neurons
Source: Sci Adv. 2024 Sep 4;10(36):eadn2321. doi: 10.1126/sciadv.adn2321 (PMC11373605; doi:10.1126/sciadv.adn2321)

ERRATUM PUBLISHED 2 OCTOBER 2026; SEE BELOW

Science Advances
AAAS

Supplementary Materials for

AAGGG repeat expansions trigger *RFC1*-independent synaptic dysregulation in human CANVAS neurons

Connor J. Maltby *et al.*

Corresponding author: Peter K. Todd, petertod@med.umich.edu

Sci. Adv. **10**, eadn2321 (2024)
DOI: 10.1126/sciadv.adn2321

The PDF file includes:

Figs. S1 to S15
Tables S1 to S4
Legends for movies S1 to S3

Other Supplementary Material for this manuscript includes the following:

Movies S1 to S3

Erratum (2 October 2026): In the original version of the article, there was an issue with the heterozygous control data file that was originally plotted in the graph of Fig. 6A, and its position on the PCA plot was incorrect. The batch effect of the selected RNA-seq run introduced a significant variance into the data related to the sample set in Fig. 6A. Attempts at batch correction were unsuccessful. Figure 6A has been replaced with the corrected version, with the heterozygous control sample data removed, and the PCA plot re-graphed without the heterozygous sample. Minor edits have been made to the main text to reflect these changes. As this sample was also included in fig. S12, the Supplementary Materials file has been updated with a corrected version of fig. S12 to remove this heterozygous control data.

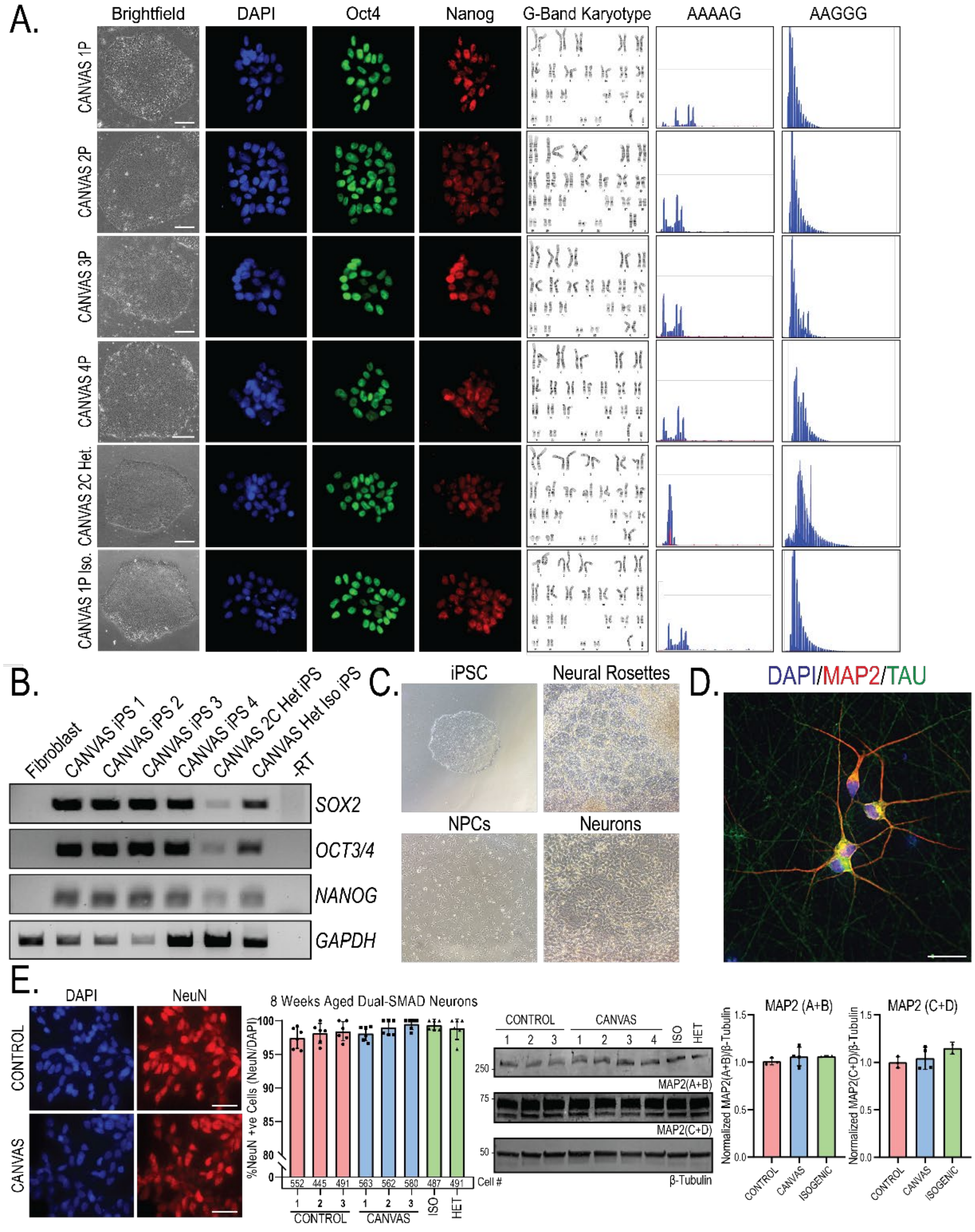

Fig. S1. Generation, characterization, and differentiation of CANVAS patient iPSC-derived neurons. A) G-Band Karyotype analysis (WiCel), Immunocytochemistry of CANVAS patient iPSCs for the pluripotency markers OCT4 and NANOG, scale = 25 µm, and repeat primed PCR for AAAAG/AAGGG expansions in all patient-derived iPSC lines. B) RT-PCR of CANVAS patient derived iPSCs for the pluripotency mRNA markers *SOX2*, *OCT3/4*, and *NANOG*. C) Brightfield images of the stages of neuronal differentiation from patient-derived iPSCs, showing iPSCs, neural rosettes, neural progenitor cells, mass-differentiated neuronal cells and re-plated neuronal cells for experimentation. D) Immunocytochemistry of a representative patient iPSC-derived neurons stained for the neuronal markers Tau and MAP2. Scale = 25 µm. E) Immunocytochemistry and protein analysis from 6-weeks aged iPSC-derived neurons analyzing neuronal markers (NeuN) vs DAPI and MAP2 vs β-Tubulin respectively to quantify efficiency of differentiation to neuronal cell populations across cell lines.

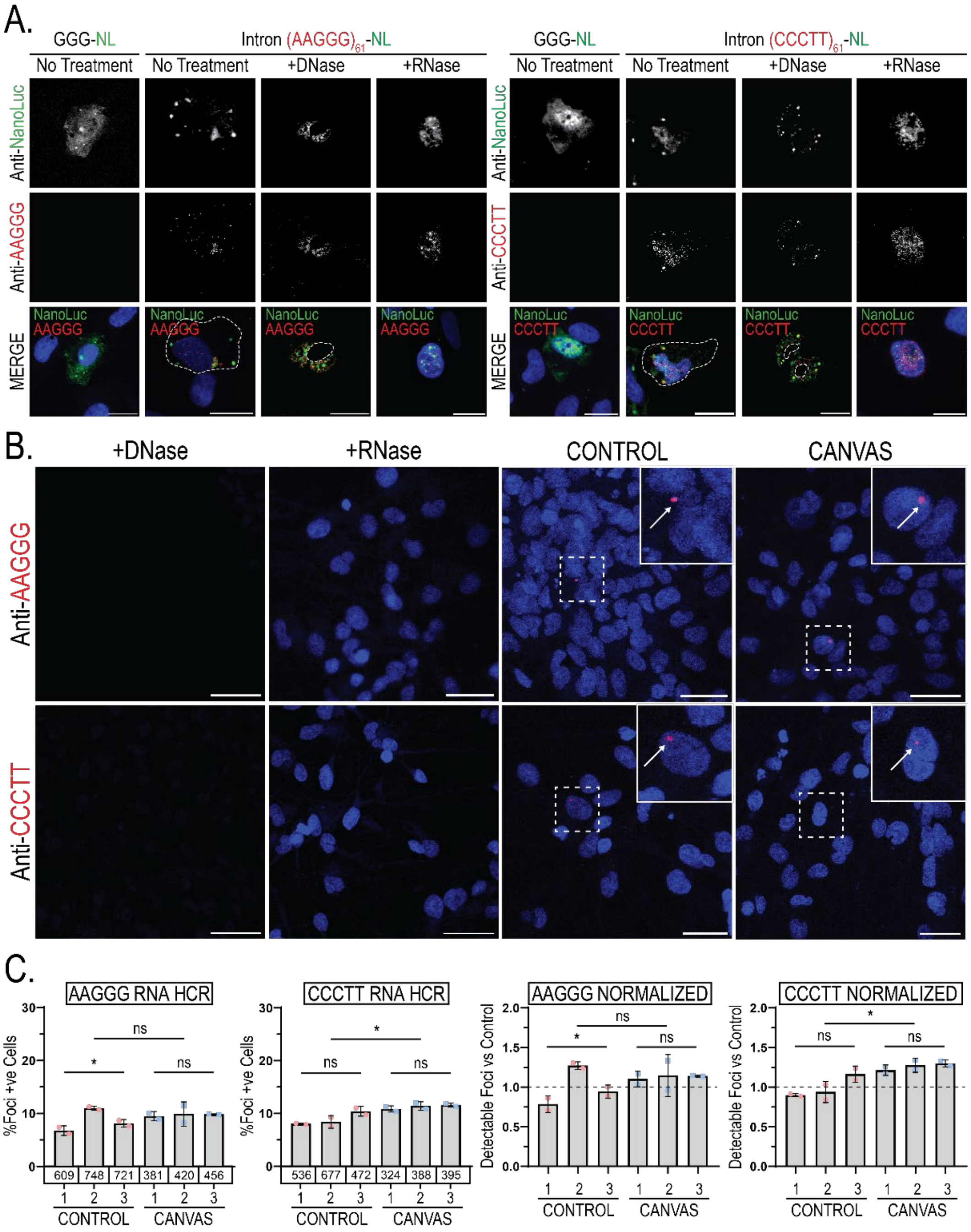

Fig. S2. RNA HCR probe validation and analysis of RNA foci formation in CANVAS patient and control iPSC-derived neurons. A) Confocal images of HEK293 cells transfected with control GGG-NL plasmid or plasmid expressing intronic AAGGG or CCCTT expansion followed by RNA HCR-FISH for AAGGG and CCCTT RNAs. Cells were fixed and treated with either anti-Nanoluc, anti-AAGGG, or anti-CCCTT fluorescent probes after no treatment, DNase, or RNase treatment to assess probe specificity. Scale = 10 µm. B) Confocal Images of representative CANVAS and control patient iPSC-derived neurons after RNA HCR-FISH utilizing either anti-AAGGG or anti-CCCTT fluorescent probes to assess sense or antisense RNA foci formation. Scale = 10 µm. C) Quantification of foci positive neurons for control (n=3) and CANVAS (n=3) patient iPSC-derived neurons with total n-numbers of neuronal cells analyzed per cell line indicated. AAGGG ($F(5, 12) = 3.619$, $P=0.074$), CCCTT ($F(5, 12) = 8.293$, $P=0.011$). Data were analyzed by one-way ANOVA with Sidak's post-hoc multiple comparison tests. Error = SD.

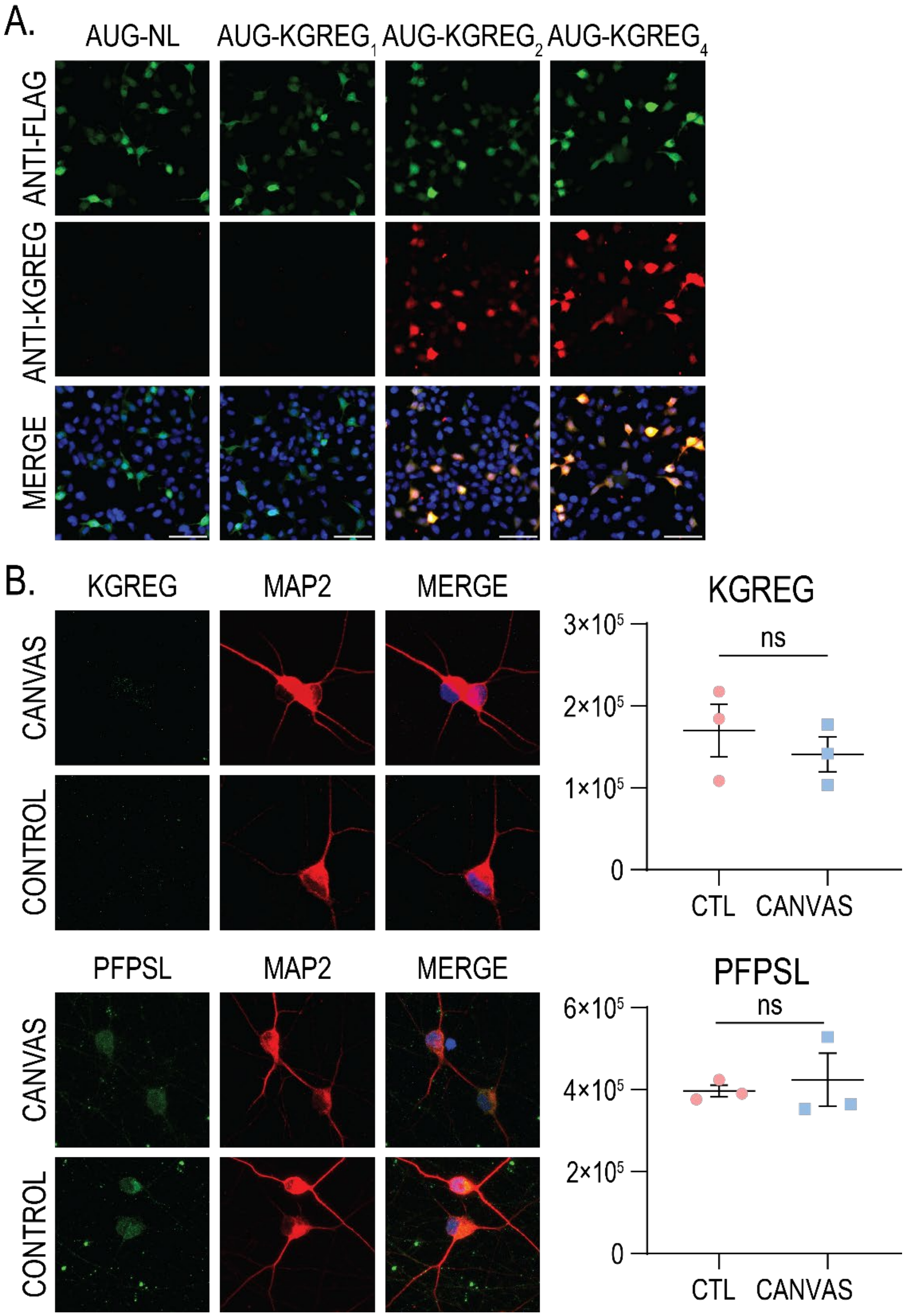

Fig. S3. Analysis of repeat-derived peptides using KGREG & PFPSL antibodies. A) ICC of HEK293 cells transfected with control plasmid or plasmids expressing 1x, 2x, and 4x KGREG FLAG-tagged plasmids using anti-FLAG M2 (1:100) and anti-KGREG (1:100) antibodies, scale = 25 µm. B) Immunocytochemistry and quantifications of 8-weeks aged control (n=3) and CANVAS (n=3) iPSC-derived neurons for sense AAGGG-derived KGREG peptides and antisense CCCTT-derived PFPSL peptides.

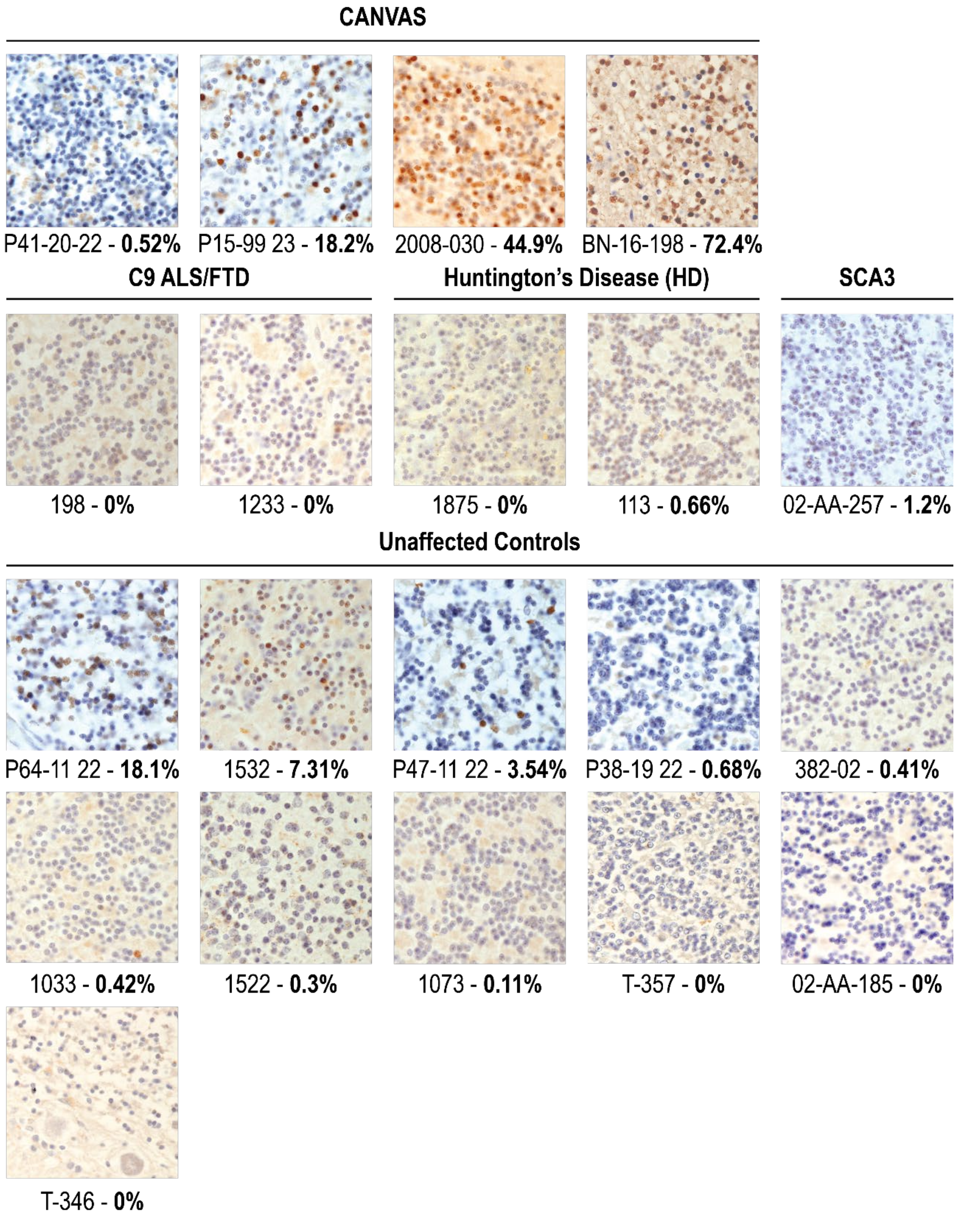

Fig. S4. **Supplementary IHC cerebellar staining of control and CANVAS brains for sense-derived AAGGG peptides.** 60x magnification immunohistochemistry images of control (n=16, including disease controls from cases of Spinocerebellar Ataxia Type 3 (n=1), C9orf72 associated FTD/ALS (n=2), Huntington's disease (n=2), and 11 non-disease controls) and RFC1 expansion CANVAS (n=4) patient post-mortem cerebellar vermis tissue stained with antisense anti-KGREG antibodies (1:100, acid AR).

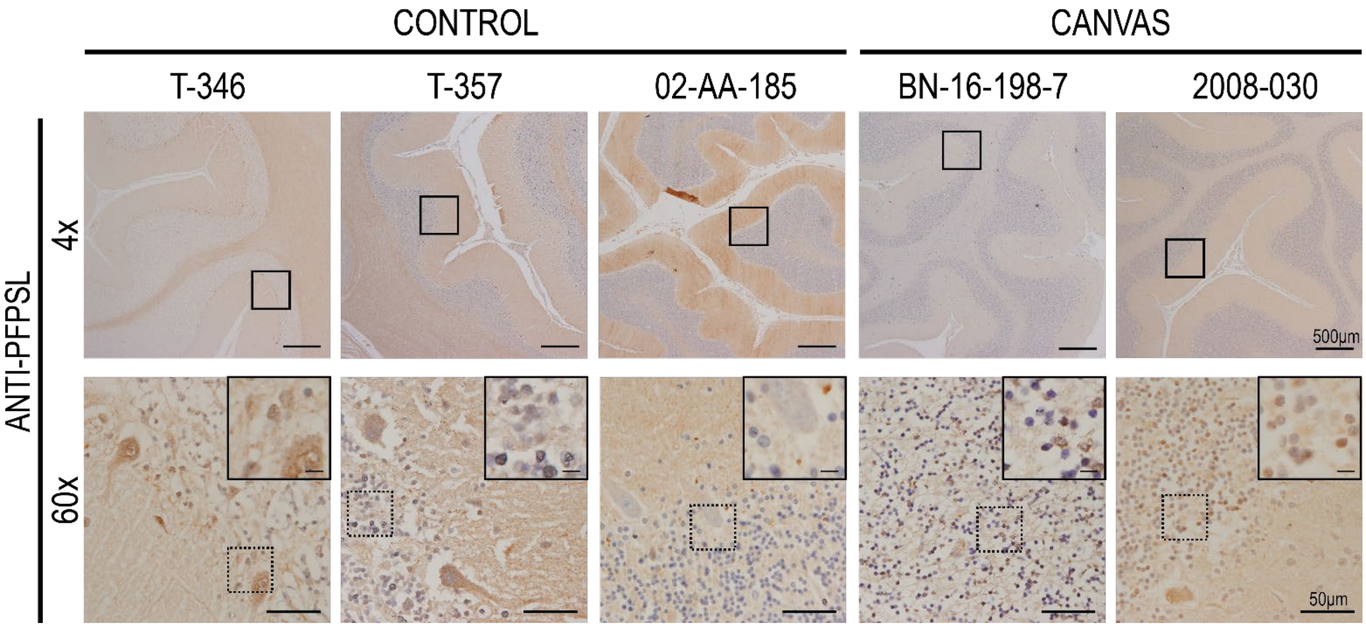

Fig. S5. Supplementary IHC cerebellar staining of control and CANVAS brains for antisense-derived PFPSL peptides. Immunohistochemistry of control (n=3) and RFC1 expansion CANVAS (n=2) patient post-mortem cerebellar vermis tissue stained with antisense anti-PFPSL antibodies (1:100, acid AR). Scale = 500 µm (4x), 50 µm (60x) and 20 µm (inset).

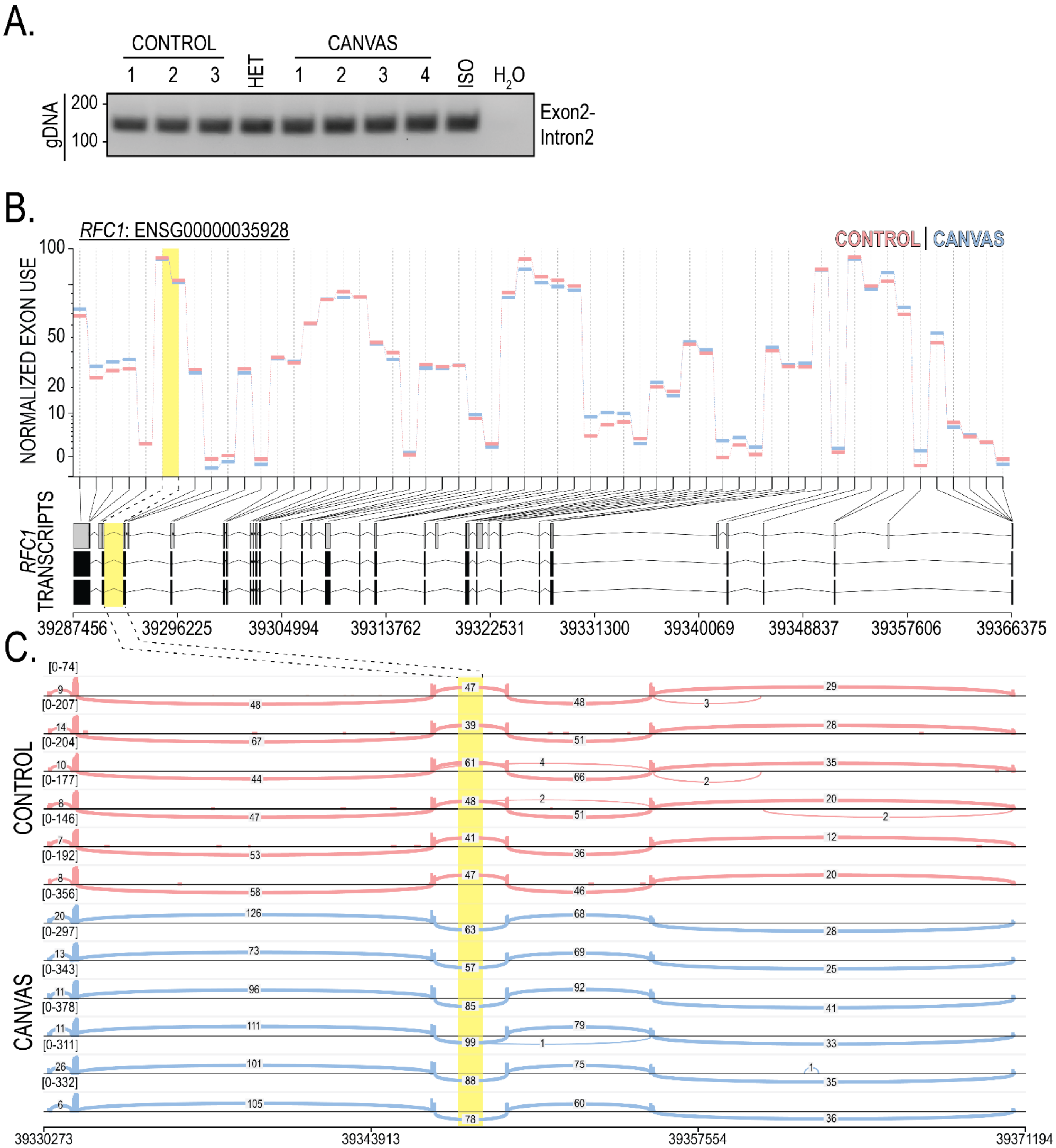

Fig. S6. Analysis of *RFC1* isoform and alternative exon usage RNA short-read sequencing. A) Endpoint RT-PCR utilizing primer sets spanning *RFC1* exon2-intron2 with gDNA extracted from control (n=2), CANVAS (n=4), Heterozygous (n=1), and Heterozygous Isogenic (n=1) iPSCs as a positive control for primer set efficiency. B) Normalized exon usage for *RFC1* mRNA transcripts in CANVAS patient iPSC-derived neurons, analyzed by DEXSeq of paired-end RNASeq reads. C) Sashimi plot of *RFC1* exons 1-5 in CANVAS patient iPSC-derived neurons indicating no skipping of exons flanking the intron 2 repeat expansion region (highlighted yellow).

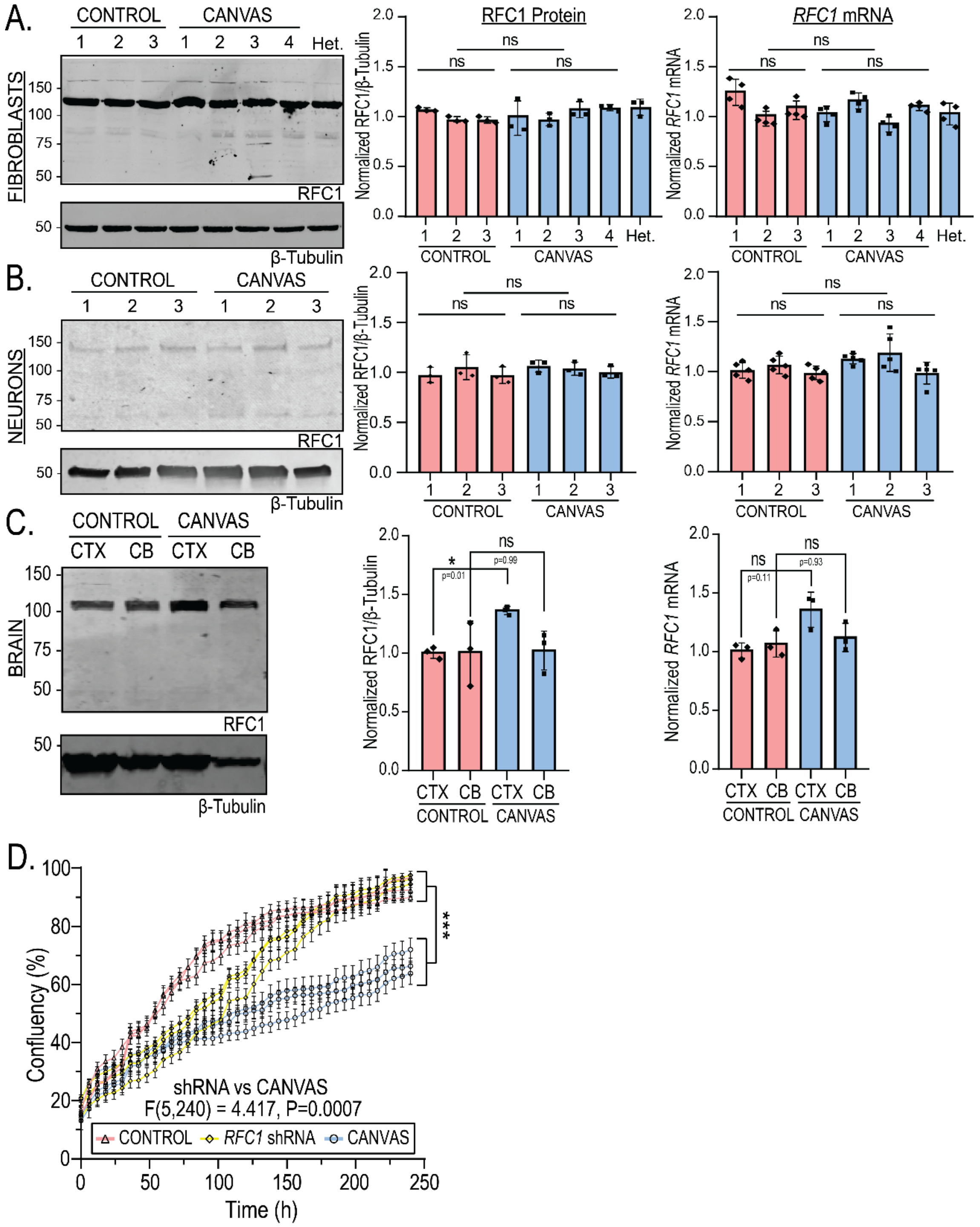

Fig. S7. RFC1 protein and mRNA expression is maintained in AAGGG expansion contexts. A) Analysis of RFC1 expression (left), quantification of normalized RFC1 expression (center, F(7,24) = 1.592, P=0.208), and quantification of normalized *RFC1* mRNA expression (right, F(7,24) = 4.944, P=0.0014) from CANVAS (n=4) and control (n=3) patient-derived fibroblasts. B) Analysis of RFC1 expression (left), quantification of normalized RFC1 expression (center, F(5,18) = 0.707, P=0.629)), and quantification of normalized RFC1 mRNA expression (right, F(5,24) = 3.029, P=0.029) from CANVAS and control (n=3) patient iPSC-derived neurons. C) Analysis of RFC1 protein (left), quantification of normalized RFC1 protein expression (center, F(3,8) = 2.010, P=0.191) from CANVAS (n=1) and control

(n=1) patient post-mortem brain tissue, and quantification of normalized RFC1 mRNA expression (right, $F(3,8) = 5.171$, $P=0.0281$). N = 3 biological replicates. Data were analyzed by one-way ANOVA with Sidak's post-hoc multiple comparison tests. Error = SD. D) 10-day time-course analysis of the rate of cellular division and proliferation in control fibroblasts (n=3) or CANVAS fibroblast lines (n=3) mock-treated or treated with RFC1 overexpression lentivirus ($F(5,240) = 2.358$, $P=0.245$). N = 3 biological replicates from 3 independent patient cell lines. Data were analyzed by one-way ANOVA with Sidak's post-hoc multiple comparison tests.

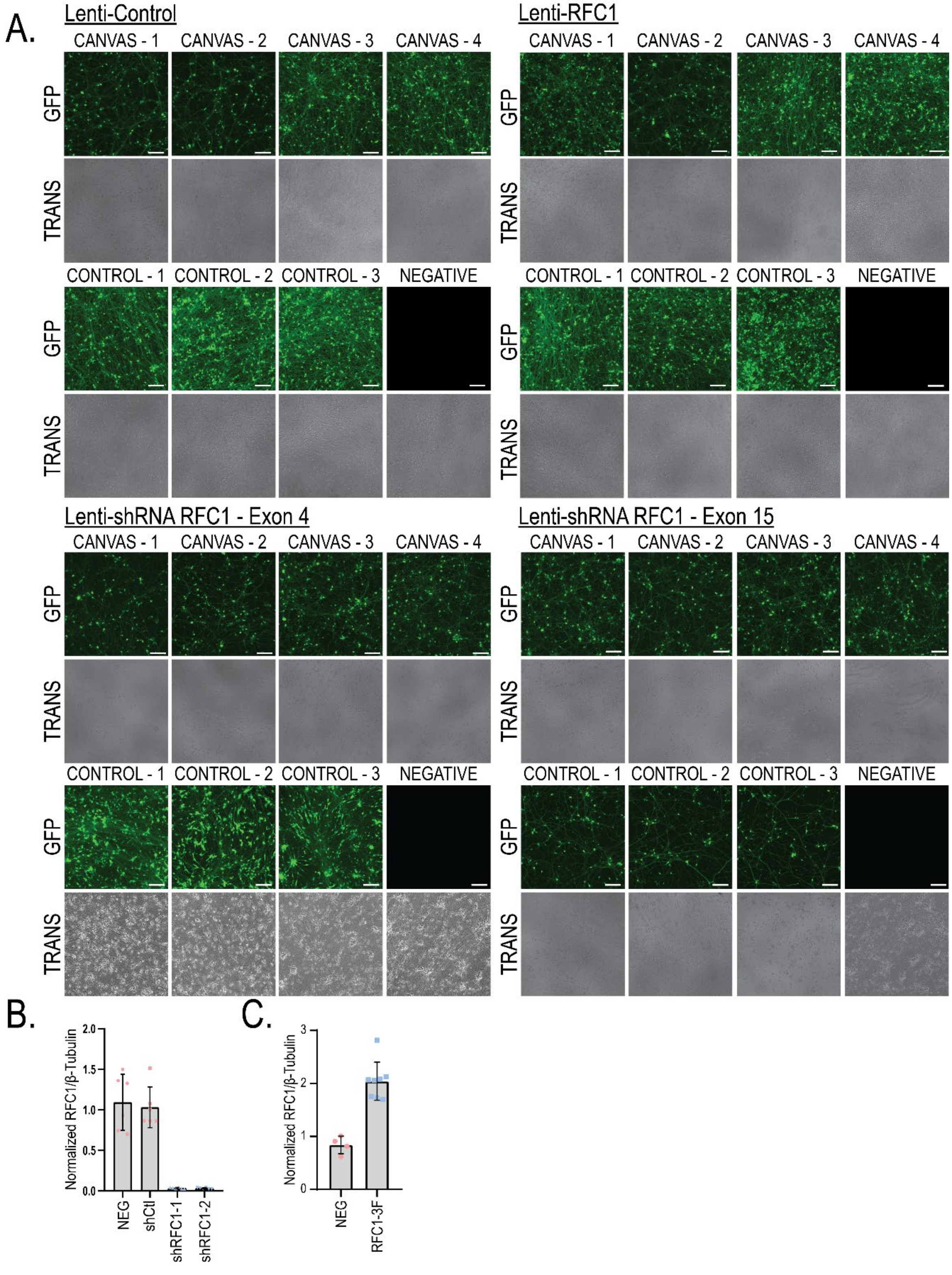

Fig. S8. Efficiency of lentiviral transduction in CANVAS patient and control iPSC-derived neurons. A) Brightfield and GFP fluorescence images of CANVAS (n=4) and control (n=3) patient iPSC-derived neurons transduced with control lentivirus, RFC1 overexpression lentivirus, or lentiviruses encoding shRNAs to knockdown RFC1 at exon4 or exon15. Scale = 50 µm. B-C) Quantification of immunoblots presented in main Fig. 7A & E upon shRNA RFC1 lentivirus treatment (B) or RFC1 overexpression lentivirus treatment (C) to assess for lentivirus efficiency.

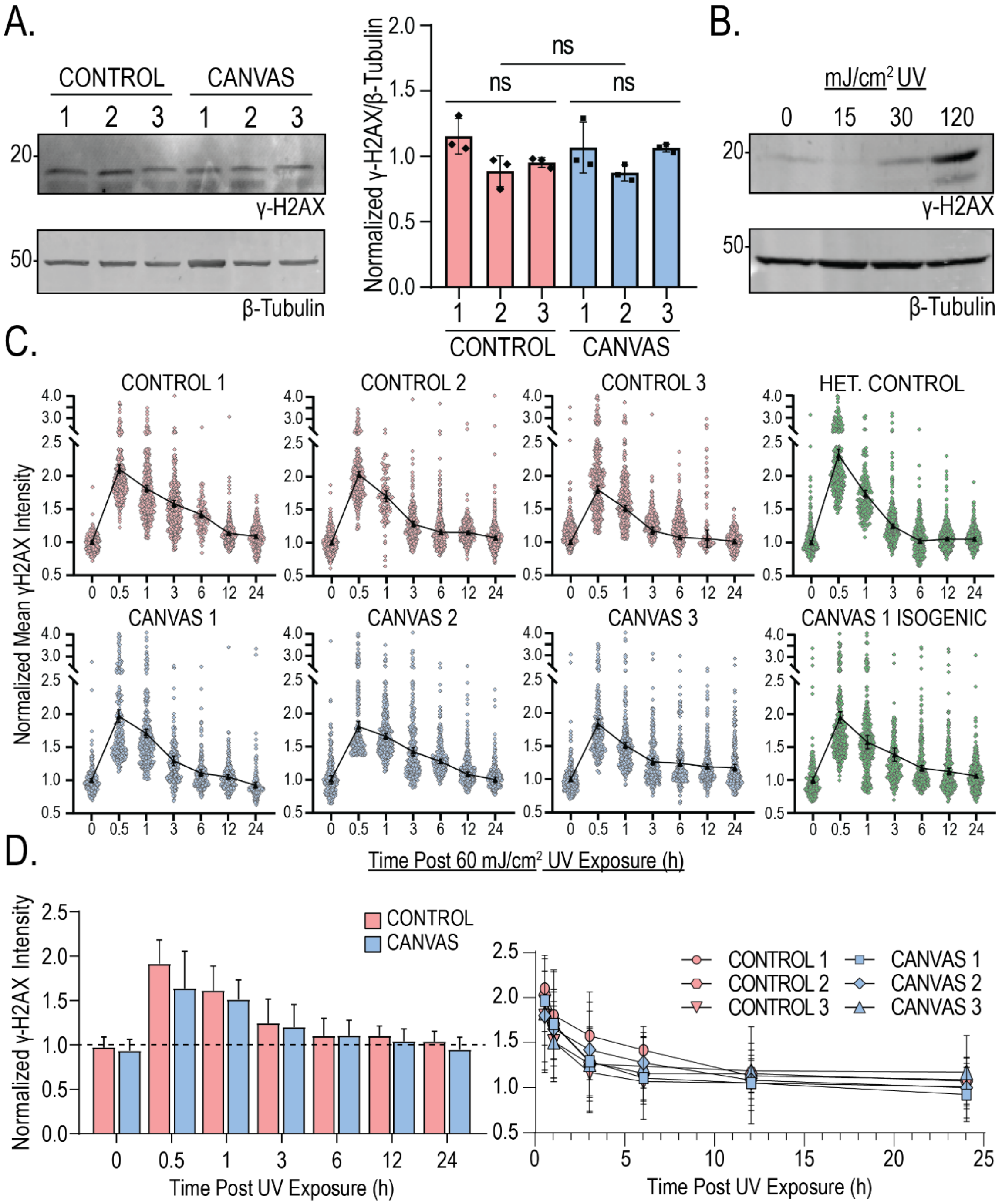

Fig. S9. Analysis of DNA damage accumulation and recovery in patient iPSC-derived neurons. A) Analysis of γ-H2AX expression (left), and quantification of normalized γ-H2AX levels (right, $F(5, 18) = 2.962$, $P=0.057$) from CANVAS (n=3) and control (n=3) patient iPSC-derived neurons. Data were analyzed by one-way ANOVA with Sidak's post-hoc multiple comparison tests. N = 3 biological replicates. B) Analysis of γ-H2AX levels in control iPSC-derived neurons exposed to 0, 15, 30, and 120 mJ/cm^2 UV irradiation. C) Quantification of mean γ-H2AX staining in control (n=3), CANVAS (n=3), heterozygous *RFC1* expansion (n=1), and CANVAS heterozygous isogenic corrected (n=1) patient iPSC-derived NeuN+ neuronal nuclei over a 24h period after 60 mJ/cm^2 UV exposure. D) Comparison of control (n=3) and CANVAS (n=3) γ-H2AX levels after 60 mJ/cm^2 UV exposure (left) and comparison of first derivative rates of γ-H2AX decline (right, $F(5, 30) = 0.033$, $P=0.999$). Error = SD.

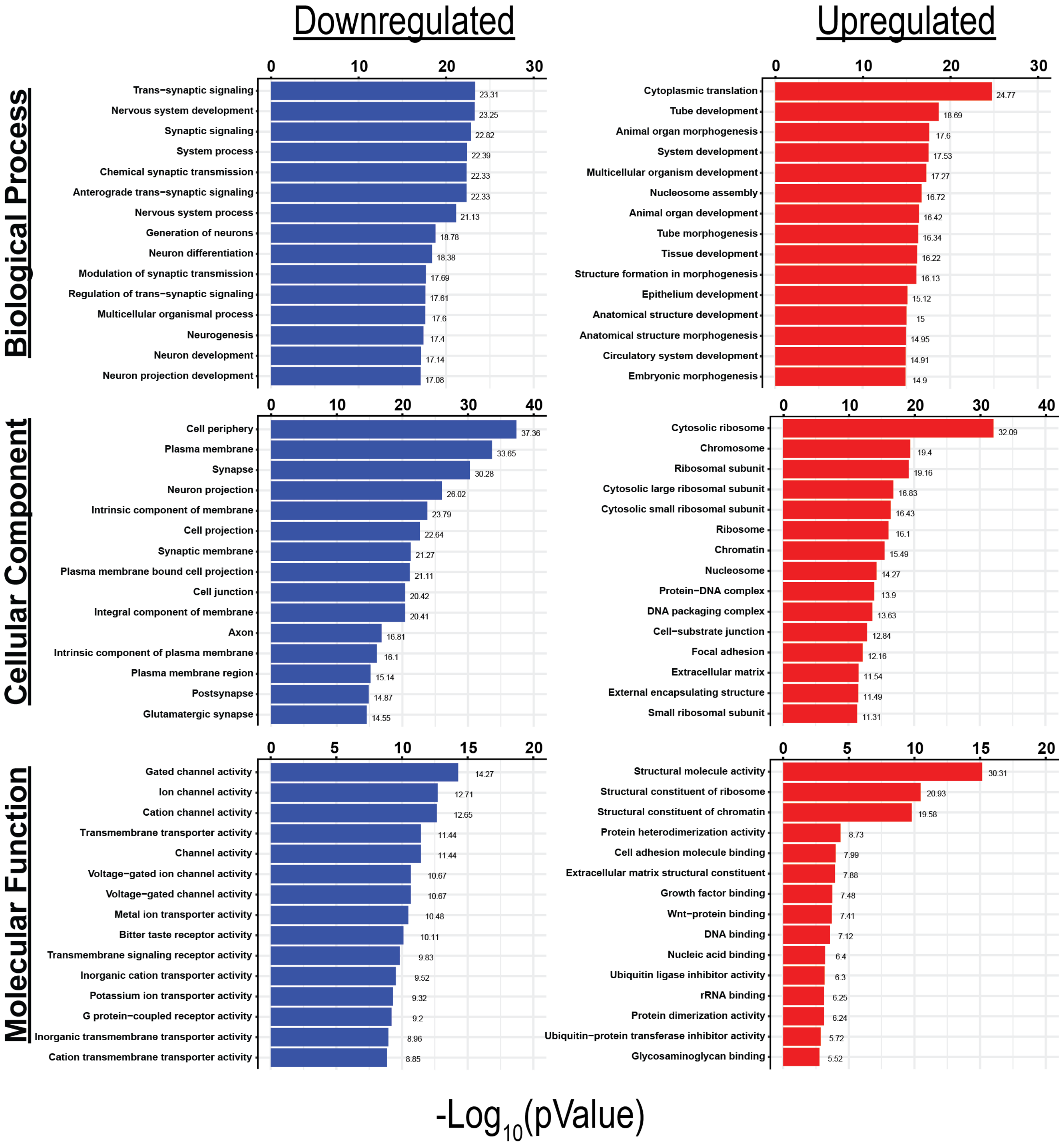

Fig. S10. Gene ontology (GO) analysis of dysregulated transcripts detected in CANVAS patient vs control iPSC-derived neurons. Full Gene Ontology (GO) pathway analysis of the top up and downregulated Biological Process, Cellular Component, and Molecular Functions in CANVAS patient vs control iPSC-derived neurons. N = 6 biological replicates from 3 individual CANVAS and control patients.

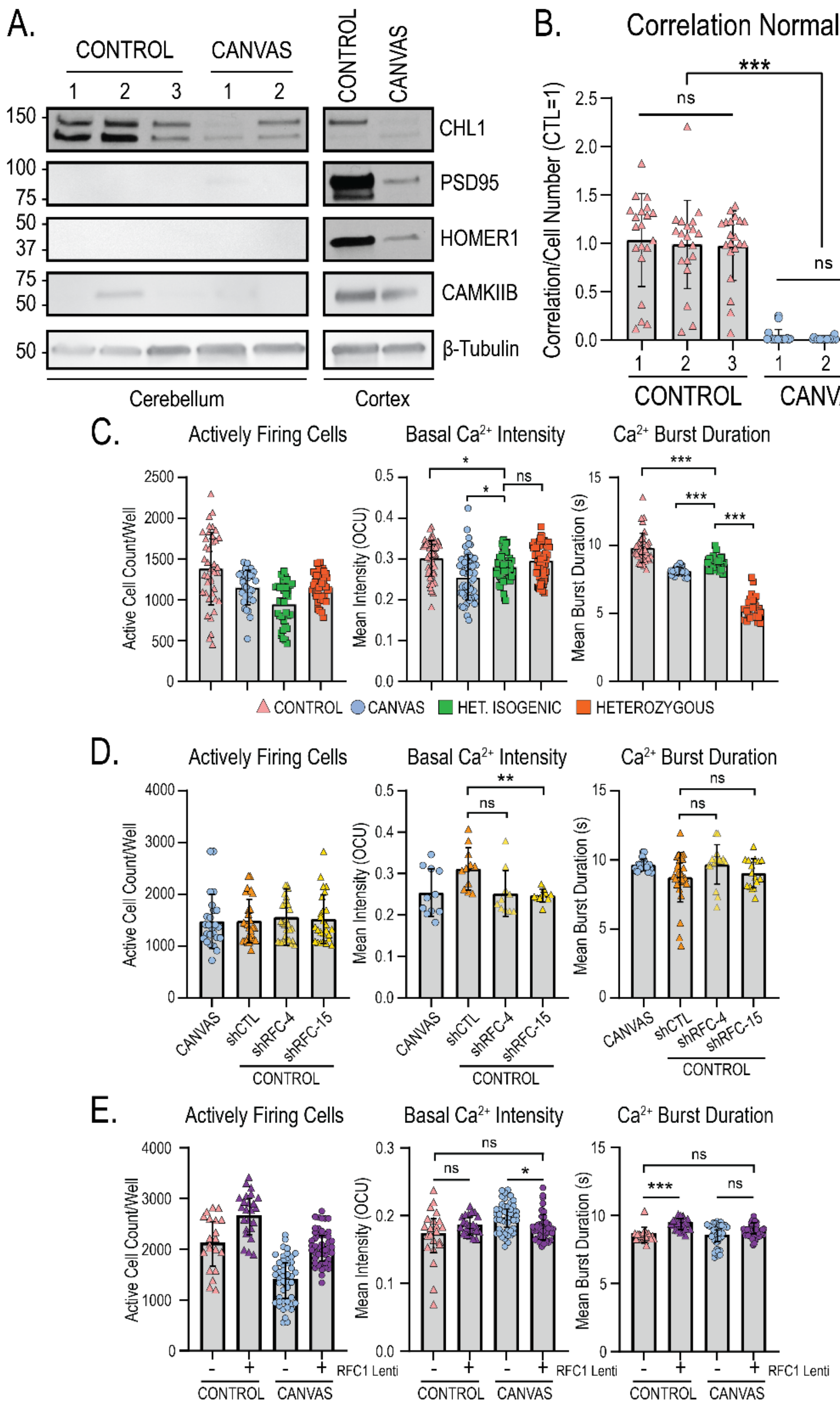

Fig. S11. Calcium imaging metrics from CANVAS patient and control iPSC-derived neurons. A) Protein expression analysis for selected synaptic proteins in cerebellar and cortical post-mortem tissue for available control and CANVAS patients. B) Firing correlation analysis of control (n=3) and CANVAS (n=3) patient iPSC-derived neurons normalized to the number of active cells ($F(5,114) = 59.89$, $P<0.0001$). C) Analysis of Ca^{2+} imaging metrics for control (n=3), CANVAS (n=3), Heterozygous (n=1) and Heterozygous Isogenic (n=1) patient iPSC-derived neurons. Basal Intensity ($F(2, 165) = 14.31$, $P<0.0001$), Burst Duration ($F(2, 101) = 48.79$, $P<0.0001$). D) Analysis of Ca^{2+} imaging metrics for CANVAS (n=3) and control (n=3) patient iPSC-derived neurons treated with shControl or shRFC1

exon4/exon15 lentiviruses. Basal Intensity ($F(3, 78) = 4.279$, $P=0.011$), Burst Duration ($F(3, 78) = 2.520$, $P=0.0641$). E) Analysis of Ca^{2+} imaging metrics for control (n=3) and CANVAS (n=3) patient iPSC-derived neurons treated with control or RFC1-overexpression lentivirus. Basal Intensity ($F(3, 135) = 5.621$, $P=0.0012$), Burst Duration ($F(3, 135) = 14.42$, $P<0.0001$). Each data point represents the mean of ~1000-3000 active cells per well. Data were analyzed by one-way ANOVA with Sidak's post-hoc multiple comparison tests. N= 3 biological replicates, error = SD.

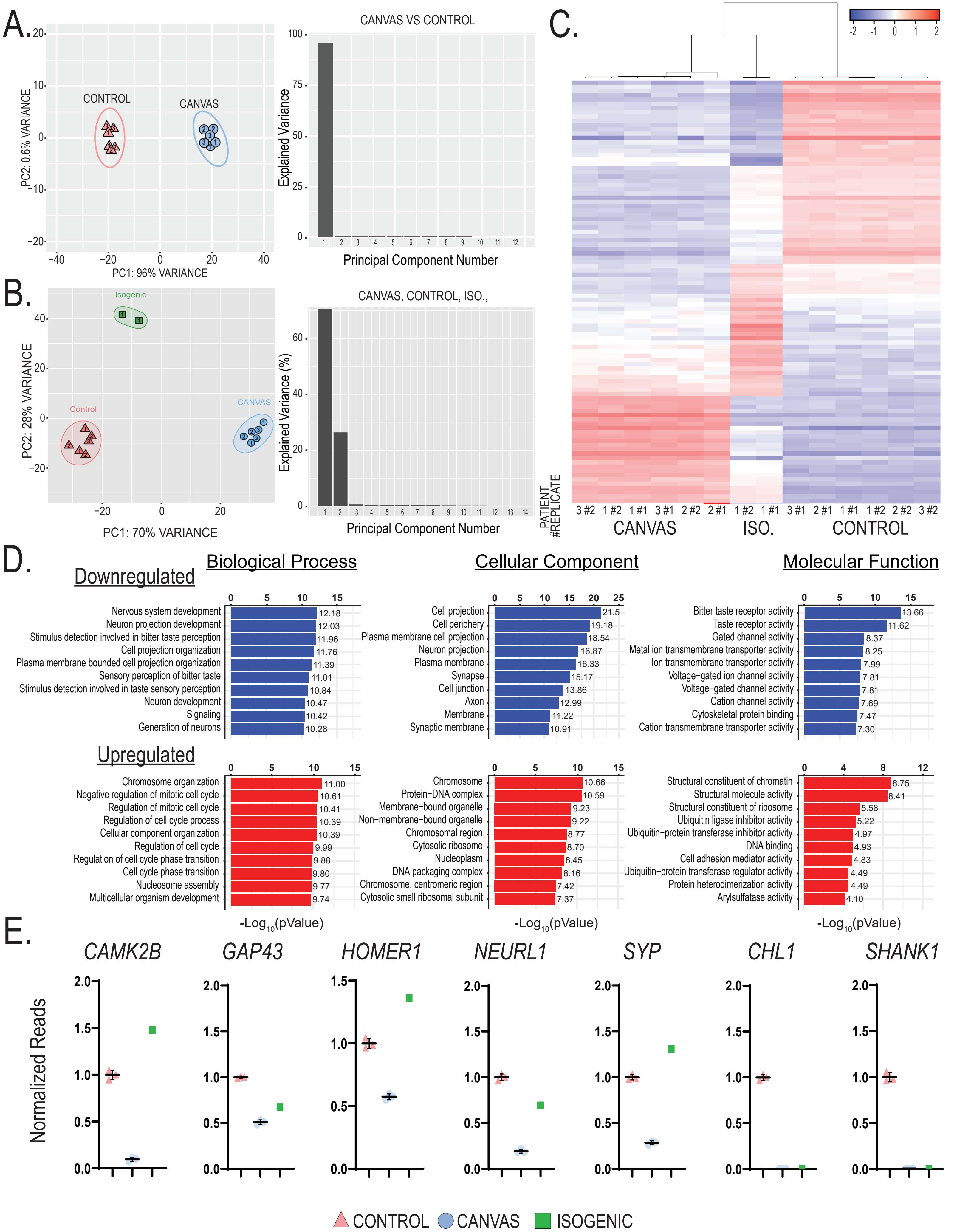
A.
CANVAS VS CONTROL
CONTROL
CANVAS
PC2: 0.6% VARIANCE
PC1: 96% VARIANCE
Explained Variance
Principal Component Number
B.
CANVAS, CONTROL, ISO.,
Isogenic
Control
CANVAS
PC2: 28% VARIANCE
PC1: 70% VARIANCE
Explained Variance (%)
Principal Component Number
C.
PATIENT #REPLICATE
3 #2 1 #2 1 #1 3 #1 2 #2 2 #1 1 #2 1 #1 3 #1 2 #1 1 #1 1 #2 2 #2 3 #2
CANVAS
ISO.
CONTROL
D.
Downregulated
Biological Process
Nervous system development 12.18
Neuron projection development 12.03
Stimulus detection involved in bitter taste perception 11.96
Cell projection organization 11.76
Plasma membrane bounded cell projection organization 11.39
Sensory perception of bitter taste 11.01
Stimulus detection involved in taste sensory perception 10.84
Neuron development 10.47
Signaling 10.42
Generation of neurons 10.28
Cellular Component
Cell projection 21.5
Cell periphery 19.18
Plasma membrane cell projection 18.54
Neuron projection 16.87
Plasma membrane 16.33
Synapse 15.17
Cell junction 13.86
Axon 12.99
Membrane 11.22
Synaptic membrane 10.91
Molecular Function
Bitter taste receptor activity 13.66
Taste receptor activity 11.62
Gated channel activity 8.37
Metal ion transmembrane transporter activity 8.25
Ion transmembrane transporter activity 7.99
Voltage-gated ion channel activity 7.81
Voltage-gated channel activity 7.81
Cation channel activity 7.69
Cytoskeletal protein binding 7.47
Cation transmembrane transporter activity 7.30
Upregulated
Chromosome organization 11.00
Negative regulation of mitotic cell cycle 10.61
Regulation of mitotic cell cycle 10.41
Regulation of cell cycle process 10.39
Cellular component organization 10.39
Regulation of cell cycle 9.99
Regulation of cell cycle phase transition 9.88
Cell cycle phase transition 9.80
Nucleosome assembly 9.77
Multicellular organism development 9.74
Chromosome 10.66
Protein-DNA complex 10.59
Membrane-bound organelle 9.23
Non-membrane-bound organelle 9.22
Chromosomal region 8.77
Cytosolic ribosome 8.70
Nucleoplasm 8.45
DNA packaging complex 8.16
Chromosome, centromeric region 7.42
Cytosolic small ribosomal subunit 7.37
Structural constituent of chromatin 8.75
Structural molecule activity 8.41
Structural constituent of ribosome 5.58
Ubiquitin ligase inhibitor activity 5.22
Ubiquitin-protein transferase inhibitor activity 4.97
DNA binding 4.93
Cell adhesion mediator activity 4.83
Ubiquitin-protein transferase regulator activity 4.49
Protein heterodimerization activity 4.49
Arylsulfatase activity 4.10
-Log10(pValue)
E.
CAMK2B
GAP43
HOMER1
NEURL1
SYP
CHL1
SHANK1
Normalized Reads
CONTROL
CANVAS
ISOGENIC

Fig. S12. Supplemental data of transcriptomic analyses from control, CANVAS, and heterozygous isogenic correction patient iPSC-derived neurons. A) Principal component analyses of CANVAS (n=3) vs control (n=3) patient iPSC-derived neurons (left) with scree plot of explained variance with principal component axis of variance per sample shown for PC1 and PC2 (right). B) CANVAS (n=3), control (n=3), and Heterozygous Isogenic (n=1) patient iPSC-derived neurons (left) with principal component axis of variance per sample shown for PC1 and PC2 (right). C) Heatmap of normalized expression for the top 1000 genes differentially expressed in CANVAS, control, and CANVAS isogenic patient iPSC-derived neurons. D) Gene Ontology (GO) pathway analysis of the top up/downregulated Biological Process, Cellular Component, and Molecular Function for the genes that showed significant expression correction in heterozygous isogenic iPSC-derived neurons compared to CANVAS/control. E) Normalized read counts for CAMK2B, GAP43, HOMER1, NEURL1, SYP, CHL1, and SHANK1 from control (n=6), CANVAS (n=6), and isogenic (n=2) iPSC-derived neurons

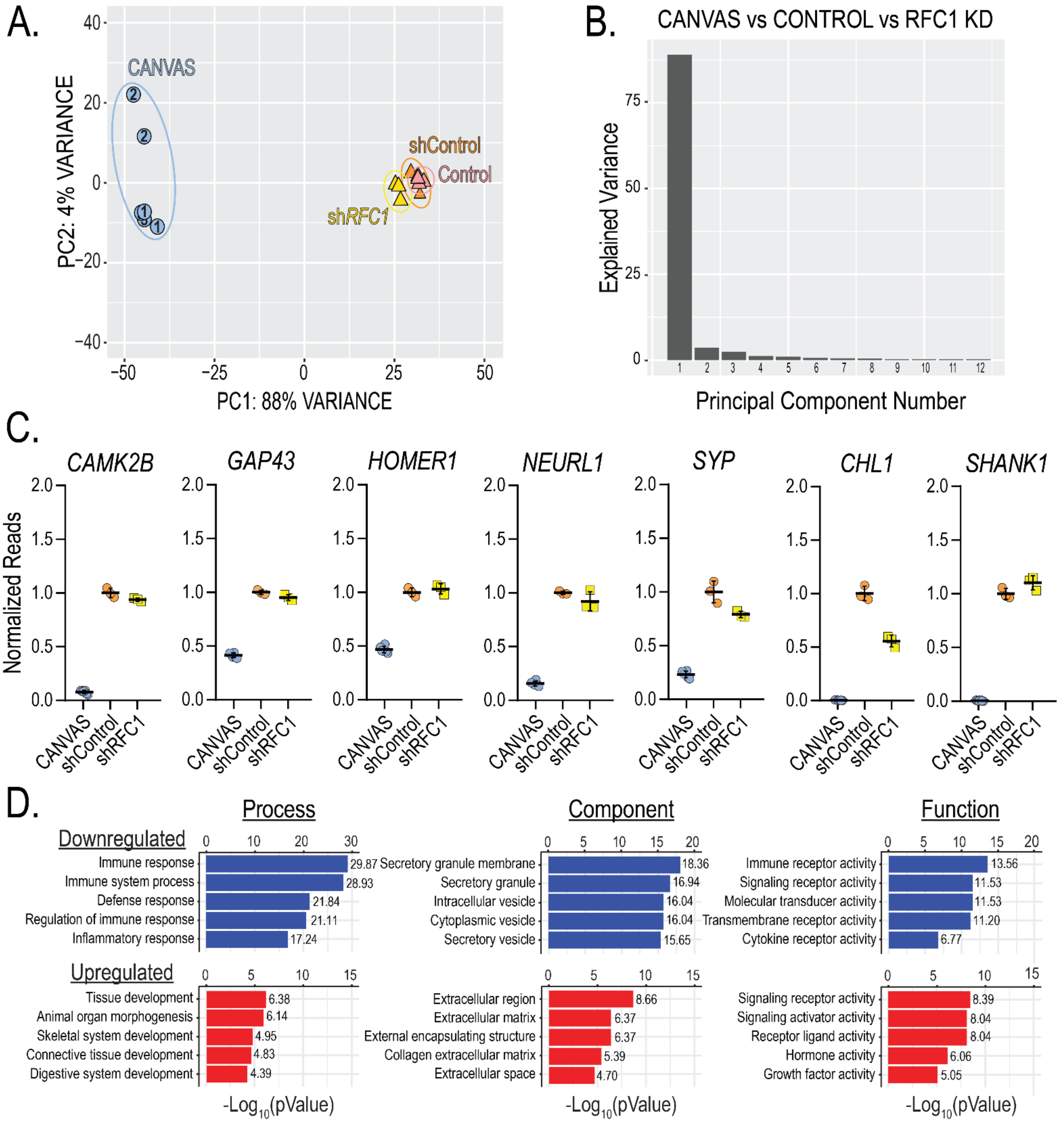

Fig. S13. Supplemental data to RFC1 knockdown RNASeq analyses. A) Principal component analyses of CANVAS (n=6), control (n=3), control *shControl* (n=3), and control *shRFC1* (n=3) patient iPSC-derived neurons. B) Scree plots of explained variance with principal component axis of variance per sample shown for PC1 and PC2. C) Normalized read counts for *CAMK2B, GAP43, HOMER1, NEURL1, SYP, CHL1*, and *SHANK1* from CANVAS (n=6), *shControl* (n=3), and *shRFC1* (n=3) iPSC-derived neurons. D) Gene Ontology (GO) pathway analysis of the top up/downregulated Biological Process, Cellular Component, and Molecular Function for the genes that showed significant expression correction in RFC1 knockdown iPSC-derived neurons compared to CANVAS/control.

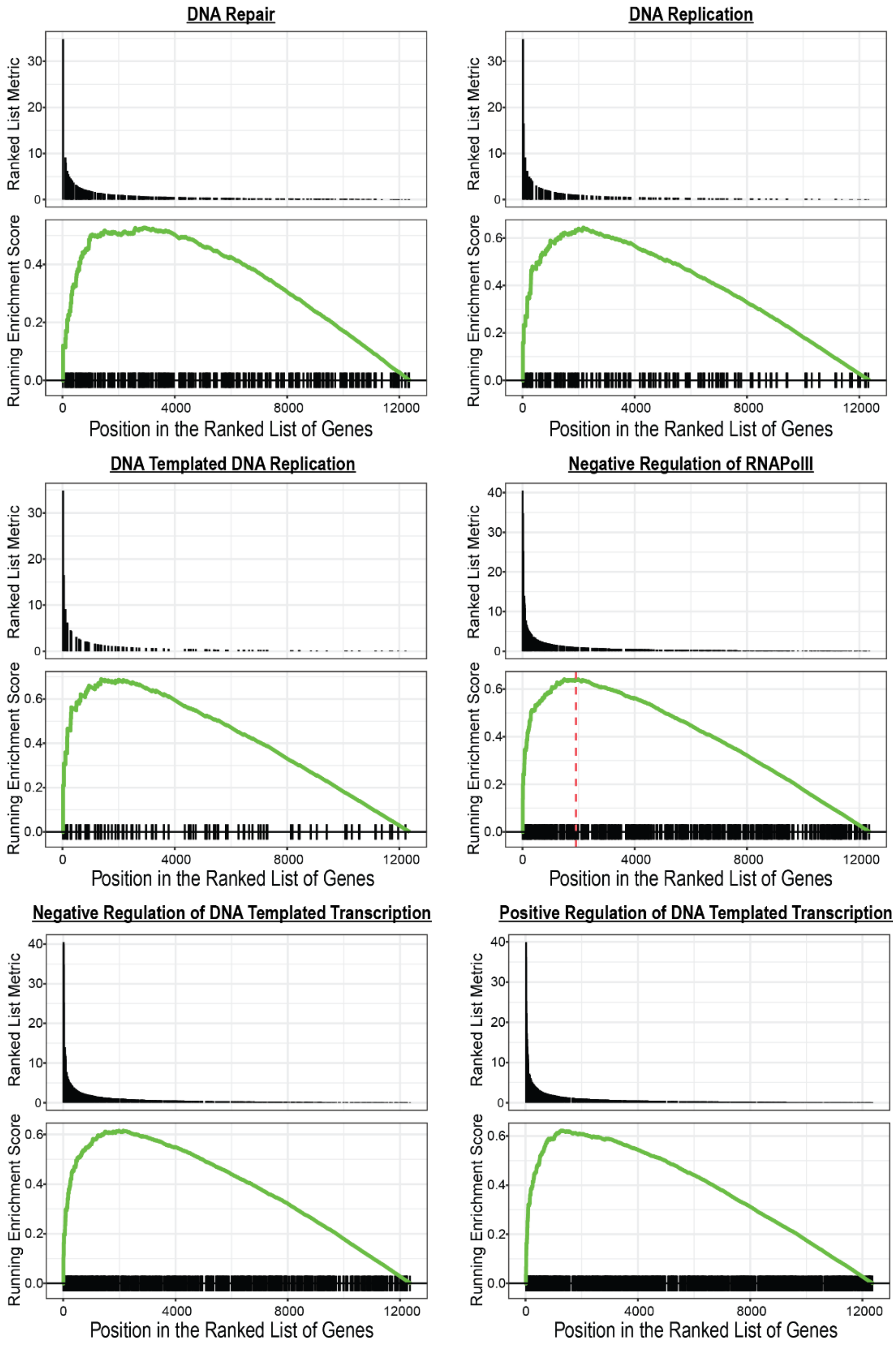

Fig. S14. Gene set enrichment analysis (GSEA) of RFC1-associated functions in RFC1 knockdown control iPSC-derived neurons. Gene set enrichment analysis (GSEA) of the RFC1-associated functions DNA repair, DNA replication, DNA templated DNA replication, negative regulation of RNA Pol II, and positive and negative regulation of DNA templated transcription in RFC1 knockdown control iPSC-derived neurons.

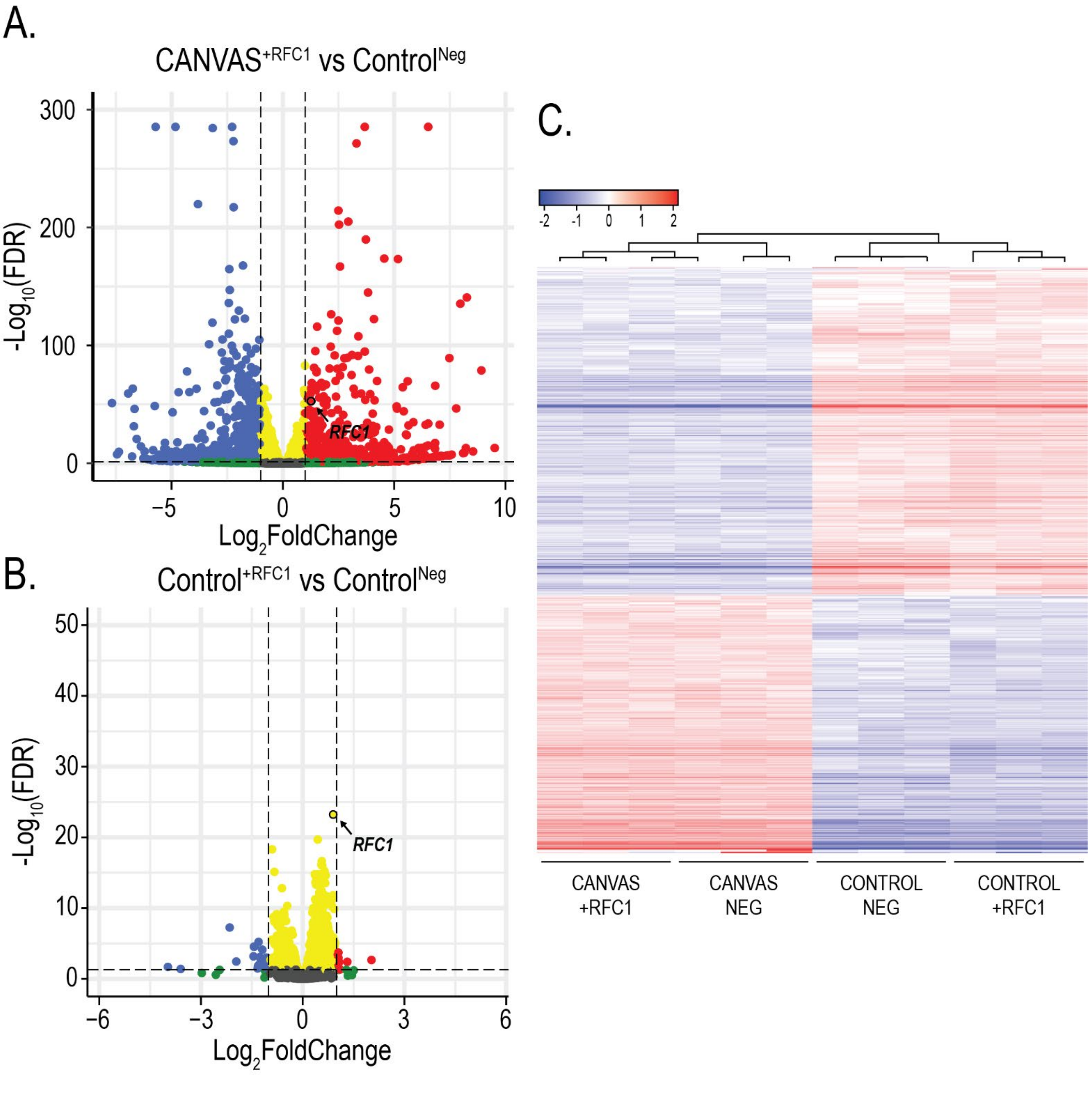

Fig. S15. RFC1 reprovision RNASeq analyses. A) Volcano plot of -Log_{10}FDR vs Log_2(Fold Change) for CANVAS patient-derived neurons transduced with either full-length *RFC1* CDS lentivirus or control lentivirus (n=3/group), RFC1 labelled. B) Volcano plot of -Log_{10}FDR vs Log_2(Fold Change) for control-derived neurons transduced with either full-length RFC1 CDS lentivirus or control lentivirus (n=3/group), RFC1 labelled. C) Heatmap of normalized expression for the top 1000 genes differentially expressed in CANVAS, control, and CANVAS isogenic patient iPSC-derived neurons.

Purpose	Primers	Reagents	Conditions
NHEJ CRISPR	Fwd gRNA: GAGAATAGCAACGGTGTAGCTGG<br>Rev gRNA: TCATTTTCTGAAATACGGACAGG	20uM HiFi Cas9 (IDT)<br>tracR-ATTO550 (IDT) 1:1 gRNA(total)<br>120ul R-Buffer	1450V, 10ms, 3-pulses
Non-Edited Screening Repeat-Flanking PCR	Fwd: CTGAAGTGATTGGCCTGTCTCCC<br>Rev: CACTGGATCAAGGACAGAGTCA	2X Faststart Master Mix (Roche)<br>Primers 0.5 M<br>gDNA 50 ng	95°C 4 min<br>[95°C 30s, 60°C 30s, 72°C 60s] x35<br>72 C 5 min
Edited Screening Repeat-Spanning PCR	Fwd: GGGTGGTGGCTGTCTCATC<br>Rev: CACTGGATCAAGGACAGAGTCA		
Repeat-Primed PCR	Fwd: FAM-TCAAGTGATACTCCAGCTACACCGT<br>Anchor: CAGGAAACAGCTATGACC<br>**(AAAAG)11 Allele**<br>Rev1: CAGGAAACAGCTATGACCAACAGAGCAAGACTCTGTTTCAAAAAAGAAAAGAAAAGAAAAGAAAA<br>Rev2: CAGGAAACAGCTATGACCAACAGAGCAAGACTCTGTTTCAAAAAGAAAAGAAAAGAAAAGAAAA<br>Rev3: CAGGAAACAGCTATGACCAACAGAGCAAGACTCTGTTTCAAAAGAAAAGAAAAGAAAAGAAAA<br>**(AAGGG)exp Allele**<br>Rev1: CAGGAAACAGCTATGACCAACAGAGCAAGACTCTGTTTCAAAAAAGGGAAGGGAAGGGAAGGGAA<br>Rev2: CAGGAAACAGCTATGACCAACAGAGCAAGACTCTGTTTCAAAAAGGGAAGGGAAGGGAAGGGAA<br>Rev3: CAGGAAACAGCTATGACCAACAGAGCAAGACTCTGTTTCAAAAGGGAAGGGAAGGGAAGGGAA	2X Phusion Flash High-Fidelity PCR Master Mix (ThermoFisher)<br>Fw Primer 0.5 M<br>Anchor 0.5 M<br>Rv primers (Rv1:Rv2:Rv3=1:1:1) 0.05 M<br>DMSO 3%<br>gDNA 50 ng	98°C 3 min<br>[98°C 10s, 65°C 15s, 72°C 60s] x35<br>72°C 5 min
Intron 2 Retention Screening	Exon 2 Fwd: CATTCGGAAATTCTTTGGAGTA<br>Exon 3 Rev: ATCCTCTTTACGGGAGCTATTTAC<br>Intron 2 Rev: TCAATGCAAAATTATACCCAGA	2X Faststart Master Mix (Roche)<br>Primers 0.5 M<br>gDNA 50 ng	95°C 4 min<br>[95°C 30s, 59°C 30s, 72°C 60s] x35<br>72 C 5 min
qRT-PCR	RFC1: FAM-MGB Taqman Probes (Thermo Hs01099126_m1, Exon14-15, Amplicon Length 70 bp)<br>Actin: VIC-MGB-PL Taqman Probes (Thermo Hs99999903_m1, Exon 1, Amplicon Length 171 bp)	TaqMan™ 2X Universal PCR Master Mix (ThermoFisher)<br>1X RFC1 FAM Taqman Probes<br>1X Actin VIC Taqman Probes<br>2 µl cDNA	95°C 20s<br>[95°C 5s, 60°C 20s] x40

Table S1.

Table of PCR primer sequences, reagents, and thermocycling conditions used.

**Anti-Nanoluc-3F**	
Nluc-3xF-DNAp50-B1DI-P1	GAGGAGGGCAGCAAACGGGAAGAGTCTTCCTTTACGATATTTTGTAGCCGGCTGTCTGTCGCCAGTCCCCAACGAAATCTTCGAGTGTGAAATATAGCATTCTTTCTTGAGGAGGGCAGCAAACGGGAAGAG
Nluc-3xF-DNAp50-B1DI-P2	GAGGAGGGCAGCAAACGGGAAGAGTCTTCCTTTACGATATTTTGGATCGGAGTTACGGACACCCCGAGATTCTGAAACAAACTGGACACACATATAGCATTCTTTCTTGAGGAGGGCAGCAAACGGGAAGAG
Nluc-3xF-DNAp50-B1DI-P3	GAGGAGGGCAGCAAACGGGAAGAGTCTTCCTTTACGATATTAAATTTTTTCGATCTGGCCCATTTGGTCGCCGCTCAGACCTTCATACGGGATATAGCATTCTTTCTTGAGGAGGGCAGCAAACGGGAAGAG
Nluc-3xF-DNAp50-B1DI-P4	GAGGAGGGCAGCAAACGGGAAGAGTCTTCCTTTACGATATTCGTAACCCCGTCGATTACCAGTGTGCCATAGTGCAGGATCACCTTAAAGTATATAGCATTCTTTCTTGAGGAGGGCAGCAAACGGGAAGAG

**Anti-CCCTT 5' Anchor**	
CCCTT-P1	GAGGAGGGCAGCAAACGGGAAGAGTCTTCCTTTACGATATTAAGGGAAGGGAAGGGAAGGGAAGGGAAGGGAAGGGAAGGGAGCATGTTCTAAAGAGAATATAGCATTCTTTCTTGAGGAGGGCAGCAAACGGGAAGAG

**Anti-CTTTT 5' Anchor**	
CTTTT-P1	GAGGAGGGCAGCAAACGGGAAGAGTCTTCCTTTACGATATTAAAAGAAAAGAAAAGAAAAGAAAAGAAAAGAAAAGAAAAGAGCATGTTCTAAAGAGAATATAGCATTCTTTCTTGAGGAGGGCAGCAAACGGGAAGAG

**Anti-AAGGG 5' Anchor**	
AAGGG-P1	GAGGAGGGCAGCAAACGGGAAGAGTCTTCCTTTACGATATTCCCTTCCCTTCCCTTCCCTTCCCTTCCCTTCCCTTCCCTTTTTTGAAACAGAGTCATATAGCATTCTTTCTTGAGGAGGGCAGCAAACGGGAAGAG
AAGGG-P2	GAGGAGGGCAGCAAACGGGAAGAGTCTTCCTTTACGATATTCCCTTCCCTTCCCTTCCCTTCCCTTCCCTTCCCTTCCCTTTTTGAAACAGAGTC ATATAGCATTCTTTCTTGAGGAGGGCAGCAAACGGGAAGAG
AAGGG-P3	GAGGAGGGCAGCAAACGGGAAGAGTCTTCCTTTACGATATTCCCTTCCCTTCCCTTCCCTTCCCTTCCCTTCCCTTCCCTTTTGAAACAGAGTC ATATAGCATTCTTTCTTGAGGAGGGCAGCAAACGGGAAGAG

**Anti-AAAAG 5' Anchor**	
AAAAG-P1	GAGGAGGGCAGCAAACGGGAAGAGTCTTCCTTTACGATATTCTTTTCTTTTCTTTTCTTTTCTTTTCTTTTCTTTTCTTTTTTTTGAAACAGAGTCATATAGCATTCTTTCTTGAGGAGGGCAGCAAACGGGAAGAG
AAAAG-P2	GAGGAGGGCAGCAAACGGGAAGAGTCTTCCTTTACGATATTCTTTTCTTTTCTTTTCTTTTCTTTTCTTTTCTTTTCTTTTTTTGAAACAGAGTCATATAGCATTCTTTCTTGAGGAGGGCAGCAAACGGGAAGAG
AAAAG-P3	GAGGAGGGCAGCAAACGGGAAGAGTCTTCCTTTACGATATTCTTTTCTTTTCTTTTCTTTTCTTTTCTTTTCTTTTCTTTTTTGAAACAGAGTCATATAGCATTCTTTCTTGAGGAGGGCAGCAAACGGGAAGAG

Table S2.

Table HCR probe sequences used.

			Blinded Analysis #1			Blinded Analysis #2			
Sample ID	Genotype	Source	Total Cells	Positive	% Positive	Total Cells	Positive	% Positive	Avg % Positive
BN-16-198	CANVAS	MassGeneral Brigham SCiN	276	205	74.28	268	189	70.52	72.40
2008-030	CANVAS	Netherlands Brain Bank	325	165	50.77	459	179	39.00	44.88
P15-99 23	CANVAS	Queen Square Brain Bank UCL	352	77	21.88	513	74	14.42	18.15
P41-20-22	CANVAS	Queen Square Brain Bank UCL	286	3	1.05	576	0	0.00	0.52
198	C9	University of Michigan Brain Bank	363	0	0.00	546	0	0.00	0.00
1233	C9	University of Michigan Brain Bank	360	0	0.00	513	0	0.00	0.00
1875	HD	University of Michigan Brain Bank	440	0	0.00	597	0	0.00	0.00
113	HD	University of Michigan Brain Bank	529	7	1.32	740	0	0.00	0.66
02-AA-257	SCA3	University of Michigan Brain Bank	376	9	2.39	692	0	0.00	1.20
1532	Control	University of Michigan Brain Bank	300	32	10.67	380	15	3.95	7.31
T-357	Control	University of Michigan Brain Bank	384	0	0.00	430	0	0.00	0.00
1033	Control	University of Michigan Brain Bank	354	3	0.85	515	0	0.00	0.42
1522	Control	University of Michigan Brain Bank	333	2	0.60	357	0	0.00	0.30
02-AA-185	Control	University of Michigan Brain Bank	393	0	0.00	346	0	0.00	0.00
1073	Control	University of Michigan Brain Bank	440	1	0.23	558	0	0.00	0.11
382 02-AA-185	Control	University of Michigan Brain Bank	431	2	0.46	561	2	0.36	0.41
T-346	Control	University of Michigan Brain Bank	164	0	0.00	178	0	0.00	0.00
P64-11 22	Control	Queen Square Brain Bank UCL	394	90	22.84	490	65	13.27	18.05
P47-11 22	Control	Queen Square Brain Bank UCL	307	21	6.84	401	1	0.25	3.54
P38-19 22	Control	Queen Square Brain Bank UCL	370	5	1.35	492	0	0.00	0.68

Table S3.

Full blinded quantitative analysis from CANVAS, control, and disease control post-mortem cerebellum anti-KGREG IHC.

Reagent or Resource	Source	Details
**Cell Lines**		
Control 1 (2E)	Laboratory of Jack Parent, University of Michigan	Tidball et al.(*88*), Mojica-Perez at al.(*70*)
Control 2 (NL-Xa 1071)	Laboratory of Paul Hagerman, UC Davis	Liu et al.(*89*)
Control 3 (UM4-6)	MStem Cell Laboratory of Gary Smith, University of Michigan	NIH (hESC-12-0147). Haenfler et al.(*90*)
CANVAS 1P	Michigan Medicine (Dermal fibroblast patient sample)	In-house episomal hiPSC reprogramming
CANVAS 2P	Michigan Medicine (Dermal fibroblast patient sample)	In-house episomal hiPSC reprogramming
CANVAS 3P	Michigan Medicine (Dermal fibroblast patient sample)	In-house episomal hiPSC reprogramming
CANVAS 4P	Laboratory of Vikram Khurana, Harvard Medicine, (Fibroblasts)	In-house episomal hiPSC reprogramming
CANVAS 1P Isogenic	CRISPR-edited CANVAS 1P line	In-house episomal hiPSC reprogramming
CANVAS 2C Heterozygous	Michigan Medicine (Dermal fibroblast patient sample)	In-house episomal hiPSC reprogramming
**Patient Brains**		
CANVAS 1	MassGeneral Brigham SCiN	BN-16-198-7(*7*)
CANVAS 2	Netherlands Brain Bank	2008-030
CANVAS 3	Queen Square Brain Bank UCL	P15-99 23
CANVAS 4	Queen Square Brain Bank UCL	P41-20-22
C9Orf72 ALS/FTD 1	University of Michigan Brain Bank	198
C9Orf72 ALS/FTD 2	University of Michigan Brain Bank	1233
Huntington's Disease 1	University of Michigan Brain Bank	1875
Huntington's Disease 2	University of Michigan Brain Bank	113
Spinocerebellar Ataxia Type 3 1	University of Michigan Brain Bank	02-AA-257
Unaffected Control 1	University of Michigan Brain Bank	1532
Unaffected Control 2	University of Michigan Brain Bank	T-357
Unaffected Control 3	University of Michigan Brain Bank	1033
Unaffected Control 4	University of Michigan Brain Bank	1522
Unaffected Control 5	University of Michigan Brain Bank	02-AA-185
Unaffected Control 6	University of Michigan Brain Bank	1073
Unaffected Control 7	University of Michigan Brain Bank	382 02-AA-185
Unaffected Control 8	University of Michigan Brain Bank	T-346
Unaffected Control 9	Queen Square Brain Bank UCL	P64-11 22
Unaffected Control 10	Queen Square Brain Bank UCL	P47-11 22
Unaffected Control 11	Queen Square Brain Bank UCL	P38-19 22

**iPSC Reprogramming**		
pCXLE-hUL	Addgene	plasmid #27080
pCXLE-hSK	Addgene	plasmid #27078
pCXLE-hOCT3/4-shP53	Addgene	plasmid #27077
TeSR^{TM}E7^{TM} Reprogramming Media	STEMCELL Technologies	#05914

**iPSC Maintenance**		
TeSR™E8™ Maintenance Media	STEMCELL Technologies	#05990
ROCK Inhibitor (Y-27632)	Cayman Chemicals	#10005583
EDTA 0.5M	Lonza	#BMA51201
**Lentiviral Constructs**		
Lenti-EV-GFP-VSVG	University of Michigan Vector Core	100x (1x10^8 TU/mL)
Lenti-pGipZ Scramble Control	University of Michigan Vector Core	100x (1x10^8 TU/mL)
Lenti-eF1a-RFC1-3xF-GFP	VectorBuilder	100x (1x10^8 TU/mL)
Lenti-shRFC1-Exon4-GFP	Horizon Discovery	100x (1x10^8 TU/mL)
Lenti-shRFC1-Exon15-GFP	Horizon Discovery	100x (1x10^8 TU/mL)
**SMAD-Neuron Differentiation**		
Brainphys Neuronal SM1 Kit	STEMCELL Technologies	#05792
N-2 Supplement	ThermoFisher Scientific	#17502001
Laminin	Millipore Sigma	#L2020
Recombinant Human BDNF	PeproTech	#450-02
Recombinant Human GDNF	PeproTech	#450-10
Bucladesine (dbcAMP)	Cayman Chemicals	#14408
L-Ascorbic Acid	Sigma	#A4403
Accutase™	ThermoFisher	#00-4555-56

**Antibodies**		
FLAG M2	Sigma	#F1804 (1:1000 WB, 1:100 ICC)
KGREG	Abclonal	(1:500 WB, 1:100 ICC/IHC)
PFPSL	Abclonal	(1:500 WB, 1:100 ICC/IHC)
γ-H2AX	Abcam	#ab11174 (1:2000 WB, 1:500 ICC)
RFC1	GeneTex	#GTX129291 (1:2000 WB)
β-Tubulin	DSHB	#E7 (1:1000 WB)
Oct4	Abcam	#ab181557 (1:250 ICC)
Nanog	Biolegend	#674202 (1:250 ICC)
NeuN	MilliporeSigma	#MAB377 (1:500 ICC)
CHL1	Abcam	#ab106269 (1:500 WB)
CAMKIIB	Fisher	#13-9800 (1:1000 WB)
Synaptophysin	Abcam	#ab32127 (1:1000 WB)
SHANK1	Novus	#NB300-167 (1:1000 WB)
GAP43	Fisher	#33-5000 (1:2000 WB)
AlexaFluor Rabbit-488	ThermoFisher	#A-11008 (1:1000 ICC 2°)
AlexaFluor Rabbit-555	ThermoFisher	#A-21428 (1:1000 ICC 2°)
AlexaFluor Mouse-488	ThermoFisher	#A-11001 (1:1000 ICC 2°)
AlexaFluor Mouse-555	ThermoFisher	#A-21422 (1:1000 ICC 2°)
IRDye Rabbit-680rd	LI-COR	#926-68071 (1:5000 WB 2°)
IRDye Rabbit-800cw	LI-COR	#926-32211 (1:5000 WB 2°)

IRDye Mouse-680rd	LI-COR	#926-68070 (1:5000 WB 2°)
IRDye Mouse-800cw	LI-COR	#926-32210 (1:5000 WB 2°)

Table S4.
Table of information for all cell lines, patient samples and reagents used in these studies

Movie S1.

Representative calcium imaging time course movie for human control neurons over a duration of 180s.

Movie S2.

Representative calcium imaging time course movie for human CANVAS neurons over a duration of 180s.

Movie S3.

Representative calcium imaging time course movie for CANVAS isogenic neurons over a duration of 180s.

Fig. S7A Uncropped Blots

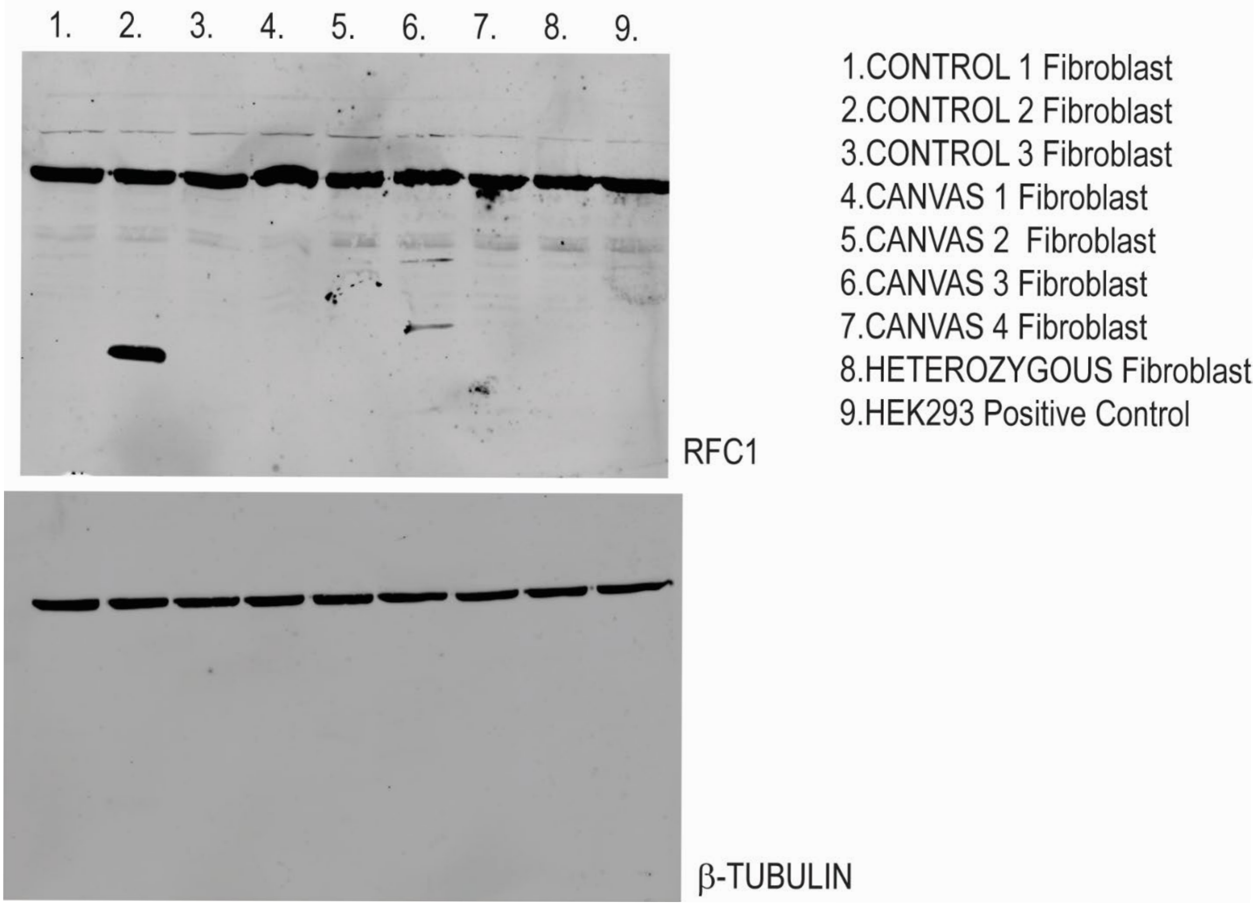

Supplement: Supplementary file 4 [file sciadv.adn2321_sm.v2.pdf]
